# Reproducibility of drug-induced effects on the contractility of an engineered heart tissue derived from human pluripotent stem cells

**DOI:** 10.3389/fphar.2023.1212092

**Published:** 2023-07-04

**Authors:** Ayesha Arefin, Melissa Mendoza, Keri Dame, M. Iveth Garcia, David G. Strauss, Alexandre J. S. Ribeiro

**Affiliations:** ^1^ Division of Applied Regulatory Science, Office of Clinical Pharmacology, Office of Translational Sciences, Center for Drug Evaluation and Research, U.S. Food and Drug Administration, Silver Spring, MD, United States; ^2^ Division of Systems Biology, National Center for Toxicological Research, U.S. Food and Drug Administration, Jefferson, AR, United States; ^3^ Office of Clinical Pharmacology, Office of Translational Sciences, Center for Drug Evaluation and Research, U.S. Food and Drug Administration, Silver Spring, MD, United States

**Keywords:** microphysiological systems, pluripotent stem cells, cardiotoxicity, contractility, drug development

## Abstract

**Introduction:** Engineered heart tissues (EHTs) are three-dimensional culture platforms with cardiomyocytes differentiated from human pluripotent stem cells (hPSCs) and were designed for assaying cardiac contractility. For drug development applications, EHTs must have a stable function and provide reproducible results. We investigated these properties with EHTs made with different tissue casting batches and lines of differentiated hPSC-cardiomyocytes and analyzed them at different times after being fabricated.

**Methods:** A video-optical assay was used for measuring EHT contractile outputs, and these results were compared with results from motion traction analysis of beating hPSC-cardiomyocytes cultured as monolayers in two-dimensional cultures. The reproducibility of induced contractile variations was tested using compounds with known mechanistic cardiac effects (isoproterenol, EMD-57033, omecamtiv mecarbil, verapamil, ranolazine, and mavacamten), or known to be clinically cardiotoxic (doxorubicin, sunitinib). These drug-induced variations were characterized at different electrical pacing rates and variations in intracellular calcium transients were also assessed in EHTs.

**Results:** To ensure reproducibility in experiments, we established EHT quality control criteria based on excitation-contraction coupling and contractile sensitivity to extracellular calcium concentration. In summary, a baseline contractile force of 0.2 mN and excitation-contraction coupling of EHTs were used as quality control criteria to select suitable EHTs for analysis. Overall, drug-induced contractile responses were similar between monolayers and EHTs, where a close relationship was observed between contractile output and calcium kinetics. Contractile variations at multiple time points after adding cardiotoxic compounds were also detectable in EHTs.

**Discussion:** Reproducibility of drug-induced effects in EHTs between experiments and relative to published work on these cellular models was generally observed. Future applications for EHTs may require additional mechanistic criteria related to drug effects and cardiac functional outputs to be measured in regard to specific contexts of use.

## 1 Introduction

Engineered heart tissues (EHTs) are three-dimensional (3D) *in vitro* cellular platforms containing cardiomyocytes (Schaaf et al., 2011) differentiated from human pluripotent stem cells (hPSC-cardiomyocytes) (Yamanaka, 2007; Burridge et al., 2012) and have demonstrated potential to predict cardiac drug effects from variations in their assayable functional outputs (Matsa et al., 2014; Denning et al., 2016; Ribeiro et al., 2019). In these culture platforms, interconnected hPSC-cardiomyocytes forming a beating tissue are aligned between load-bearing deflectable micropost force sensors (Eschenhagen et al., 2012) and can be assayed to provide functional results on drug-induced cardiac contractile effects (Jacob et al., 2016; Mannhardt et al., 2016; Mannhardt et al., 2017; Saleem et al., 2020; Rhoden et al., 2022). Despite being primarily developed to determine cardiac contractility from the deflection of microposts (Hansen et al., 2010), other cardiac properties have been assayed with EHTs, such as electrophysiology (Eder et al., 2014), calcium cycle (Stoehr et al., 2014), and metabolism and mitochondrial function (Rhoden et al., 2022). Since adverse cardiac events are among the main causes for drug attrition (Batta et al., 2020), new *in vitro* models like the EHT are sorely needed to predict such events in the early stages of drug development (Pang et al., 2019). To eventually be used as robust drug development tools (Baran et al., 2022), EHTs must provide reproducible results. In addition, the advantages of EHTs in predicting clinical cardiac effects, when compared with monolayers of hPSC-cardiomyocytes in two dimensional (2D) cultures (da Rocha et al., 2017), are still not clear. Here, the reproducibility of drug-induced effects on EHT contractility was studied (Schaaf et al., 2011), while aiming to 1) follow quality control characteristics based on established functional specifications that may ensure reproducibility; and 2) test the role of critical EHT-based assay properties (i.e., origin of cells, rate-dependent stimulation, 3D vs. 2D, and contractility vs. calcium functional outputs) on the reproducibility of measured drug-induced effects.

Reproducibility of results is key for developing drug development tools (Kannt and Wieland, 2016). The potential of EHTs as being able to predict cardiac drug effects has been demonstrated since the first prototypes were published over a decade ago (Hansen et al., 2010; Stoehr et al., 2014). For example, EHTs have reproduced clinically relevant cardiac drug effects (Schaaf et al., 2011), and clinically-relevant effects of cardiac ion-channel modulatory compounds have been assayed with EHTs (Mannhardt et al., 2016). Poor reproducibility of such type of proof-of-concept studies has been generally identified as one of the main causes for failure in method translation (Kannt and Wieland, 2016), as needed for establishing drug development tools (Kraus, 2018). It has been reported that two-thirds of published data was difficult to reproduce upon investigating over 50 drug discovery projects (Prinz et al., 2011). In addition, reproducibility of published experiments has been identified as a key enabler in the translation of new drug development tools, along with publishing protocols, materials, and methods with full transparency, displaying quality control attributes, and ensuring clear communication to avoid data ambiguity (Kannt and Wieland, 2016). Specifically for cell-based platforms, quality control criteria have also been noted to ensure their biological reproducibility and therefore the reliability of generated data (Zhang et al., 2012; Chen et al., 2016). Since improper fabrication or handling procedures can negatively affect EHT performance, we hypothesized that following quality control criteria for baseline EHT function could aid in ensuring the reproducibility of drug-induced responses of EHTs (Chen et al., 2016). To test this hypothesis, assayable criteria based on expected cardiac physiology related to EHT contractility were investigated.

As an assayable output of cardiac function, contractility is the outcome of a series of molecular events that lead to actin-myosin interactions and depend on coordinated events related to electrophysiology, calcium cycling and mitochondrial activity (Ribeiro et al., 2019). Therefore, variations in contractility can generally reflect changes in the diverse mechanisms that regulate it and a wide range of drug-induced effects can be detected with contractility assays (Abi-Gerges et al., 2020). Test compounds with distinct mechanisms of action on cardiac-specific pathways are often used in cell-based assays to evaluate how robustly specific types of effects can be predicted. Being contractility also a minimally invasive and non-destructive assay facilitates running quality control trials and drug testing at multiple time-points, thus enabling an integrated characterization of the reproducibility of baseline function and drug-induced effects. In summary, this work builds on well-established EHT contractility assays (Mannhardt et al., 2016) and proposes following quality control criteria around established specifications to ensure data reproducibility.

## 2 Materials and methods

A detailed materials and methods document is provided as Supplementary Material. Upon fabricating EHTs, we tested the ability of these tissues to robustly reproduce published data on their baseline contractility and drug-induced variations. This characterization involved a phased approach with distinct experiments focused on contractility endpoints and using test compounds with known contractile effects (Guth et al., 2019; Ribeiro et al., 2019) or known to be cardiotoxic (Mamoshina et al., 2021).

### 2.1 Thawing and maintenance of cryopreserved hPSC-Cardiomyocytes

EHTs were fabricated with two different lines of cardiomyocytes differentiated from hPSCs: commercially available iCell Cardiomyocytes2 (Fujifilm Cellular Dynamics) and in-house differentiated human WTC-11 GCaMPf hPSC line (Maddah et al., 2015), which was a gift from Dr. Bruce Conklin (Gladstone Institute of Cardiovascular Disease and UCSF). Prior to use in EHT fabrication, cells were maintained in liquid nitrogen upon receiving from the respective vendor (iCell Cardiomyocytes2) or after differentiation (WTC-11 GCaMPf hPSC-cardiomyocytes). Detail on these procedures, including on the differentiation of WTC-11 GCaMPf hPSCs, is included in the supplementary detailed materials and methods document. In summary, cardiomyocytes were thawed and counted to ensure that each EHT was loaded with 1.1 million cells or that 2D culture platforms were seeded with 156,000 viable cells/cm2. WTC-11 GCaMPf hPSCs were differentiated into cardiomyocytes using a protocol based on temporal modulation of WNT signaling (Lian et al., 2012), which was then followed by a purification step with metabolic selection at day 11 after the onset of differentiation (Tohyama et al., 2013). On day 28, differentiated cardiomyocytes were dissociated using accutase (Innovative Cell Technologies, Inc.) and cryopreserved in 90% FBS (ThermoFisher Scientific)/10% dimethyl sulfoxide (Sigma-Aldrich).

### 2.2 Baseline characterization of EHT contractility

EHTs were fabricated and maintained following the vendor’s recommendations (DiNAQOR Deutschland GmbH) (Mannhardt et al., 2016). As detailed ahead in the results section, EHT functional properties to assess quality control were tested. Tissues that showed an irregular/asynchronous beating pattern on day two or three after fabrication and had developed a synchronized beating pattern by day seven were used in experiments. In addition, once EHT contractility was stable, its sensitivity to varying concentrations of extracellular calcium (0.063 mM–2 mM calcium in Tyrode’s solution) and rate-dependent variations were tested. Contractile sensitivity to extracellular calcium was investigated at 1.25 Hz electrical pacing rate. To acquire rate-dependent measurements, EHTs were incubated with 300 nM of ivabradine in EHT medium for 1 hour (Mannhardt et al., 2016) and, after incubation, contractility was measured without any pacing and thereafter, at 0.5 Hz and at higher pacing rates: 0.75, 1, 1.25, 1.5, 1.75, 2 Hz.

### 2.3 EHT quality control criteria used prior to drug exposure

After 2 weeks of EHT maintenance, follow-on experiments with compounds were continued with EHTs that produced an average force of 0.2 mN. If average force did not reach 0.2 mN after one or two additional weeks of the being characterized, EHTs were discarded. A second criterion for assessing quality was based on the ability of EHTs to beat at the same rate of the electrical pacing frequency: 0.5, 1 and 2 Hz. EHTs were used up to 1 week after ensuring these quality performance criteria.

### 2.4 Frequency-dependent effects of compounds known to increase/decrease contractility in EHTs developed from two different HPSC lines

EMD57033, omecamtiv mecarbil, verapamil, and mavacamten were added to EHTs to investigate if frequency-dependent variation in contractility could be reproduced in both types of EHTs. Responses were recorded with two pacing rates (0.5 Hz, 1 Hz) for EMD57033 and omecamtiv mecarbil. EHTs were incubated in 300 nM of ivabradine for 1 hour and a baseline measurement was done before testing the effects of compounds (Mannhardt et al., 2016). Tissue responses were recorded at 0.5 and 1 Hz pacing rates in Tyrode’s solution for different drug concentrations. Responses to verapamil and mavacamten were measured at a pacing rate of 1.25 Hz.

### 2.5 Comparing the contractile effects of compounds between EHTs and monolayers of HPSC-Cardiomyocytes

The contractile effects of isoproterenol, verapamil, ranolazine, and aspirin (control) were investigated in both EHTs and monolayers containing iCell cardiomyocytes2. Culture, electrical stimulation, and drug exposure conditions were kept the same between EHTs and monolayers. The contractility of the cellular monolayer was determined with a motion detection assay using a SI8000 Cell Motion Imaging System (Sony Biotechnology Inc., San Jose, CA, USA) (Kopljar et al., 2017), while using the EHT measuring system to characterize EHT contractility (Mannhardt et al., 2017). Drug concentrations and exposure schedules were identical for both EHTs and monolayers and baseline measurements were done before and after incubating with 300 nM of ivabradine for 1 hour as detailed in the previous sections for EHTs.

### 2.6 Assaying drug-induced variations in the kinetics of intracellular calcium in EHTs

EHTs were fabricated with cardiomyocytes differentiated from hPSCs expressing a GCaMP6f calcium indicator (Chen et al., 2013). Experiments were designed to investigate variations in the kinetics of intracellular calcium transients and contractility upon adding EMD57033, omecamtiv mecarbil, verapamil, and mavacamten as already described. This approach involved video acquisition with an inverted fluorescent microscope, and electrical pacing.

### 2.7 Testing long-term cardiotoxic effects of compounds with EHTs

Sub-acute or delayed contractile effects due to cardiotoxic drug exposure of sunitinib, doxorubicin, and erlotinib (control), and recovery back to baseline levels were investigated. Spontaneous contractility was measured before, during and after drug exposures. EHTs generated from iCell cardiomyocytes2 were maintained for 21 days or one additional week until EHTs produced an average force of 0.2 mN. These were then maintained with daily medium changes in 4% serum for 5 days and in serum-free medium for one additional day before drug exposure in serum-free medium for 2 days. After exposure to drugs, tissues were maintained in 4% serum for another 5 days with daily medium changes and functional characterization.

### 2.8 Parameters derived from video-based data analysis

Video-based approaches involved analyzing sequences of frames to determine functional parameters that relate to contractile function or intracellular calcium transients. From the EHT testing systems (DiNAQOR Deutschland GmbH), the following parameters were obtained to characterize the average of the recorded contractile cycles: frequency of contractions, force of contraction, time parameters describing the time to peak (RT) or time from peak (RT) involved in each segment from 10% to 90% of the peak of the contraction cycle or during relaxation [TTP (−10%), TTP (−20%), TTP (−50%), TTP (−80%), TTP (−90%); RT (10%), RT (20%), RT (50%), RT (80%), RT (90%)], contraction velocity, relaxation velocity. Parameters resultant from analyzing videos of beating hPSC-cardiomyocytes monolayers (Sony Biotechnology Inc., San Jose, CA, USA) were beating area, frequency of contractions, contraction velocity, relaxation velocity, contraction-end velocity, acceleration, contraction deformation distance, relaxation deformation distance, contraction duration, relaxation duration, contraction-relaxation duration, contraction-relaxation peak interval. MATLAB-based video analysis of fluorescent calcium probes yielded intensity variations, peak magnitude of intensity variation, rate of fluorescent signal rise, rate of fluorescent signal decay, contraction time and frequency of contractions.

### 2.9 Data analysis

The statistical significance of test results in relation to control conditions was evaluated using paired *t*-test for the group data. Ordinary one-way ANOVA with Dunnett’s multiple comparison test was performed for mixed model. Ordinary two-way ANOVA with Dunnett’s multiple comparison test, with individual variances were used for mixed models. Data were presented as means ± SD. *p*-values compared to control were included.

## 3 Results

We tested the reproducibility of baseline EHT contractility and drug-induced contractile variations, while also testing the physiological relevance of EHT contractile function and following quality control criteria for these cellular models (Ribeiro et al., 2019). Overall, reproducibility of contractility output was investigated with experiments using distinctly fabricated batches of EHTs or with two different lines of hPSC-cardiomyocytes. EHTs were exposed to extracellular stimuli, such as varying extracellular calcium concentration and electrical stimulation (Wiegerinck et al., 2009), or compounds with known effects on contractility, specifically omecamtiv mecarbil (Saleem et al., 2020), EMD57003 (Saleem et al., 2020), verapamil (Mannhardt et al., 2016), mavacamten (Feric et al., 2019), isoproterenol (Saleem et al., 2020), ranolazine (Blinova et al., 2018), or cardiotoxic drugs. Specifically erlotinib (Jacob et al., 2016), doxorubicin (Hansen et al., 2010), and sunitinib (Truitt et al., 2018) were used.

### 3.1 Contractility of EHTs following quality criteria is stable beyond 80 days in culture

**FIGURE 1:**
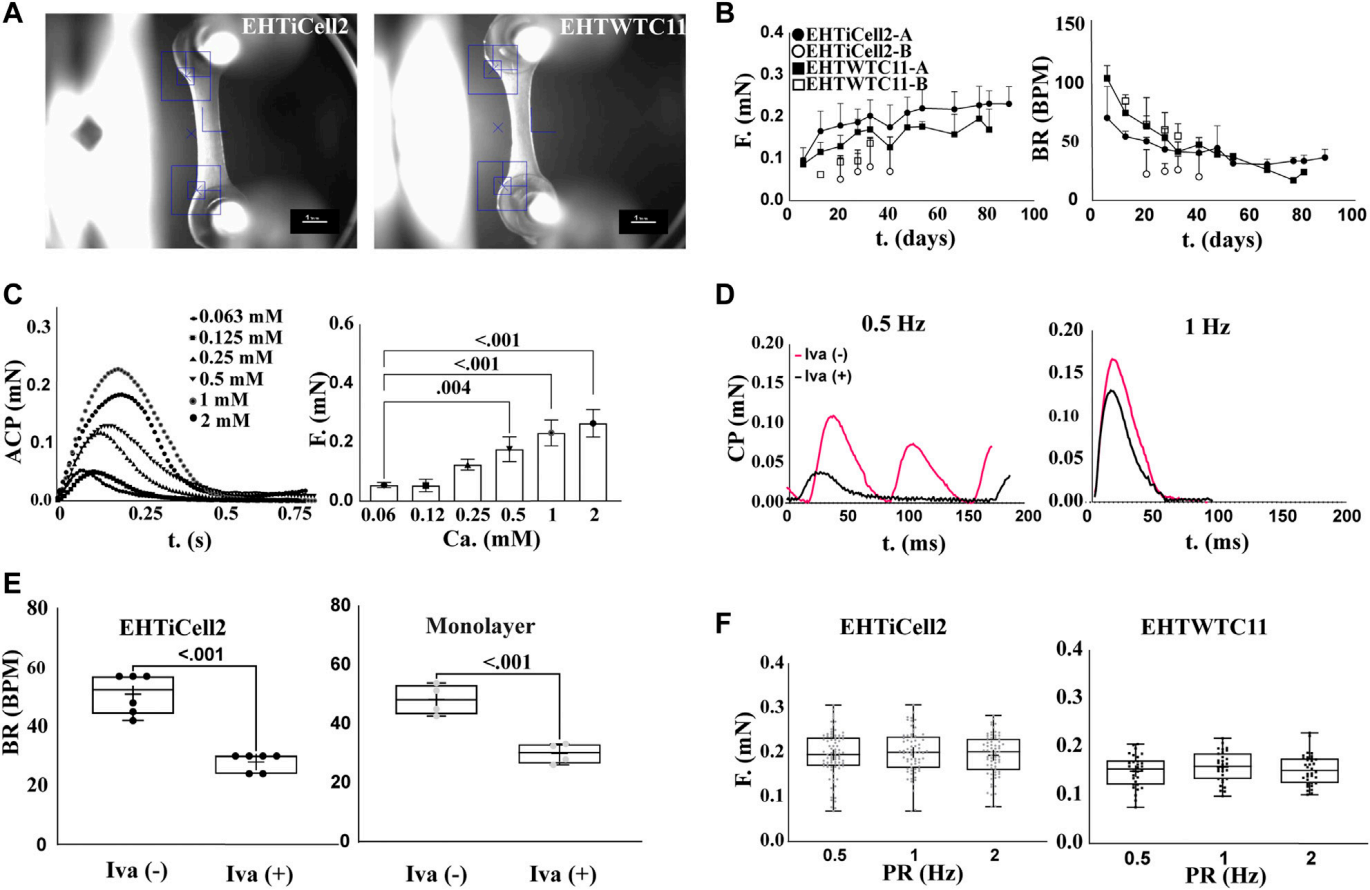
Contractile function of EHTs following quality criteria are stable beyond 80 days. **(A)** Representative brightfield image of EHTs generated from iCell Cardiomyocytes2 (EHTiCell2) (left), and hPSC-cardiomyocytes expressing GCaMPf (EHTWTC11) (right). Scale bar = 1 mm. **(B)** Force and frequency of spontaneously beating tissue were recorded over 80 days. EHTiCell2-A, n = 6, EHTWTC11-A, n = 14, EHTiCell2-B, n = 5, EHTWTC11-B, n = 8. Data presented as mean ± SD. **(C)** Effect of external calcium concentrations on contractile parameters measured in EHTiCell2. Left shows average contraction peaks, and right represents mean force upon electrically pacing EHTiCell2s at 1.25 Hz in Tyrode’s solution with different calcium concentrations. Number of experiments (N) = 1, replicates (n) = 3. Ordinary one-way ANOVA was performed, adjusted *p*-value shown. **(D)** Effect of ivabradine on EHTs’ ability to follow electrical pacing. Average contraction peaks obtained from pacing EHTiCell2s at 0.5 (left) and 1 Hz (right) in modified Tyrode’s solution with 0.6 mM calcium. Tissues were incubated with 300 mM of ivabradine for 1 hour prior to electrical pacing. **(E)** Effect of ivabradine on beating frequency in monolayers, and in EHTiCell2s. Measurements obtained from monolayers (n = 4) and EHTs (n = 6) with or without being pre-incubated in 300 mM ivabradine for 1 hour. Paired *t*-test was performed, *p*-values are shown in the graph. **(F)** Force-frequency relationship of electrically paced EHTs after incubation in ivabradine. n = (83, 70, 69) for EHTiCell2s, and n = (30, 30, 34) for EHTWTC11. Ordinary one-way ANOVA was performed. EHTiCell2-A and B (represents tissues from fabrication batch 4 and 8), EHTWTC11-A and B (represents tissues from fabrication batch 3 and 5), F = force, BR = beat rate, T = time, ACP = average contraction peak, CP = contraction peak, Iva (−) = without ivabradine, Iva (+) = with ivabradine, PR = pacing rate.

Figure 1A shows the representative morphology of an EHT (Hansen et al., 2010; Breckwoldt et al., 2017), where cells self-organized into a beating tissue between microposts within a fibrin-based matrix under tension (Supplementary Video S1). Quality control criteria used early on the first week after fabricating EHTs consisted of having a homogeneous distribution of cells between microposts, and asynchronous beating within two or 3 days of fabrication (Supplementary Video S2) (Hansen et al., 2010). After 2 weeks, quality criteria for EHTs focused in observing 1) forces from spontaneous contractions in the range of 0.2 mN (20% within this value: Supplementary Tables S1, S2), 2) synchronous beating (Supplementary Videos S3, S4), and 3) beating at rate similar to the frequency of electrical stimulation when paced (Mannhardt et al., 2016; Breckwoldt et al., 2017). Overall, a total of 17 fabrication batches of EHTs with iCell Cardiomyocytes2 hPSCs (EHTiCell2) followed these quality control criteria (Supplementary Table S3). EHTiCell2 following quality criteria at week three after fabrication (Figure 1B, and Supplementary Tables S1, S2) produced an average contractile force of 0.21 ± 0.04 mN (n = 87) (Figure 1B, left) at a spontaneous beating frequency of 43 ± 9 (n = 70) beats per minute (Figure 1B, right). In total, 77% ± 32% of fabricated EHTiCell2s from the 17 independent fabrication batches followed quality control criteria. Also, the average spontaneous contraction force remained between 80% and 120% of 0.2 mN up to 90 days (Supplementary Table S2 and Supplementary Figure S1B). Changes were observed in other contractile parameters over time, including an increase in contraction and relaxation velocities (Supplementary Figures S1E, F) In summary, EHTiCell2s that followed the described quality criteria showed reproducible and robust spontaneous baseline contractility for weeks after fabrication.

To test the physiological relevance of EHT contractility in relation to followed quality criteria, effects induced by varying concentrations of extracellular calcium (Kitazawa, 1984) under electrical pacing were investigated. Overall, the magnitude of the contractile output of EHTiCell2s was proportional to extracellular calcium concentration (Figure 1C). The observed half maximal effective concentration for the contractile effects of extracellular calcium was 0.40 mM (Supplementary Figure S2, n = 3, HillSlope = 2.065). No concentrations were tested above 2 mM given the observed unstable beat rates at this range, despite electrical pacing (Supplementary Figure S3). Other contractile properties varied with extracellular calcium, including contraction time, and contraction and relaxation velocities (Supplementary Figure S4). Spontaneous beat rate was partially inhibited in EHTs with ivabradine and beat rate followed both tested pacing frequencies of 0.5 Hz (Figure 1D, left), and 1 Hz (Figure 1D, right). Electrically pacing EHTs not treated with ivabradine was possible when the pacing frequency was higher than their spontaneous beat rate (Figure 1D, right) (Lemoine et al., 2018). Both cardiomyocyte monolayers and EHTs were treated with ivabradine to decrease the rate and magnitude of spontaneous contractions (Figure 1E), thus allowing a better control of their beating frequency (Mannhardt et al., 2016). The force-frequency relationship in ivabradine-treated EHTs were investigated to further test their physiological relevance (Endoh, 2004 et al., 2009), since the cardiac contractile output is known to be rate dependent, and drug effects can be rate-dependent (Butler et al., 2015). No variation in the force of contractions was observed as a function of beat rate (Figure 1F, left). In summary, EHTs following quality control criteria presented contractile outputs that were proportional to extracellular calcium concentration and their frequency easily followed electrical pacing rate upon incubation with ivabradine.

EHTWTC11s were generated from cardiomyocytes differentiated in house from WTC11 hPSCs expressing a GCaMP6f calcium indicator and were also characterized to investigate potential effects of a different source of cells on the reproducibility of EHT results. Figure 1A-right shows a representative image of EHTWTC11s, where no morphological differences were detected between these and EHTiCell2s shown in Supplementary Video S5. A total of five batches of EHTWTC11s were fabricated and experiments were also performed with those that followed the already noted performance criteria (Supplementary Table S4). Spontaneous contractions of EHTWTC11 generated an average force of 0.16 ± 0.01 mN force (n = 26) at 50 ± 5 (n = 26) beats per minute within 4 weeks of fabrication (Figure 1B) and maintained their force output between 70% and 110% of 0.2 mN from day 14 to day 82 (Supplementary Table S2). Varying beat rate had similar effects in both EHT types, where no variation in force was detected (Figure 1F, right). Overall, a 0.2 mN force was achieved earlier in EHTiCell2 (2 weeks after fabrication) and later in EHTWTC11 (4 weeks after fabrication) (Supplementary Tables S1, S2). A reduction in spontaneous beat rate systematically occurred after several weeks of culturing EHTs: 66% decrease in EHTWTC11 (Supplementary Figure S5A) and 31% decrease in EHTiCell2 (Supplementary Figure S1A). A longer relaxation time and slower relaxation velocity were observed in EHTWTC11 compared to EHTiCell2 (Supplementary Figures S1, S5, S6). EHTWTC11 had shorted resting lengths than EHTiCell2 (Supplementary Figure S7), suggesting that a higher tissue tension was achieved when using the differentiated cardiomyocytes from WTC-11 GCaMPf hPSCs. Taken together, these data strongly suggested that EHTs following performance criteria had a stable and robust contractile function (Supplementary Table S5) and that EHTs fabricated with different lines of hPSC-cardiomyocytes can present distinct baseline properties.

### 3.2 Drug-induced contractile effects of EMD57033 and omecamtiv mecarbil on EHTs

**FIGURE 2:**
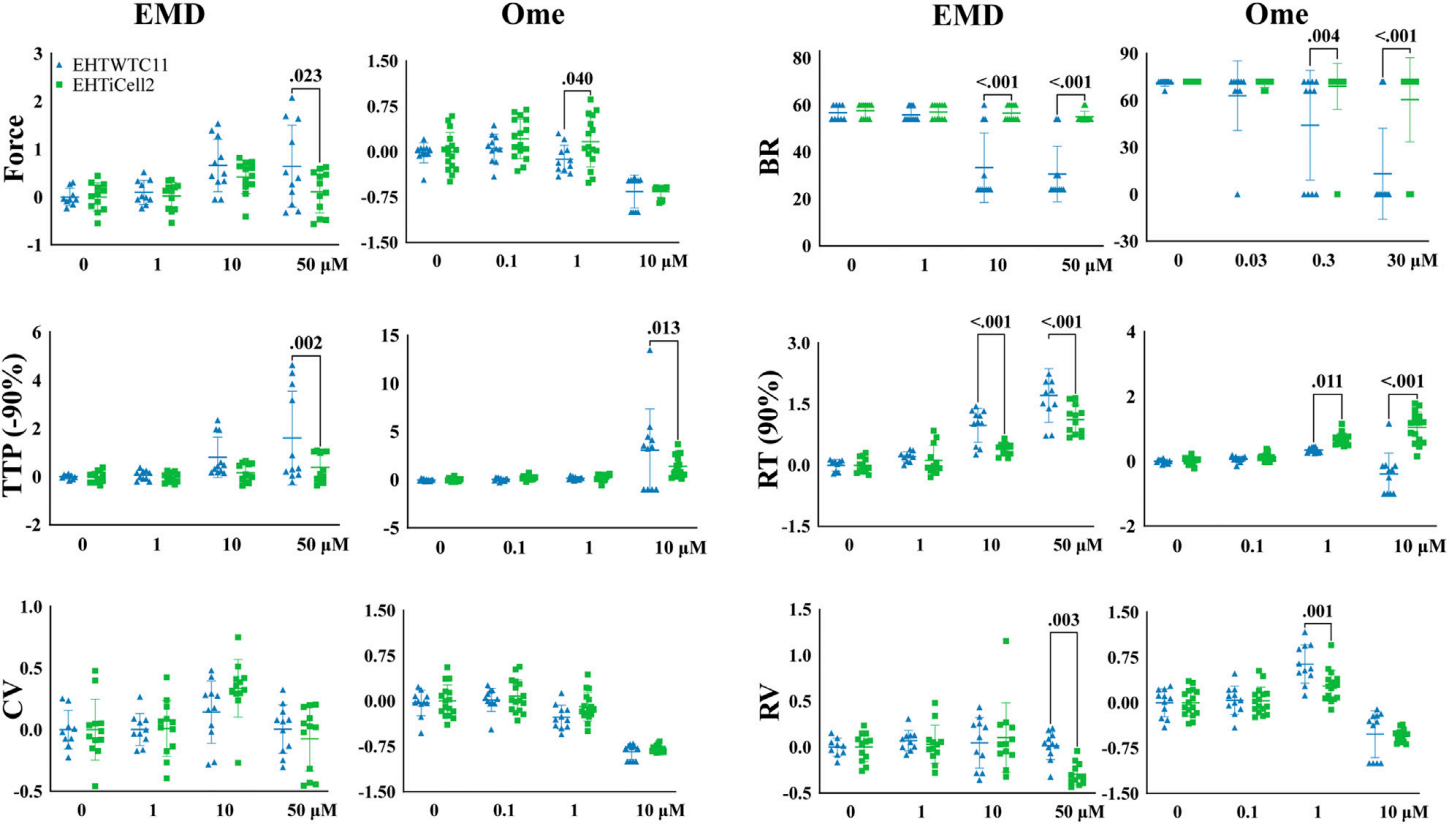
Cell line dependent effects of compounds known to increase contractility in EHTs. Effects of EMD57033 and omecamtiv mecarbil on contractility were measured. Data collected from EHTs generated from iCell Cardiomyocytes2 (EHTiCell2) and from hPSC-cardiomyocytes expressing GCaMPf (EHTWTC11). Contractility was analyzed at a 1 Hz pacing rate in modified Tyrode’s solution with 0.6 mM calcium. EHTs were pre-incubated for 1 hour in 300 nM of ivabradine. Data represents values normalized to the mean except BR, ±SD, *p*-values were determined by ordinary two-way ANOVA using Sidak’s multiple comparison test, with a single pool variance. Number of experiments (N) and replicates (n) for EMD57003 were N = 5 and n = 12 with EHTiCell2s, N = 3 and n = 11 with EHTWTC11s; for omecamtiv mecarbil were N = 4 and n = 16 with EHTiCell2s, N = 3 and n = 11 with EHTWTC11s. EMD = EMD57003, Ome = omecamtive mecarbil, BR = Beat rate, TTP (-90%) = time to peak from 90% contraction, CV = contraction velocity, RT (90%) = time to peak from 90% relaxation, RV = relaxation velocity. 3 vials of iCell Cardiomyocytes2 and 2 vials of WTC11 hPSC-cardiomyocytes were used in these experiments.

EMD57033 and omecamtiv mecarbil increase the contractile output of the myocardium (Brixius et al., 2002) and EHTs were exposed to different concentrations of these compounds to test their dose-dependent effects. Since these compounds have rate-dependent effects (Wiegerinck et al., 2009), contractility was assayed at 0.5 Hz and 1 Hz in both EHTiCell2 and EHTWTC11 (Figure 2). Overall, differences were observed in the magnitude of contractile effects of EMD57033 and omecamtiv mecarbil in force, beat rate, TTP (-90%), RT (90%) and relaxation velocity between the fabricated types of EHTs, despite similar trends in the variation of these parameters between EHTiCell2 and EHTWTC11. Effects of EMD57033 were mostly observed at 50 μM, where EHTWTC11 had a higher force output than EHTiCell2, as well as other contractile parameters noted in Figure 2. In addition, almost a five-fold increase occurred in TTP (-10%) with EHTiCell2 and TTP (–20%) with EHTWTC11 (Supplementary Figure S8 and Supplementary Table S6). A consistent increase in contraction time in both EHTiCell2 and EHTWTC11 (except TTP (-10%) in EHTWTC11) occurred at 0.5 Hz of pacing frequency with EMD57033. As also shown in Supplementary Table S7, relaxation time increased in both EHT types. Higher relaxation velocity with EHTWTC11 at 0.5 Hz differed from a decrease in this parameter with EHTiCell2 at 1 Hz (Figure 2, Supplementary Figure S8 and Supplementary Table S8). EHTWTC11 paced at 0.5 Hz showed a distinct rise in contraction velocity (Supplementary Figure S8 and Supplementary Table S8). Below 50 μM, increased relaxation time RT (80% and 90%) was also evident in EHTWTC11 at 0.5 Hz with 1 µM (Supplementary Figure S8 and Supplementary Table S7). Changes in beat rate were observed in EHTWTC11 at 1 Hz, where it decreased at 10 and 50 µM (Figure 2 and Supplementary Table S9). In summary, EMD57033 augmented contractile output in both EHTiCell2 and EHTWTC11, where differences in some parameters were evident.

Omecamtiv mecarbil also showed rate- (0.5 Hz vs. 1 Hz), cell type- (iCell2-derived vs. WTC11-derived) and concentration-dependent effects on EHTs (1 µM vs. 10 µM) (Supplementary Figure S8). At 1 Hz (Figure 2), force contractile output decreased at a lower concentration of 1 µM in EHTWTC11, no effect was observed at 0.5 Hz or with 1 µM (Supplementary Figure S10 and Supplementary Table S10), and no variations occurred in resting tissue length in both EHT types at any beating rate (Supplementary Figure S7). Higher relaxation velocities were induced at 1 µM (except for EHTiCell2 at 0.5 Hz), while decreasing at 10 µM (except for EHTWTC11) (Supplementary Figure S8 and Supplementary Table S10). 10 μM further prolonged relaxation time parameters, except in EHTWTC11 at 1 Hz, with no change at RT (10, 80% and 90%) (Supplementary Figure S8 and Supplementary Table S11), and contraction time TTP (-50, −80, −90%) was prolonged in both EHTiCell2 and EHTWTC11 at 10 µM (Supplementary Figure S8 and Supplementary Table S12). Except for lower contraction velocities in EHTWTC11 at 1 Hz, 1 µM did not induce any additional changes in this parameter (Supplementary Figure S8 and Supplementary Table S13). In summary, different concentrations of omecamtiv mecarbil impacted contractile force and relaxation velocity, but with different trends at 1 μM and 10 µM. Increased contraction time and reduced contraction velocity were observed mostly at 10 µM. In addition, changes in relaxation time at 10 µM were more significant at 0.5 Hz.

### 3.3 Similar contractile effects of verapamil and mavacamten in EHTs with different lines of HPSC-Cardiomyocytes

**FIGURE 3:**
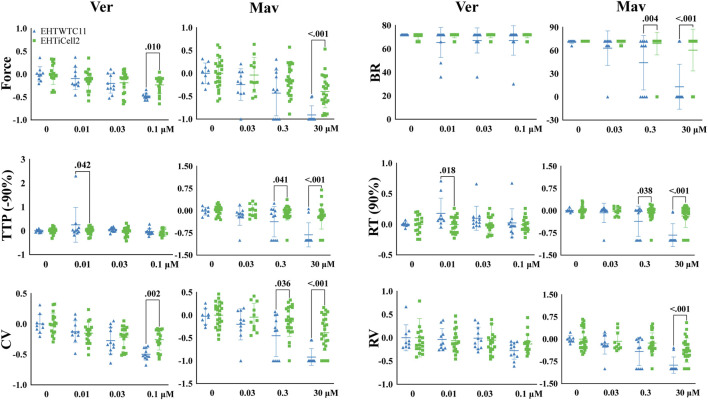
Effects in EHTs of compounds known to decrease contractility. Effects of verapamil and mavacamten on contractility were measured. Drug-induced responses were acquired at 1.25 Hz pacing rate in modified Tyrode’s solution with 0.6 mM calcium using EHTs generated from iCell cardiomyocytes2 (EHTiCell2), and EHTs generated from hPSC-cardiomyocytes expressing GCaMPf (EHTWTC11). Presented results were normalized to the mean, except with BR, ±SD, *p*-values were determined by ordinary two-way ANOVA using Sidak’s multiple comparison test, with a single pool variance. Number of experiments (N) and replicates (n) for verapamil incubations: N = 4 and n = 18 with EHTiCell2s, N = 3 and n = 11 with EHTWTC11s. For mavacamten incubations: N = 5 and n = 11–25 with EHTiCell2s, N = 3 and n = 7–11 with EHTWTC11s. Ver = verapamil, Mav = mavacamten, BR = Beat rate, TTP (-90%) = time to peak from 90% contraction, CV = contraction velocity, RT (90%) = time to peak from 90% relaxation, RV = relaxation velocity. 3 vials of iCell Cardiomyocytes2 and 2 vials of WTC11 hPSC-cardiomyocytes were used in these experiments.

Verapamil (Mannhardt et al., 2016) and mavacamten (Sewanan et al., 2021) reduce the cardiac contractile output through distinct biological mechanisms and were tested with EHTs to identifying such effects. Verapamil reduced force at the lowest tested concentration of 0.03 µM in EHTiCell2s and at 0.1 µM in EHTWTC11s (Figure 3 and Supplementary Table S14). This compound also decreased TTP (-10, and −20%) in EHTiCell2s at 0.1 µM (Supplementary Figure S9 and Supplementary Table S14). With EHTWTC11s, an increase in relaxation time parameters was observed at the tested concentrations (Supplementary Figure S9 and Supplementary Table S15). Effects on RT (80% and 90%) occurred with 0.01 µM (Supplementary Table S15). 0.1 µM verapamil caused a reduction in contraction velocity in both EHT types (Supplementary Figure S9 and Supplementary Table S16). Similar effects in contraction velocity were observed at lower concentrations with EHTiCell2s, but not with EHTWTC11s. No effects were detected on relaxation velocity (Supplementary Figure S9) or beat rate (Figure 3). Overall, verapamil reduced force and increased contraction velocity in EHTs, but at different concentrations depending on the used line of hPSC-cardiomyocytes.

Mavacamten caused a reduction in contraction force at 3 µM in both EHTiCell2s and EHTWTC11s (Figure 3 and Supplementary Table S17). At the earliest time parameter derived from the contraction peak TTP (-10%), a decrease was observed at 0.3 µM for EHTiCell2s, and at 3 µM for EHTWTC11s (Supplementary Figure S9 and Supplementary Table S17). Additional effects of 3 µM of mavacamten on TTP (-50, −80, and −90%) were similar between EHTiCell2s and EHTWTC11s. Mavacamten also reduced relaxation time, contraction velocity and relaxation velocity, with divergent concentration-dependent effects between EHTiCell2s and EHTWTC11s. Between both EHT types, a decrease in relaxation parameters was observed only at RT (20%) with 3 µM in EHTiCell2s (Supplementary Table S17). At 0.3 µM, EHTWTC11 contractions had lower RT (80% and 90%), and at 3 µM all relaxation time parameters also decreased. Contraction velocity was reduced more significantly in EHTWTC11s than in EHTiCell2s at 3 μM, but 0.3 µM had no effect on this parameter in EHTiCell2s, while reducing it in EHTWTC11 (Figure 3 and Supplementary Table S18). Relaxation velocity decreased at 3 µM in both EHT types. Significant changes in beat rate occurred exclusively in EHTWTC11s, where it reduced with 0.3 µM and 3 µM. In conclusion, mavacamten reduced several key contractile parameters with concentration-dependent effects on both EHT types.

### 3.4 Divergent contractile effects of isoproterenol, verapamil and ranolazine on EHTs and monolayers of hPSC-Cardiomyocytes

The contractility of monolayers of iCell Cardiomyocytes2 hPSC-cardiomyocytes was assayed with motion detection, following the same experimental procedures used with EHTs before and after adding varying concentrations of isoproterenol, verapamil, and ranolazine.

**FIGURE 4:**
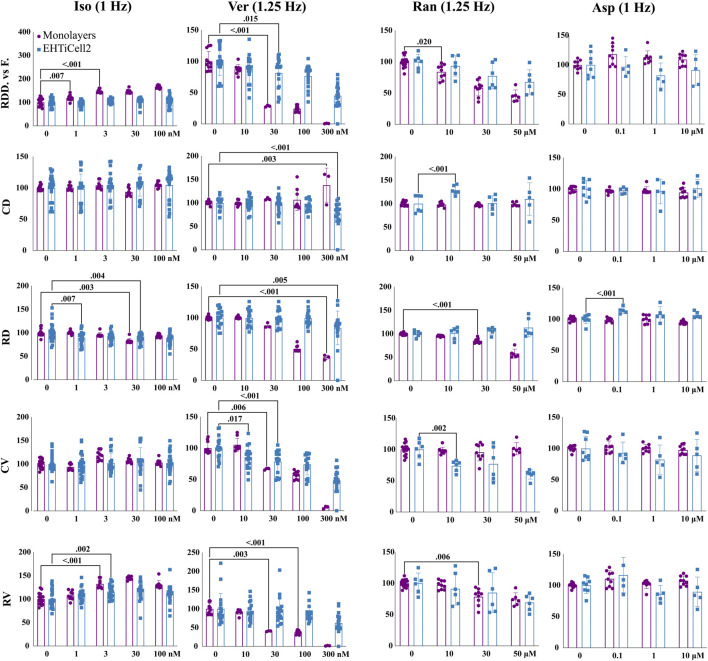
Contractile effects of compounds in EHTs and monolayers of hPSC-cardiomyocytes. Contractile effects of isoproterenol, verapamil, ranolazine, and aspirin in monolayers and EHTs from iCell Cardiomyocytes2 (EHTiCell2) were measured. Images represent relative changes in contractile parameters that were compared between monolayers and EHTiCell2s after incubation for 1 hour in 300 nM of ivabradine prior to adding isoproterenol and aspirin. Data were acquired at 1 and 1.25 Hz pacing rate in modified Tyrode’s solution with 0.6 mM calcium using the EHT measuring system for EHTiCell2s and the SI800 Cell Motion Imaging System for monolayers. Results presented as mean ± SD, *p*-values were determined by two-way ANOVA using Dunnett’s multiple comparison test. Number of experiments (N) and replicates (n) for isoproterenol were: N = 2 and n = (9-17) with monolayers, N = 4 and n = (20-27) with EHTs; for verapamil: N = 2 and n = (3-12) with monolayers, N = 4 and n = 18 with EHTs; for ranolazine: N = 2 and n = (6-15) with monolayers, N = 3 and n = 6 with EHTs; for aspirin: N = 2 and n = 9 with monolayers, N = 2 and n = 8 with EHTs. Iso = isoproterenol, Ver = verapamil, Ran = ranolazine, Asp = aspirin, RDD vs. F. = relaxation deformation distance vs. force, CD = contraction duration, RD = relaxation duration, CV = contraction velocity, RV = relaxation velocity. 3 vials of iCell Cardiomyocytes2 were used in these experiments.

Isoproterenol reduced relaxation duration at 30 nM in monolayers and at 1 and 30 nM in EHTiCell2s at the tested concentrations (Figure 4 and Supplementary Table S19). A dose-dependent increase in deformation distance was detected in monolayers with no effects on EHT force output. Isoproterenol also increased relaxation velocity in monolayers at 3 nM and higher, while contraction duration and contraction velocity remained unaltered in both cell culture formats (Figure 4 and Supplementary Table S19). In summary, effects of isoproterenol at the tested concentrations were detected in monolayers and in EHTs.

Verapamil reduced relaxation and contraction duration in EHTs at 0.3 µM (Figure 4 and Supplementary Table S20). Deformation distance decreased in monolayers at 30 nM and reduction in force in EHTs was detected at 30 nM and higher concentrations. Verapamil at 0.01 µM reduced monolayer contraction velocity and in EHTs at 0.03 µM. A decrease in relaxation velocity was detected in EHTs with the highest tested concentration of 0.3 µM, while a similar effect occurred in monolayers at 0.03 µM. At 0.1 µM, monolayers stopped beating synchronously (Supplementary Videos S6, S7). Overall, verapamil similarly affected the contractility of both tested platforms.

Ranolazine had opposite effects on the relaxation duration of EHTs and monolayers, where contraction duration increased in EHTiCell2s at 10 μM, but not in monolayers at any concentration (Figure 4 and Supplementary Table S21). Relaxation deformation distance (monolayers) and force (EHTs) decreased with most tested concentrations except for 10 µM with EHTs. Relaxation velocity decreased in both platforms, being reduced at 30 µM in monolayers and in EHTs at 50 µM (Figure 4 and Supplementary Table S21). All tested concentrations also reduced contraction velocity in EHTs. In general, relaxation effects caused by ranolazine were detected in both platforms, but were more pronounced at lower concentrations in monolayers, while variations in contraction parameters were mainly significant in EHTs.

Aspirin was used as a control with no effects on contractility (Mannhardt et al., 2016; Mannhardt et al., 2017; Bhagwan et al., 2020; Saleem et al., 2020) (Figure 4 and Supplementary Table S22). Overall, verapamil and ranolazine differently affected each platform in contraction and relaxation durations and disparities in the effective concentrations that caused variations in other parameters were also observed.

### 3.5 Drug-induced variations in EHT contractility correlate to variations in the kinetics of intracellular calcium

**FIGURE 5:**
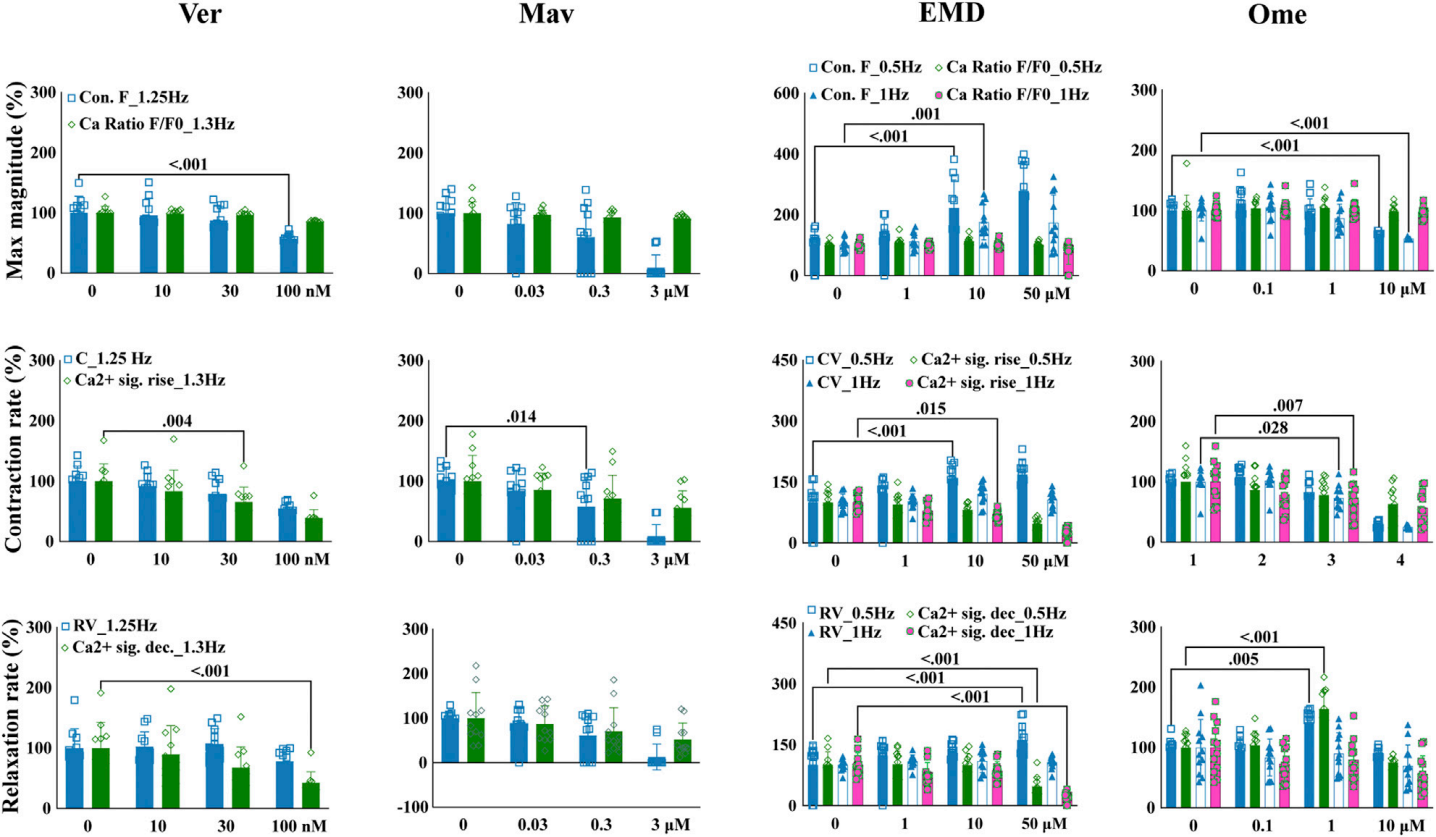
Drug-induced variations in EHT contractility correlate to variations in the kinetics of intracellular calcium transients. Effects of verapamil, mavacamten, EMD57033, and omecamtiv mecarbil on contractility and intracellular calcium transients were measured in EHTs from GCaMPf-expressing hPSC-cardiomyocytes (EHTWTC11) with electrical pacing using the EHT analysis platform and analysis of calcium fluorescence videos. Relative changes are represented in the images where mean responses were recorded at 0.5, 1, 1.25, and 1.3 Hz pacing rates in modified Tyrode’s solution with 0.6 mM calcium. EHTs were pre-incubated for 1 hour in 300 nM ivabradine with EMD57003 and omecamtiv mecarbil. Data represents mean ± SD, *p*-values were determined by two-way ANOVA using Dunnett’s multiple comparison test. Number of experiments (N) and replicates (n) for incubations were: N = 3 and n = 11 using verapamil, N = 3 and n = 7–11 using mavacamten, N = 3 and n = 11 using EMD57003, N = 3 and n = 11 using omecamtiv mecarbil. Ver = verapamil, Mav = mavacamten, EMD = EMD57003, Ome = omecamtiv), Con. F = contraction force, Ca2+ amp = calcium signal amplitude, CV = contraction velocity, Ca2+ sig. rise = calcium signal rise, RV = relaxation velocity, Ca2+ sig. dec = calcium signal decay. 3 vials of WTC11 hPSC-cardiomyocytes were used in these experiments.

To test if the quantified contractile effects could reflect mechanisms underlying the mechanisms of action of compounds that are associated with the kinetics of intracellular calcium, EHT contractile changes were compared with variations in the kinetics of intracellular calcium. EHTWTC11s were dosed with verapamil, mavacamten, EMD57033, and omecamtiv mecarbil, which have distinct mechanisms related to calcium signaling. In concordance with its effects on EHTiCell2s (Figure 3), verapamil reduced contractile force at 100 nM in EHTWTC11s (Figure 5 and Supplementary Table S23). Contraction velocity also decreased at 100 nM and a surrogate effect was detected at a lower concentration of 30 nM as a decrease in the rate of calcium signal rise. Relaxation velocity did not change, while the rate of calcium signal decay decreased at 100 nM. Mavacamten reduced EHT force and contraction velocity at 0.3 µM, while a reduction in the rate of calcium signal rise occurred at 3 µM (Figure 5 and Supplementary Table S24). Both relaxation velocity and rate of calcium signal decay also decreased at 3 μM. EMD57033 increased contractile force at 10 µM and higher, and contraction velocity at 0.5 Hz pacing with concentrations of 10 µM and higher (Figure 5 and Supplementary Table S25). The rate of calcium signal rise decreased at both pacing rates at 50 µM and at 1 Hz with 10 µM. Except for relaxation velocity at 1 Hz, changes in both relaxation velocity and the rate of calcium signal decay were mostly detected at 50 µM under 0.5 Hz pacing rate. Relaxation velocity increased at 0.5 Hz, in contrast with the observed reduction in the rate of calcium signal decay at both rates (Figure 5 and Supplementary Table S25). Omecamtiv mecarbil reduced contractile force at 10 µM with both pacing frequencies (Figure 5 and Supplementary Table S26). Contraction velocity and calcium signal rise decreased for both pacing rates at 10 µM of omecamtiv mecarbil, while this level of reduction was only detected at 1 Hz for both parameters at 1 µM. At this concentration, relaxation velocity increased under both pacing rates, while the rate of calcium signal decay decreased at 10 µM. In conclusion, drug-induced changes in contractility and parameters of variations in calcium transients were generally similar regarding the kinetics of these functional properties, where differences were observed in compounds that are known to have contractile effects mediated by variations in calcium.

### 3.6 Long-term effects including loss of contractile function and recovery due to cardiotoxic compounds in EHTs

**FIGURE 6:**
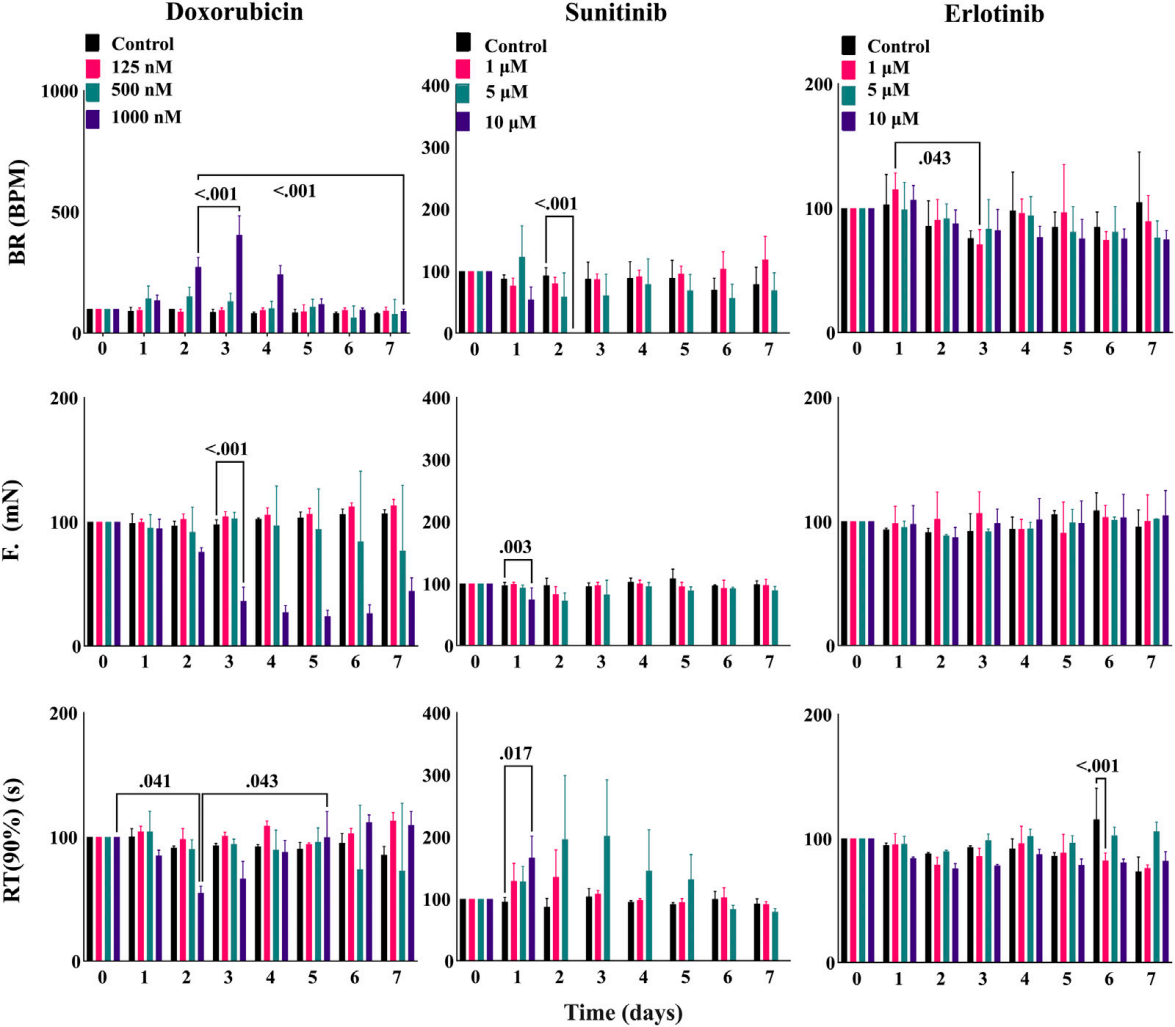
EHT contractile long-term effects of doxorubicin, sunitinib, and erlotinib. EHTs were dosed with doxorubicin, sunitinib, and erlotinib for 48 h (day 0 to day 2) and their contractile output was measured every day until day 7 (5 days after dosing). Images show percent change from the baselines of three contractile parameters: beat rate (BR), contractile force (F), and time to peak from 90% relaxation (RT (90%)). Data presented as mean ± SD, *p*-values were determined by two-way ANOVA using Dunnett’s multiple comparison test. Number of experiments (N) and replicates (n) for each treatment were N = 1 and n = 3 using doxorubicin, N = 1 and n = 3 using sunitinib, N = 1 and n = 3 using erlotinib. 12 vials of iCell Cardiomyocytes2) were used in these experiments.

After ensuring the stability of EHTiCell2s up to several weeks in culture and testing mechanistic effects of drugs, we investigated long-term effects of cardiotoxic drugs within days of exposure (Hansen et al., 2010). EHTiCell2s were incubated for 2 days to varying concentrations of doxorubicin and sunitinib, which are known cardiotoxic drugs, and their effects on contractility during exposure and after washing out were measured. Erlotinib was used as a less cardiotoxic reference control drug (Doherty et al., 2013). Doxorubicin increased the beat rate on day two at 1 µM concentration, which further increased after washout on day three, continued to be higher than baseline values by day four, and started recovering after day five (Figure 6 and Supplementary Table S27). Decreased force occurred at 1 µM after washout between days three and seven (Figure 6 and Supplementary Table S28). Relaxation times did not vary, except on day two in EHTiCell2s dosed with 1 μM, when relaxation time decreased and recovered on day five (Figure 6 and Supplementary Table S29). Sunitinib at 10 µM reduced the beat rate of EHTiCell2s on day two prior to loss of contractile function, in opposition to the increase that was detected with doxorubicin (Figure 6 and Supplementary Table S30). In addition, force decreased on day one and day two with 10 µM of sunitinib. A loss in contractile force was also detected with 5 µM on day two and again on day five when compared with the non-treatment group after washout (Figure 6 and Supplementary Table S30). Relaxation time increased with 10 µM on day one before loss of contractile function and on days two and three with 5 µM. Despite being less cardiotoxic, 1 µM erlotinib decreased beat rate on day three (Figure 6 and Supplementary Table S31) and relaxation time on day six at 1 and 10 µM. Also, relaxation time increased on day seven with 5 µM. However, erlotinib induced less pronounced effects than what was observed with doxorubicin and sunitinib. In summary, the EHTs detected the effects of cardiotoxic drugs exposed for several days and after washout, which allowed for analysis of functional recovery or continued degradation after a toxic insult.

## 4 Discussion

Reproducibility between EHTiCell2 fabrication batches in line with published results was observed in contractile baseline parameters, which included force, beat rate, contraction time, and relaxation time (Jacob et al., 2016; Mannhardt et al., 2017). Similar EHTs generated elsewhere also from iCell cardiomyocytes2 produced 0.18 mN force, 59 beats per minute, 0.17 s long contraction time, and 0.26 s relaxation time between day 12–22 (Breckwoldt et al., 2017). Another study found that atrial trabeculae from a healthy human heart produced a higher force of 6.2 mN along with 0.08 s long contraction time and 0.11 s long relaxation time (Breckwoldt et al., 2017). EHTs after 3 weeks of fabrication in our study produced 0.2 mN of average force at 43 beats per minute, with a contraction time of 0.13 s, and a relaxation time of 0.28 s (Supplementary Figure S1). When investigating the inter-batch reproducibility of EHT baseline function, a maximum of 20%–30% in coefficient of variation were observed in both types of tissues (Supplementary Table S5), which also matched published results with similarly produced EHTs (Mannhardt et al., 2020). Key hallmark properties of mature mammalian myocardium contractility include positive force-frequency relationship and increased force in response to external calcium (Endoh, 2004; Mannhardt et al., 2016). Instead of being positive, a flat force-frequency relationship was observed (Figure 1F), which was in line with similar reported effects of pacing frequency at rates ranging from 0.5 Hz to 2.5 Hz (Jacob et al., 2016). However, positive force-frequency relationships have been demonstrated with EHTs that were electrically trained during differentiation or co-cultured with non-myocyte cells (Ronaldson-Bouchard et al., 2018; Lange et al., 2021). Force increased relative to higher external calcium concentration (Supplementary Figure S2), with an half maximal effective concentration of 0.4 mM, which was close to the 0.6 mM value reported in the literature (Jacob et al., 2016). These results on contractile physiology led to quality performance criteria for EHTs produced with cells differentiated in-house and elsewhere.

Effects of compounds known to affect cardiac contractility are commonly used to characterize cardiac contractility methods (Mannhardt et al., 2016) and a set of these compounds were here tested with EHTs that followed quality control criteria. Overall, drug-induced effects reflected the mechanisms of action of the tested compounds. However, isoproterenol reduced the relaxation duration of EHTs without affecting contractile force (Figure 4 and Supplementary Table S19). This result also partially reflects published EHT results where 100 nM led to an increase in contractile force without altering contraction time, and a decrease in relaxation time (Jacob et al., 2016). The experimental conditions that lead to this result were similar to ours, but different lines of hPSC-cardiomyocytes were used, as well as a 2 Hz pacing frequency, instead of 0.5 Hz and 1 Hz. It was speculated in another study that non-cardiomyocytes in EHTs were required for driving the maturity of the *β*-adrenergic pathway (Feric et al., 2019). Future work should elucidate the roles of such approaches in enhancing the physiology of *β*-adrenergic signaling. Despite potential differences between the origins of used cells, such divergent results could also have derived from distinct experimental conditions in extracellular calcium concentration and electrical stimulation. Ranolazine is known to inhibit the late sodium current and intracellular calcium accumulation (Belardinelli et al., 2006) and, to our knowledge, no other studies have investigated its effects with EHTs. However, contractile effects have been tested with primary cardiomyocytes, where relaxation time was significantly prolonged and sarcomere shortening inhibited by ranolazine (Nguyen et al., 2017). Our data were in line with these results, where ranolazine reduced contractile force, contraction velocity, relaxation velocity and increased contraction time and relaxation time (Figure 4 and Supplementary Table S21). The calcium sensitizer EMD57033 also increased relaxation time, contractile force, and contraction time (Supplementary Figure S8 and Supplementary Tables S6, S7, S9, S25), as previously reported with EHTs and isolated tissue sections (Brixius et al., 2002; Jacob et al., 2016). In addition, effects of omecamtiv mecarbil in primary cardiomyocytes (Abi-Gerges et al., 2020) also aligned with results here presented. However, primary cardiomyocytes showed after-contraction beating incidents, while an opposite outcome was detected in EHTs, where these stopped responding to electrical stimulation (Supplementary Figure S10). A rise in force at 1 µM omecamtiv mecarbil was detected, while decreasing at 10 µM (Figure 2 and Supplementary Table S10), which was similar to what had been published with rodent cardiomyocytes (Nagy et al., 2015). An EHT study with co-cultures of different cell types did not report this bi-phasic effect in force. Contractility has been shown to be reduced in EHTs at 287 nM mavacamten (Feric et al., 2019) and similar reduction in relaxation time, contraction velocity and relaxation velocity was here observed (Figure 3, Supplementary Figure S9, and Supplementary Tables S17, S18, S24). A ryanodine-induced biphasic contractile output (higher contractile force with lower dose of 0.3 µM and lower contractile force at 10 µM) was reported in the literature (Mannhardt et al., 2016) but in a later publication showed no biphasic outcome with different hPSC-cardiomyocyte lines in a concentration range of 1–30 µM (Mannhardt et al., 2020). Instead of a biphasic effect, a dose dependent decrease in contractile force was detected (Supplementary Figure S11). In summary, nine tested compounds in this study mostly matched published results, where differences in experimental conditions seemed to underline the noted divergent data.

Despite observing similar effects of most tested compounds between EHTs and monolayers, monolayers seemed to detect effects of compounds known to decrease contractility, like verapamil, at lower concentrations (Figure 4). Isoproterenol effects were also more pronounced in the monolayers, although a reduction of relaxation duration occurred in both culture platforms (Figure 4 and Supplementary Table S19), and effects on EHTs were more significant at 0.5 Hz than at 1 Hz. In relation to isoproterenol effects, differences between EHTs and monolayers have been reported in calcium current density, catecholamine responses and other biological properties that require a more dedicated focus in future investigations (Mannhardt et al., 2016). The reduction in relaxation time in monolayers and EHTs with verapamil (Figure 4 and Supplementary Table S20) differed from what had been reported in a multi-site study, where verapamil-induced repolarization prolongation was not detected (Blinova et al., 2018). However, reduced contraction and relaxation velocities were observed both here and elsewhere (Blinova et al., 2018). With ranolazine, contractile force decreased in both cell culture platforms (Figure 4 and Supplementary Table S21), while relaxation duration increased in EHTs and decreased in monolayers, which also resembled published monolayer experiments (Blinova et al., 2018), but with responses at a higher concentration. Comparing EHTs with monolayers using the same cells, media and electrical stimulation conditions allowed to avoid any potential effects on function from factors not directly related to physical properties and design of the cell culture platform.

The kinetics of intracellular calcium regulates contractility (Mannhardt et al., 2016). Overall, similar effects of compounds on intracellular calcium and contractile parameters were observed. Saleem and colleagues (Saleem et al., 2020) also compared the contractile output of EHTs with variations in intracellular calcium when dosed with different compounds and observed similar trends in TTP between contractile results and dynamics of calcium transients analysis. The published results of EMD57033 effects differed from what was observed in this study in calcium signal amplitude, TTP and force [32] (Figure 5). This discrepancy could relate to disparities in experimental conditions between studies, such as the concentration of calcium in the test buffer and pacing rate. With omecamtiv mecarbil, and in line with published results (Saleem et al., 2020), no effects on the magnitude of the calcium cycle were observed, despite the observed higher contractile outcome that probably resulted from the myosin-specific effect of this compound (Figure 5 and Supplementary Table S26). More pronounced contractile effects relative to variations in calcium transients were also observed with mavacamten. The concentrations tested with compounds were based on the ranges where effects in contractility were first observed and it is possible that other effects on calcium signal could be detected with different concentration ranges.

EHTs were exposed to cardiotoxic compounds (Figure 6) with the intent of comparing results with published work and investigate the capacity of this platform to test long-term effects of drugs. Overall, the ability to detect acute and subacute effects, and partial recovery after washout were demonstrated with doxorubicin and sunitinib. These results resembled what had been previously reported in other EHT-focused studies (Hansen et al., 2010) and partially differed from data obtained with hPSC-cardiomyocytes in monolayers (Doherty et al., 2013; Pointon et al., 2014; Burridge et al., 2016; Maddah et al., 2020) and primary tissues (Miller et al., 2020). Apoptosis has been shown to occur in monolayers at 500 nM doxorubicin (Miller et al., 2020), which agreed with the effects we observed on EHTs (Figure 6 and Supplementary Tables S27–S29). Considering that doxorubicin cardiotoxicity results from primarily targeting mitochondria (Thomas et al., 2021), contractile dysfunction may be downstream of those effects and therefore less pronounced at lower concentrations or early exposure times. An increase in beat rate without observable toxic effects upon 24 h of exposure to doxorubicin as we observed (Figure 6) had also been detected elsewhere (Burridge et al., 2016), but at 100 nM instead of 1 μM, while contractile dysfunction had been reported with human heart slices also at 100 nM (Miller et al., 2020). To our knowledge, no doxorubicin experiments have been published with EHTs composed of hPSC-cardiomyocytes, but with EHTs composed of murine neonatal cardiomyocytes (Hansen et al., 2010) that observed effects after 96 h of exposure to 1 nM and higher. This disparity from our data could result from inter-species differences, but also from using neonatal cells that may biologically differ from hPSC-cardiomyocytes. Sunitinib exposure to EHTs led to contractile variations at concentration ranges and time points (Figure 6) that were similar to what has been reported in the literature (Doherty et al., 2013; Pointon et al., 2014; Miller et al., 2020). The advantage of using EHTs for characterizing this compound may relate to the possibility of culturing cells for several weeks and eventually having the possibility of characterizing time-dependent effects.

In summary, reproducible contractile function was observed between EHT batches and in line with literature on the same model when following quality control criteria, where baseline function, and response to contractility modulators and cardiotoxic compounds reproduced published data. Additional quality control criteria to ensure EHT reproducibility should also be established for specific contexts of use that may rely on biological mechanisms not considered here. When differentiating cells, quality of hPSCs and of the differentiation efficiency should also be assessed (Morita et al., 2022). The pluripotent state of GCaMPf WTC11 hPSCs in culture was confirmed via immunocytochemical labeling against the markers NANOG and TRA-1-60 (Supplementary Figure S12A). Other markers can be labeled to further assess hPSC pluripotency, as well as other techniques, such as flow cytometry, gene expression and others (Morita et al., 2022). Karyotyping and sequencing of hPSCs, as done elsewhere for WTC11 hPSCs (Johnson et al., 2021), can also be critical to ensure that genetic effects do not affect the outcome of differentiations. In addition to observing differentiated cardiomyocytes expressing GCaMPf (Maddah et al., 2015) as shown in Supplementary Video S8, we labeled alpha-actinin and troponin T in a set of differentiated cells in 2D to assess the efficiency of differentiation (Supplementary Figure S12B). Such markers have been also characterized in the commercially available iCell Cardiomyocytes2 line (Rasmussen et al., 2020) and we used several vials of these cells to account for potential cell batch effects on results. Despite similar baseline contractile function and overall drug responses between EHTiCell2 and EHTWTC11, different results between EHT types could have been caused by distinct differentiation protocols, in addition to potential effects of divergent genetic backgrounds and gene expression. Future work should also investigate effects of variations in cardiomyocyte differentiation properties on the contractility of EHTs. To enhance the physiological relevance of EHT function, several established approaches, including co-culture with other non-cardiac cells (Dunn et al., 2019), electromechanical stimulation (Ronaldson-Bouchard et al., 2018), and variation in the composition of the extracellular matrix (Herron et al., 2016) could also be further explored. Advancements in both biological and engineering components of EHTs will likely assist in the integration of this cellular system in drug development.

## Data Availability

The original contributions presented in the study are included in the article/Supplementary Material, further inquiries can be directed to the corresponding authors.
