# Supplementary material for: Reproducibility of drug-induced effects on the contractility of an engineered heart tissue derived from human pluripotent stem cells
Source: Front Pharmacol. 2023 Jul 4;14:1212092. doi: 10.3389/fphar.2023.1212092 (PMC10352809; doi:10.3389/fphar.2023.1212092)
Supplement: Supplementary file 1 [file DataSheet2.pdf]

Supplementary Material

Reproducibility of drug-induced effects on the contractility of an engineered heart tissue derived from human pluripotent stem cells

Ayesha Arefin*, Melissa Mendoza, Keri Dame, M. Iveth Garcia, David G. Strauss & Alexandre J.S. Ribeiro*

*** Correspondence:**
Ayesha Arefin: rfnayesha@gmail.com
Alexandre J.S. Ribeiro: axribeiro3@gmail.com

Supplementary Tables

Supplementary Tables

S1: EHTs (shaded green) developed stable force.

Percent change (contractile force 0.2 mN as 100%)														
Days	Contractile force (mN)	EHTiCell2-4			EHTiCell2-8			EHTWTC11-3			EHTWTC11-5		
		Mean	Standard deviation	Number of tissue	Mean	Standard deviation	Number of tissue	Mean	Standard deviation	Number of tissue	Mean	Standard deviation	Number of tissue
7	0.2	48	15	6				43	7	14			
14	0.2	83	24	6				58	11	15	31	4	8
22	0.2	90	26	6	26	28	5	65	13	15	46	5	8
29	0.2	94	16	6	35	26	5	82	15	22	48	9	8
34	0.2	101	19	6	40	35	5	85	16	15	68	6	8
42	0.2	88	27	6	35	41	5	64	1	3			
49	0.2	105	19	6				87	19	5			
55	0.2	110	23	6				88	6	4			
68	0.2	109	21	6				79	24	7			
78	0.2	114	23	6				98	18	4			
82	0.2	116	15	6				85	25	11			
90	0.2	116	21	6									

S2: A 0.2 mN contraction force determined as one of the quality control criteria for EHTs.

Week		Contraction force	EHTiCell2aLL (% of 0.2 mN)	Week		Contraction force	EHTWTC11a LL (% of 0.2 mN)
1	0.2	0.09894	49.47	1	0.2	0.09836	49.18
2	0.2	0.1678	83.9	2	0.2	0.1411	70.55
3	0.2	0.2099	104.95	3	0.2	0.1293	64.65
4	0.2	0.2348	117.4	4	0.2	0.1524	76.2
5	0.2	0.2272	113.6	5	0.2	0.1676	83.8
6	0.2	0.1978	98.9	6	0.2	0.181	90.5
7	0.2	0.2069	103.45	7	0.2	0.1703	85.15
8	0.2	0.2358	117.9	8	0.2	0.2078	103.9
9	0.2	0.2227	111.35	9	0.2	0.1949	97.45
10	0.2	0.2219	110.95	10	0.2	0.1903	95.15
11	0.2	0.2232	111.6	11	0.2	0.2169	108.45
12	0.2	0.2396	119.8	12	0.2	0.1782	89.1
13	0.2	0.2193	109.65				

S3: Tissue Fate, and succession rate of EHTiCell2

Tissue fabrication batch number	Number of functional tissues	Vial information (01434)	Culture time	Fate of the tissue	Suspected reason	Succession rate
EHTiCell2 -1	4 out of 4	Vial 1	40 days	Discarded after the experiments		100
EHTiCell2 -2	6 out of 6	Vial 2	56 days	Discarded after the experiments		100
EHTiCell2 -3	2 out of 5	Vial 3	47 days	Discarded after the experiments		40
EHTiCell2 -4	6 out of 6	Vial 4	90 days	Discarded after the experiments		100
EHTiCell2 -5	4 out of 6	Vial 5	83 days	Discarded after the experiments		67
EHTiCell2 -6	1 out of 3	Vial 6	36 days	Discarded after the experiments		33
EHTiCell2 -7	5 out of 8	Vial 7, 8	44 days	Assay development		63
EHTiCell2 -8	0 out of 5	Vial 9	44 days	Force did not develop	Quality of the cells	0
EHTiCell2 -9	6 out of 6	Vial 10	23 days	Assay development		100
EHTiCell2 -10	2 out of 6	Vial 11	21 days	4 out of 6 tissue broke after day 15	Quality of the cells	33
EHTiCell2 -11	7 out of 8	Vial 12, 13	44 days	Assay development		88
EHTiCell2 -12	4 out of 4	Vial 14	42 days	Dox treatment		100
EHTiCell2 -13	8 out of 8	Vial 15,16	60 days	Dox treatment		100
EHTiCell2 -14	12 out of 12	Vial 17,18, 19	41 days	Erlotinib treatment		100
EHTiCell2 -15	15 out of 15	Vial 20, 21, 22	33 days	Dox treatment		100
EHTiCell2 -16	13 out of 14	Vial 23, 24, 25	33 days	Sunitinib treatment		93
EHTiCell2 -17	15 out of 16	Vial 26, 27, 28	33 days	Paclitaxel treatment		94

S4: Tissue Fate, and succession rate of EHTWTC11

Tissue fabrication batch number	Number of functional tissues	Vial informatio n	Culture time	Fate of the tissue	Suspected reason	Succession rate
EHTWTC11 - 1	0 out of 6	1	14 days	Force did not develop	CM differentiation efficiency	0
EHTWTC11 - 2	7 out of 7	2	47 days	Discarded after the experiments		100
EHTWTC11 - 3	12 out of 12	3	83 days	Discarded after the experiments		100
EHTWTC11 - 4	8 out of 8	2	47 days	Discarded after the experiments		100
EHTWTC11 - 5	0 out of 8	2	40 days	Tissue never developed stable force	User error	0

S5: Inter-batch variability of contractile parameters in both types of EHTs

Contractile parameters	EHTiCell2 (15 fabrication batches)	EHTWTC11 (2 fabrication batches)
Contractile force	17.2%	12.11%
Beat rate	19.9%	8.83%
Contraction time	17.2%	27.86%
Relaxation time	17.7%	12.27%
Contraction velocity	18.3%	12.32%
Relaxation velocity	20.8%	13.3%

S6: Effect of EMD57033 on contraction time (TTP) in EHTiCell2 and EHTWTC11

Contraction time	Tissue conditions	Dose	%Change	Significance level	P value
TTP, -10%	EHTiCell2-0.5 Hz	0 µM vs. 50 µM	-508	***	<.001
	EHTWTC11-1 Hz	0 µM vs. 50 µM	-271	*	0.017
TTP, -20%	EHTiCell2-0.5 Hz	0 µM vs. 50 µM	-88	*	0.04
	EHTWTC11-0 .5Hz	0 µM vs. 50 µM	-458	***	<.001
	EHTWTC11-1 Hz	0 µM vs. 50 µM	-236	*	0.01
TTP, -50%	EHTiCell2-0.5 Hz	0 µM vs. 50 µM	-70	*	0.019
	EHTWTC11-0 .5Hz	0 µM vs. 50 µM	-329	***	<.001
	EHTWTC11-1 Hz	0 µM vs. 50 µM	-161	**	0.004
TTP, -80%	EHTiCell2-0.5 Hz	0 µM vs. 50 µM	-48	*	0.044
	EHTWTC11-0 .5Hz	0 µM vs. 10 µM	-119	*	0.031
		0 µM vs. 50 µM	-256	***	<.001
	EHTWTC11-1 Hz	0 µM vs. 50 µM	-136	**	0.002
TTP, -90%	EHTiCell2-0.5 Hz	0 µM vs. 50 µM	-60	**	0.008
	EHTWTC11-0 .5Hz	0 µM vs. 50 µM	-218	***	<.001
	EHTWTC11-1 Hz	0 µM vs. 50 µM	-153	***	<.001

S7: Effect of EMD57033 on relaxation time (RT) in EHTiCell2 and EHTWTC11

Relaxation time	Tissue conditions	Dose	%Change	Significance level	P value
RT, 10%	EHTiCell2-0.5Hz	0 µM vs. 50 µM	-282	***	<.001
	EHTWTC11-0.5Hz	0 µM vs. 10 µM	-446	***	<.001
		0 µM vs. 50 µM	-1332	***	<.001
	EHTiCell2-1Hz	0 µM vs. 50 µM	-276	***	<.001
	EHTWTC11-1Hz	0 µM vs. 10 µM	-432	***	<.001
		0 µM vs. 50 µM	-1376	***	<.001
RT, 20%	EHTiCell2-0.5Hz	0 µM vs. 50 µM	-266	****	<.001
	EHTWTC11-0.5Hz	0 µM vs. 10 µM	-533	****	<.001
		0 µM vs. 50 µM	-1083	****	<.001
	EHTiCell2-1Hz	0 µM vs. 50 µM	-247	****	<.001
	EHTWTC11-1Hz	0 µM vs. 10 µM	-506	****	<.001
		0 µM vs. 50 µM	-1057	****	<.001
RT, 50%	EHTiCell2-0.5Hz	0 µM vs. 10 µM	-91	***	<.001
		0 µM vs. 50 µM	-207	***	<.001
	EHTWTC11-0.5Hz	0 µM vs. 10 µM	-462	***	<.001
		0 µM vs. 50 µM	-593	***	<.001
	EHTiCell2-1Hz	0 µM vs. 50 µM	-186	***	<.001
	EHTWTC11-1Hz	0 µM vs. 10 µM	-443	***	<.001
		0 µM vs. 50 µM	-615	***	<.001
RT, 80%	EHTiCell2-0.5Hz	0 µM vs. 10 µM	-63	**	0.001
		0 µM vs. 50 µM	-158	***	<.001
	EHTWTC11-0.5Hz	0 µM vs. 1 µM	-59	**	0.002
		0 µM vs. 10 µM	-174	***	<.001
		0 µM vs. 50 µM	-243	***	<.001
	EHTiCell2-1Hz	0 µM vs. 50 µM	-132	***	<.001
	EHTWTC11-1Hz	0 µM vs. 10 µM	-177	***	<.001
		0 µM vs. 50 µM	-263	***	<.001

RT, 90%	EHTiCell2-0.5Hz	0 μM vs. 10 μM	-49	*	0.012
		0 μM vs. 50 μM	-149	****	<0.001
	EHTWTC11-0.5Hz	0 μM vs. 1 μM	-50	**	0.003
		0 μM vs. 10 μM	-110	****	<0.001
		0 μM vs. 50 μM	-171	****	<0.001
	EHTiCell2-1Hz	0 μM vs. 50 μM	-112	****	<0.001
	EHTWTC11-1Hz	0 μM vs. 10 μM	-104	****	<0.001
		0 μM vs. 50 μM	-180	****	<0.001

S8: Effect of EMD57033 on relaxation and contraction speed in EHTiCell2 and EHTWTC11

Relaxation velocity	Tissue conditions	Dose	%Change	Significance level	P value
	EHTWTC11-0.5Hz	0 µM vs. 50 µM	-61	**	0.003
	EHTiCell2-1Hz	0 µM vs. 50 µM	29	**	0.002
**Contraction velocity**					
	EHTiCell2-0.5Hz	0 µM vs. 10 µM	-40	***	<.001
	EHTWTC11-0.5Hz	0 µM vs. 10 µM	-58	**	0.003
		0 µM vs. 50 µM	-69	***	<.001
	EHTiCell2-1Hz	0 µM vs. 10 µM	-34	***	<.001

S9: Effect of EMD57033 on contractile force and beat rate in EHTiCell2 and EHTWTC11

Contractile force	Tissue conditions	Dose	%Change	Significance level	P value
	EHTiCell2-0.5Hz	0 µM vs. 10 µM	-35	*	0.017
		0 µM vs. 50 µM	-31	*	0.038
	EHTWTC11-0.5Hz	0 µM vs. 10 µM	-122	*	0.002
		0 µM vs. 50 µM	-178	***	<.001
	EHTiCell2-1Hz	0 µM vs. 10 µM	-42	**	0.005
	EHTWTC11-1Hz	0 µM vs. 10 µM	-75	*	0.018
		0 µM vs. 50 µM	-74	*	0.021
**Beat rate**					
	EHTWTC11-1Hz	0 µM vs. 10 µM	41	***	<.001
		0 µM vs. 50 µM	46	***	<.001

S10: Effect of omecamtiv mecarbil on contractile force and relaxation velocity in EHTiCell2 and EHTWTC11

Contractile force	Tissue conditions	Dose	%Change	Significance level	P value
	EHTiCell2-0.5Hz	0 µM vs. 1 µM	-33	**	0.004
		0 µM vs. 10 µM	53	***	<.001
	EHTiCell2-1Hz	0 µM vs. 10 µM	66	***	<.001
	EHTWTC11-1Hz	0 µM vs. 10 µM	47	*	0.019
Relaxation velocity					
	EHTiCell2-0.5Hz	0 µM vs. 10 µM	36	***	<.001
	EHTWTC11-0.5Hz	0 µM vs. 1 µM	-42	*	0.013
	EHTiCell2-1Hz	0 µM vs. 1 µM	-28	***	<.001
		0 µM vs. 10 µM	54	***	<.001
	EHTWTC11-1Hz	0 µM vs. 1 µM	-64	***	<.001

S11: Effect of omecamtiv mecarbil on relaxation time (RT) in EHTiCell2 and EHTWTC11

Relaxation time	Tissue conditions	Dose	%Change	Significance level	P value
RT, 10%	EHTiCell2-0.5Hz	0 µM vs. 1 µM	-266	***	<.001
		0 µM vs. 10 µM	-592	***	<.001
	EHTWTC11-0.5 Hz	0 µM vs. 1 µM	-582	***	<.001
		0 µM vs. 10 µM	-583	***	<.001
	EHTiCell2-1Hz	0 µM vs. 1 µM	-189	**	0.001
		0 µM vs. 10 µM	-296	***	<.001
	EHTWTC11-1Hz	0 µM vs. 1 µM	-590	***	<.001
RT, 20%	EHTiCell2-0.5Hz	0 µM vs. 1 µM	-211	***	<.001
		0 µM vs. 10 µM	-521	***	<.001
	EHTWTC11-0.5 Hz	0 µM vs. 1 µM	-468	***	<.001
		0 µM vs. 10 µM	-529	***	<.001
	EHTiCell2-1Hz	0 µM vs. 1 µM	-151	***	<.001
		0 µM vs. 10 µM	-252	***	<.001
	EHTWTC11-1Hz	0 µM vs. 1 µM	-433	***	<.001
		0 µM vs. 10 µM	-207	***	<.001
RT, 50%	EHTiCell2-0.5Hz	0 µM vs. 1 µM	-151	***	<.001
		0 µM vs. 10 µM	-384	***	<.001
	EHTWTC11-0.5 Hz	0 µM vs. 0.1 µM	-70	***	<.001
		0 µM vs. 1 µM	-263	***	<.001
		0 µM vs. 10 µM	-324	***	<.001
	EHTiCell2-1Hz	0 µM vs. 1 µM	-103	***	<.001
		0 µM vs. 10 µM	-186	***	<.001
	EHTWTC11-1Hz	0 µM vs. 0.1 µM	-46	*	0.043
		0 µM vs. 1 µM	-202	***	<.001
		0 µM vs. 10 µM	-99	***	<.001
RT, 80%	EHTiCell2-0.5Hz	0 µM vs. 0.1 µM	-25	*	0.048

		0 µM vs. 1 µM	-96	****	<.001
		0 µM vs. 10 µM	-276	****	<.001
	EHTWTC11-0.5 Hz	0 µM vs. 0.1 µM	-44	****	<.001
		0 µM vs. 1 µM	-96	****	<.001
		0 µM vs. 10 µM	-137	****	<.001
	EHTiCell2-1Hz	0 µM vs. 1 µM	-78	****	<.001
		0 µM vs. 10 µM	-131	****	<.001
	EHTWTC11-1Hz	0 µM vs. 1 µM	-60	****	<.001
RT, 90%	EHTiCell2-0.5Hz	0 µM vs. 1 µM	-72	****	<.001
		0 µM vs. 10 µM	-222	****	<.001
	EHTWTC11-0.5 Hz	0 µM vs. 0.1 µM	-23	***	<.001
		0 µM vs. 1 µM	-51	****	<.001
		0 µM vs. 10 µM	-81	****	<.001
	EHTiCell2-1Hz	0 µM vs. 1 µM	-70	****	<.001
		0 µM vs. 10 µM	-105	****	<.001
	EHTWTC11-1Hz	0 µM vs. 1 µM	-35	****	<.001

S12: Effect of omecamtiv mecarbil on contraction times (TTP) in EHTiCell2 and EHTWTC11

Contraction time	Tissue conditions	Dose	%Change	Significance level	P value
**TPP, -10%**	EHTWTC11_0.5 Hz	0 µM vs. 10 µM	-104	***	<.001
	EHTiCell2_1Hz	0 µM vs. 0.1 µM	-42	*	0.031
	EHTWTC11_1Hz	0 µM vs. 10 µM	-71	*	0.01
**TPP, -20%**	EHTWTC11_0.5 Hz	0 µM vs. 10 µM	-161	***	<.001
	EHTWTC11_1Hz	0 µM vs. 10 µM	-190	***	<.001
**TPP, -50%**	EHTiCell2_0.5Hz	0 µM vs. 10 µM	-92	***	<.001
	EHTWTC11_0.5 Hz	0 µM vs. 10 µM	-252	***	<.001
	EHTiCell2_1Hz	0 µM vs. 10 µM	-108	***	<.001
	EHTWTC11_1Hz	0 µM vs. 10 µM	-367	***	<.001
**TPP, -80%**	EHTiCell2_0.5Hz	0 µM vs. 10 µM	-113	***	<.001
	EHTWTC11_0.5 Hz	0 µM vs. 10 µM	-274	***	<.001
	EHTiCell2_1Hz	0 µM vs. 10 µM	-149	***	<.001
	EHTWTC11_1Hz	0 µM vs. 10 µM	-559	***	<.001
**TPP, -90%**	EHTiCell2_0.5Hz	0 µM vs. 10 µM	-113	***	<.001
	EHTWTC11_0.5 Hz	0 µM vs. 10 µM	-264	***	<.001
	EHTiCell2_1Hz	0 µM vs. 10 µM	-138	***	<.001
	EHTWTC11_1Hz	0 µM vs. 10 µM	-539	***	<.001

S13: Effect of omecamtiv mecarbil on contraction velocity (CV) and beat rate in EHTiCell2 and EHTWTC11

Contraction velocity	Tissue conditions	Dose	%Change	Significance level	P value
	EHTiCell2-0.5Hz	0 µM vs. 10 µM	69	***	<.001
	EHTWTC11-0.5Hz	0 µM vs. 10 µM	71	***	<.001
	EHTiCell2-1Hz	0 µM vs. 10 µM	79	***	<.001
	EHTWTC11-1Hz	0 µM vs. 1 µM	27	*	0.029
**Beat rate**					
	EHTiCell2-1Hz	0 µM vs. 10 µM	8	*	0.017
	EHTWTC11-1Hz	0 µM vs. 10 µM	18	***	<.001

S14: Effect of verapamil on contractile force and contraction time (TTP) in EHTiCell2 and EHTWTC11

Contractile force	Tissue conditions	Dose	%Change	Significance level	P value
	EHTiCell2-1.25Hz	0 µM vs. 0.03 µM	19	*	0.011
		0 µM vs. 0.1 µM	24	**	0.001
	EHTWTC11-1.25Hz	0 µM vs. 0.1 µM	43	*	0.01
**Contraction time**					
**TTP, -10%**	EHTiCell2-1.25Hz	0 µM vs. 0.1 µM	17	*	0.029
**TTP, -20%**	EHTiCell2-1.25Hz	0 µM vs. 0.1 µM	17	*	0.014

S15: Effect of verapamil on relaxation time (RT) in EHTiCell2 and EHTWTC11

Relaxation time	Tissue conditions	Dose	%Change	Significance level	P value
**RT, 10%**	EHTWTC11-1.25Hz	0 µM vs. 0.01 µM	-54	*	0.013
		0 µM vs. 0.03 µM	-85	***	<.001
		0 µM vs. 0.1 µM	-79	***	<.001
**RT, 20%**	EHTWTC11-1.25Hz	0 µM vs. 0.01 µM	-51	**	0.007
		0 µM vs. 0.03 µM	-71	***	<.001
		0 µM vs. 0.1 µM	-65	***	<.001
**RT, 50%**	EHTWTC11-1.25Hz	0 µM vs. 0.01 µM	-35	**	0.006
		0 µM vs. 0.03 µM	-36	**	0.004
		0 µM vs. 0.1 µM	-27	*	0.042
**RT, 80%**	EHTWTC11-1.25Hz	0 µM vs. 0.01 µM	-15	*	0.04
**RT, 90%**	EHTWTC11-1.25Hz	0 µM vs. 0.01 µM	-18	*	0.014

S16: Effect of verapamil on contraction speed in EHTiCell2 and EHTWTC11

Contraction velocity	Tissue conditions	Dose	%Change	Significance level	P value
	EHTiCell2-1.25Hz	0 µM vs. 0.01 µM	15	*	0.018
		0 µM vs. 0.03 µM	22	***	<.001
		0 µM vs. 0.1 µM	26	***	<.001
	EHTWTC11-1.25Hz	0 µM vs. 0.1 µM	45	***	<.001

S17: Effect of mavacamten on force, contraction times (TTP) and relaxation time (RT) in EHTiCell2 and EHTWTC11

Contractile force	Tissue conditions	Dose	%Change	Significance level	P value
	EHTiCell2-1.25Hz	0 µM vs. 3 µM	40	****	<.001
	EHTWTC11-1.25Hz	0 µM vs. 3 µM	85	***	<.001
**Contraction time**					
**TPP, -10%**	EHTiCell2-1.25Hz	0 µM vs. 0.3 µM	11	*	0.562
		0 µM vs. 3 µM	28	***	0.02
	EHTWTC11-1.25Hz	0 µM vs. 3 µM	75	**	0.009
**TPP, -20%**	EHTWTC11-1.25Hz	0 µM vs. 3 µM	77	**	0.004
**TPP-50%**	EHTiCell2-1.25Hz	0 µM vs. 3 µM	24	*	0.016
	EHTWTC11-1.25Hz	0 µM vs. 3 µM	72	**	0.002
**TPP, -80%**	EHTiCell2-1.25Hz	0 µM vs. 3 µM	22	*	0.022
	EHTWTC11-1.25Hz	0 µM vs. 3 µM	70	**	0.001
**TPP, -90%**	EHTiCell2-1.25Hz	0 µM vs. 3 µM	20	*	0.035
	EHTWTC11-1.25Hz	0 µM vs. 3 µM	71	***	<.001
**Relaxation time**					
**RT, 10%**	EHTWTC11-1.25Hz	0 µM vs. 3 µM	72	*	0.02
**RT, 20%**	EHTiCell2-1.25Hz	0 µM vs. 3 µM	24	*	0.016
	EHTWTC11-1.25Hz	0 µM vs. 3 µM	73	**	0.002
**RT, 50%**	EHTWTC11-1.25Hz	0 µM vs. 3 µM	73	***	<.001
**RT, 80%**	EHTWTC11-1.25Hz	0 µM vs. 0.3 µM	31	*	0.037
		0 µM vs. 3 µM	69	***	<.001
**RT, 90%**	EHTWTC11-1.25Hz	0 µM vs. 0.3 µM	32	*	0.023

		0 μM vs. 3 μM	71	***	<.001

S18: Effect of mavacamten on beat rate, contraction (CV) and relaxation velocity (RV) in EHTiCell2 and EHTWTC11

Beat rate	Tissue conditions	Dose	%Change	Significance level	P value
	EHTWTC11-1.25Hz	0 µM vs. 0.3 µM	34	**	0.026
		0 µM vs. 3 µM	69	****	<.001
**Contraction velocity**					
	EHTiCell2-1.25Hz	0 µM vs. 3 µM	39	***	<.001
	EHTWTC11-1.25Hz	0 µM vs. 0.3 µM	42	*	0.015
		0 µM vs. 3 µM	86	***	<.001
**Relaxation velocity**					
	EHTiCell2-1.25Hz	0 µM vs. 3 µM	37	***	<.001
	EHTWTC11-1.25Hz	0 µM vs. 3 µM	80	**	0.006

S19: Effect of Isoproterenol on contraction in monolayer and EHTiCell2

Isoproterenol	Monolayer 1.25 Hz				EHTiCell2 1.25 Hz			
**Parameters**	Dose (µM)	Change (%)	Significance	P value	Change (%)	Significance	P value	Takeaway
**Contraction deformation distance**	0 vs. 0.003	-20	**	.003				No change
**Relaxation deformation distance**	0 vs. 0.001<br>0 vs. 0.003<br>0 vs. 0.03<br>0 vs. 0.1	-19<br>-48<br>-46<br>-66	**<br>***<br>***<br>***	.007<br><.001<br><.001<br><.001				Increased in monolayer
**Contraction duration**								No change
**Relaxation duration**	0 vs. 0.001<br>0 vs. 0.03<br>0 vs. 0.1	17	**	.003	11<br>12<br>13	**<br>**<br>***	.007<br>.004<br><.001	Decreased in EHT and monolayer
**Contraction velocity**								No change
**Relaxation velocity**	0 vs. 0.003<br>0 vs. 0.03<br>0 vs. 0.1	-32<br>-44<br>-30	***<br>***<br>***	<.001<br><.001<br><.001	-16<br>-17<br>-11	**<br>***<br>*	.007<br>.004<br><.001	Increased in EHT and monolayer

S20: Effect of Verapamil contraction in monolayer and EHTiCell2

Verapamil	Monolayer-1.25 Hz				EHTiCell2-1.25 Hz				
**Parameters**	**Dose (µM)**	**Change (%)**	**Significance**	**P value**	**Dose (µM)**	**Change (%)**	**Significance**	**P value**	**Takeaway**
**Contraction deformation distance**	0 vs. 0.03	35	*	0.024	0 vs. 0.03<br>0 vs. 0.1<br>0 vs. 0.3	19<br>24<br>55	*<br>**<br>***	0.017<br>0.001<br><.001	Decreased in both platform
**Relaxation deformation distance**	0 vs. 0.03	72	***	<.001	0 vs. 0.03<br>0 vs. 0.1<br>0 vs. 0.3	19<br>24<br>55	*<br>**<br>***	0.016<br>0.001<br><.001	Decreased in both platform
**Contraction duration**					0 vs. 0.3	23	***	<.001	Decreased in EHT, monolayer not reliable
**Relaxation duration**					0 vs. 0.3	16	**	0.003	Decreased in both platform
**Contraction velocity**	0 vs. 0.03	33	**	0.006	0 vs. 0.01<br>0 vs. 0.03<br>0 vs. 0.1	15<br>22<br>26	*<br>***<br>***	0.017<br><.001<br><.001	Decreased in both platform
**Relaxation velocity**	0 vs. 0.03	59	**	0.004	0 vs. 0.3	37	***	<.001	Decreased in both platform
**Beat rate**	0 vs. 0.1	-39	***	<.001					Deceased in monolayers

S21: Effect of Ranolazine on contraction kinetics in monolayer and EHTiCell2

Ranolazine	Monolayer-1.25 Hz	EHTiCell2-1.25 Hz	Takeaway

**Parameters**	**Dose (µM)**	**Change (%)**	**Significance**	**P value**	**Dose (µM)**	**Change (%)**	**Significance**	**P value**	
**Contraction deformation distance**					0 vs. 30<br>0 vs. 50	23<br>32	*<br>***	0.016<br><.001	Decreased in EHT
**Relaxation deformation distance**	0 vs. 10<br>0 vs. 30<br>0 vs. 50	16<br>42<br>55	*<br>***<br>***	0.019<br><.001<br><.001	0 vs. 30<br>0 vs. 50	23<br>32	*<br>***	0.014<br><.001	Decreased in both
**Contraction duration**					0 vs. 10	-29	***	<.001	Increased in EHT
**Relaxation duration**	0 vs. 30<br>0 vs. 50	15<br>42	***<br>****	<.001<br><.001	0 vs. 50	-13	*	0.025	Decreased in monolayer but increased in EHT
**Contraction velocity**					0 vs. 10<br>0 vs. 30<br>0 vs. 50	26<br>23<br>40	**<br>**<br>***	0.002<br>0.008<br><.001	Decreased in EHT
**Relaxation velocity**	0 vs. 30<br>0 vs. 50	21<br>26	**<br>**	0.005<br>0.004	0 vs. 50	31	**	0.003	Decreased in both
**Beat rate**	0 vs. 50	25	***	<.001					Decreased in monolayer

S22: Effect of Aspirin on contraction kinetics in monolayer and EHTiCell2

**Aspirin**	**Monolayer 1 Hz**				**EHTiCell2 1 Hz**				**Takeaway**
**Parameters**	Dose (µM)	Change (%)	Significance	P value	Dose (µM)	Change (%)	Significance	P value	

**Contraction deformation distance**						
**Relaxation deformation distance**						
**Contraction duration**						
**Relaxation duration**		0 vs. 0.1	-14	***	<.001	Increased in EHT
**Contraction velocity**						
**Relaxation velocity**						

S23: Verapamil induced variability in EHT contractility and variations in kinetics of calcium transients

Verapamil	Contractility					Calcium transients				
**Parameters**	**Freq (Hz)**	**Dose (nM)**	**Change (%)**	**Significance**	**P value**	**Freq (Hz)**	**Dose (nM)**	**Change (%)**	**Significance**	**P value**
**Contractile force vs magnitude of the normalized peak intensity of calcium signals**	1.25	0 vs. 100	43	***	<.001					
**Contraction velocity vs rate of calcium signal rise**	1.25	0 vs. 100	45	***	<.001	1.3	0 vs. 30<br>0 vs. 100	34<br>61	**<br>***	0.004<br><.001
**Relaxation velocity vs rate of calcium signal decay**						1.3	0 vs. 100	57	***	<.001

S24: Mavacamten induced variability in EHT contractility and variations in kinetics of calcium transients

Mavacamten	Contractility					Calcium transients				
**Parameters**	**Freq (Hz)**	**Dose (µM)**	**Change (%)**	**Significance**	**P value**	**Freq (Hz)**	**Dose (µM)**	**Change (%)**	**Significance**	**P value**
**Contractile force vs magnitude of the normalized peak intensity of calcium signals**	1.25<br>1.25	0 vs. 0.3<br>0 vs. 3	40<br>85	**<br>***	0.003<br><.001					
**Contraction velocity vs rate of calcium signal rise**	1.25<br>1.25	0 vs. 0.3<br>0 vs. 3	42<br>86	*<br>***	0.018<br><.001	1.3	0 vs. 3	44	*	0.011
**Relaxation velocity vs rate of calcium signal decay**	1.25	0 vs. 3	80	***	<.001	1.3	0 vs. 3	48	*	0.029

S25: EMD57033 induced variability in EHT contractility and variations in kinetics of calcium transients

EMD57033	Contractility					Calcium transients				
**Parameters**	**Freq (Hz)**	**Dose (µM)**	**Change (%)**	**Significance**	**P value**	**Freq (Hz)**	**Dose (µM)**	**Change (%)**	**Significance**	**P value**
**Contractile force vs magnitude of the normalized peak intensity of calcium signals**	0.5	0 vs. 10	-122	***	<.001					
		0 vs. 50	-178	***	<.001					
	1	0 vs. 10	-75	**	0.001					
		0 vs. 50	-74	**	0.002					
**Contraction velocity vs rate of calcium signal rise**	0.5	0 vs. 10	-59	***	<.001	0.5	0 vs. 50	53	***	<.001
		0 vs. 50	-69	***	<.001	1	0 vs. 10	33	*	0.015
							0 vs. 50	75	***	<.001
**Relaxation velocity vs rate of calcium signal decay**	0.5	0 vs. 10	-35	*	0.015	0.5	0 vs. 50	53	***	<.001
	0.5	0 vs. 50	-61	***	<.001	1	0 vs. 50	79	***	<.001

S26: Omecamtiv mecarbil induced variability in EHT contractility and variations in kinetics of calcium transients

Omecamtiv mecarbil	Contractility					Calcium transients				
Parameters	Freq (Hz)	Dose (μM)	Change (%)	Significance	P value	Freq (Hz)	Dose (μM)	Change (%)	Significance	P value
Contractile force vs magnitude of the normalized peak intensity of calcium signals	0.5	0 vs. 10	38	***	<.001					
	1	0 vs. 10	47	***	<.001					
Contraction velocity vs rate of calcium signal rise	0.5	0 vs. 10	71	***	<.001	0.5	0 vs. 10	37	***	<.001
	1	0 vs. 1<br>0 vs. 10	27<br>75	*<br>***	0.028<br><.001	1	0 vs. 1<br>0 vs. 10	30<br>43	**<br>***	0.007<br><.001
Relaxation velocity vs rate of calcium signal decay	0.5	0 vs. 1	-42	**	0.005	0.5	0 vs. 10	30	*	0.039
	1	0 vs. 1	-64	***	<.001	1	0 vs. 10	44	**	0.001

S27: Effect of doxorubicin on beat rate

Comparison between doses from day 0 to 7				
**Dose (nM)**	**Treatment days**	**%change**	**Summary**	**Adjusted P Value**
Control vs. 1000	Day 2	-172	***	<.001
Control vs. 1000	Day 3	-318	***	<.001
Control vs. 1000	Day 4	-160	***	<.001
**Comparison between days for treatment group**				
500 nM				
	1 vs. 6	79	**	0.008
	2 vs. 6	87	**	0.002
	2 vs. 7	73	*	0.017
	3 vs. 6	66	*	0.043
1000 nM	0 vs. 2	-172	***	<.001
	0 vs. 3	-305	***	<.001
	0 vs. 4	-142	***	<.001
	1 vs. 2	-137	***	<.001
	1 vs. 3	-270	***	<.001
	1 vs. 4	-107	***	<.001
	2 vs. 3	-133	***	<.001
	2 vs. 5	152	***	<.001
	2 vs. 6	177	***	<.001
	2 vs. 7	182	***	<.001
	3 vs. 4	163	***	<.001
	3 vs. 5	285	***	<.001
	3 vs. 6	310	***	<.001
	3 vs. 7	314	***	<.001
	4 vs. 5	122	***	<.001
	4 vs. 6	147	***	<.001
	4 vs. 7	152	***	<.001

Supplementary Material

S28: Effect of doxorubicin on force

Comparison between doses from day 0 to 7				
**Dose (nM)**	**Treatment days**	**% change**	**Summary**	**Adjusted P Value**
**Control vs. 1000**	Day 3	62	**	0.001
**125 vs. 1000**		68	***	<.001
**500 vs. 1000**		66	***	<.001
**Control vs. 1000**	Day 4	75	***	<.001
**125 vs. 1000**		78	***	<.001
**500 vs. 1000**		70	***	<.001
**Control vs. 1000**	Day 5	80	***	<.001
**125 vs. 1000**		82	***	<.001
**500 vs. 1000**		70	***	<.001
**Control vs. 1000**	Day 6	80	***	<.001
**125 vs. 1000**		86	***	<.001
**500 vs. 1000**		58	**	0.001
**Control vs. 1000**	Day 7	62	***	<.001
**125 vs. 1000**		69	***	<.001
Comparison between days for treatment group 1000 nM				
**1000 nM**	0 vs. 3	64	**	0.003
	0 vs. 4	73	***	<.001
	0 vs. 5	76	***	<.001
	0 vs. 6	74	***	<.001
	0 vs. 7	56	*	0.016
	1 vs. 3	59	**	0.009
	1 vs. 4	68	**	0.001
	1 vs. 5	71	***	<.001
	1 vs. 6	68	**	0.001
	1 vs. 7	51	*	0.04
	2 vs. 5	52	*	0.032

	2 vs. 6	50	*	0.048

S29: Effect of doxorubicin on relaxation time

Comparison between doses from day 0 to 7				
**Dose (nM)**	**Treatment days**	**%change**	**Summary**	**Adjusted P Value**
**Control vs. 1000 nM**	Day 2	36	*	0.032
**Comparison between days for treatment group**				
**1000 nM**	0 vs. 2	45	*	0.041
	2 vs. 5	-45	*	0.043
	2 vs. 6	-57	**	0.003
	2 vs. 7	-55	**	0.006
	3 vs. 6	-46	*	0.038

S30: Effect of sunitinib in EHT

Beat rate				
**Comparison with control**				
**Dose (µM)**	**Treatment days**	**%change**	**Summary**	**Adjusted P Value**
Control vs. 10 µM	Day 2	93	***	<.001
**Comparison between individual treatment groups**				
5 µM	Day 1 vs. Day 2	64	*	0.014
	Day 1 vs. Day 3	62	*	0.02
	Day 1 vs. Day 6	66	**	0.01
10 µM	Day 0 vs. Day 2	100	***	<.001
**Force**				
**Comparison with control**				
Control vs. 10 µM	Day 1	23	**	0.003
Control vs. 5 µM	Day 2	25	**	0.001
Control vs. 10 µM	Day 2	97	***	<.001
Control vs. 5 µM	Day 5	20	*	0.012
**Comparison between individual treatment groups**				
5 µM	Day 0 vs. Day 2	27	**	0.003
	Day 2 vs. Day 4	-23	*	0.025
10 µM	Day 0 vs. Day 1	26	**	0.006
	Day 0 vs. Day 2	100	***	<.001
	Day 1 vs. Day 2	74	***	<.001
**Relaxation time**				
**Comparison with control**				
Control vs. 10 µM	Day 1	-71	*	0.017
Control vs. 5 µM	Day 2	-109	***	<.001
Control vs. 5 µM	Day 3	-98	***	<.001
**Comparison between individual treatment groups**				
5 µM	Day 0 vs. Day 2	-96	**	0.007
	Day 0 vs. Day 3	-102	**	0.003
	Day 2 vs. Day 6	113	***	<.001

	Day 2 vs. Day 7	117	***	<.001
	Day 3 vs. Day 6	119	***	<.001
	Day 3 vs. Day 7	123	***	<.001
10 μM	Day 0 vs. Day 2	100	**	0.004

S31: Effect of erlotinib in EHTs

Beat rate				
**Comparison between days for all the treatment group**				
**Dose (µM)**	**Days**	**%change**	**Summary**	**Adjusted P Value**
1 µM	1 vs. 3	44	*	0.043
**Relaxation time**				
**Comparison between doses from day 0 to 7**				
**Treatment days**	**Dose (µM)**	**%change**	**Summary**	**Adjusted P Value**
Day 6	Control vs. 1 µM	33	***	<.001
	Control vs. 10 µM	35	***	<.001
Day 7	Control vs. 5 µM	-32	***	<.001
**Comparison between days for all the treatment group**				
**Dose (µM)**	**Days**	**%change**	**Summary**	**Adjusted P Value**
Control	0 vs. 7	27	*	0.013
	2 vs. 6	-28	**	0.009
	4 vs. 6	-23	*	0.043
	5 vs. 6	-29	**	0.004
	6 vs. 7	42	***	<.001
1 µM	0 vs. 2	21	*	0.017
	0 vs. 7	24	**	0.005
	1 vs. 7	19	*	0.044
	4 vs. 7	20	*	0.031
10 µM	0 vs. 2	24	**	0.004
	0 vs. 3	22	*	0.012
	0 vs. 5	22	*	0.014
	0 vs. 6	19	*	0.039
