# Supplementary material for: Reproducibility of drug-induced effects on the contractility of an engineered heart tissue derived from human pluripotent stem cells
Source: Front Pharmacol. 2023 Jul 4;14:1212092. doi: 10.3389/fphar.2023.1212092 (PMC10352809; doi:10.3389/fphar.2023.1212092)
Supplement: Supplementary file 4 [file DataSheet4.pdf]

SUPPLEMENTARY VIDEOS

Accompanying the article entitled:

Reproducibility of drug-induced effects on the contractility of an engineered heart tissue derived from human pluripotent stem cells

Ayesha Arefin[1,2*], Melissa Mendoza[1], Keri Dame[1], M. Iveth Garcia[1], David G. Strauss[1,3] & Alexandre J.S. Ribeiro[1*]

[1]U.S. Food and Drug Administration, Center for Drug Evaluation and Research, Office of Translational Sciences, Office of Clinical Pharmacology, Division of Applied Regulatory Science, Silver Spring, MD, USA
[2]U.S. Food and Drug Administration, National Center for Toxicological Research, Division of Systems Biology, Jefferson, AR, USA
[3]U.S. Food and Drug Administration, Center for Drug Evaluation and Research, Office of Clinical Pharmacology, Silver Spring, MD, USA

*Corresponding authors:
Ayesha Arefin: rfnayesha@gmail.com
Alexandre J.S. Ribeiro: aribeirostanford@gmail.com

Supplementary Video S1: Related to Figure 1.

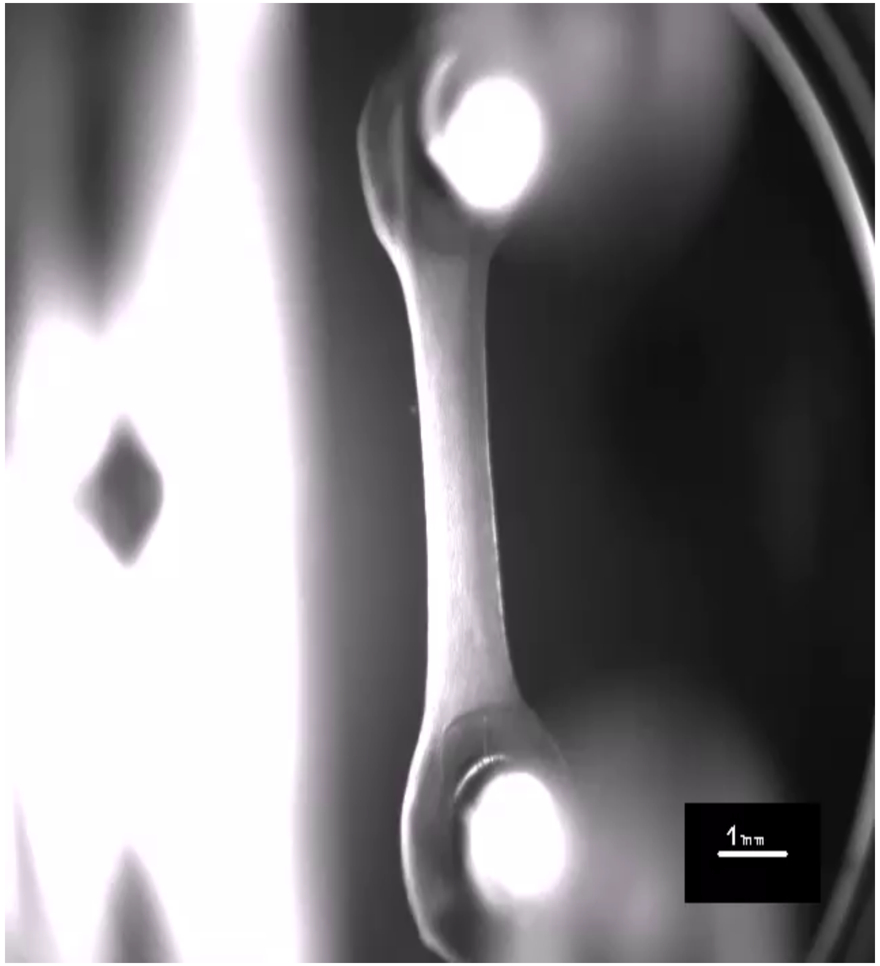

Supplementary Video S1: Morphology of EHTiCell2. Engineered heart tissue was generated from iCell cardiomyocyte2 and was maintained in EHT medium. The video was captured using EHT measuring system.

Supplementary Video S2: Related to Figure 1.

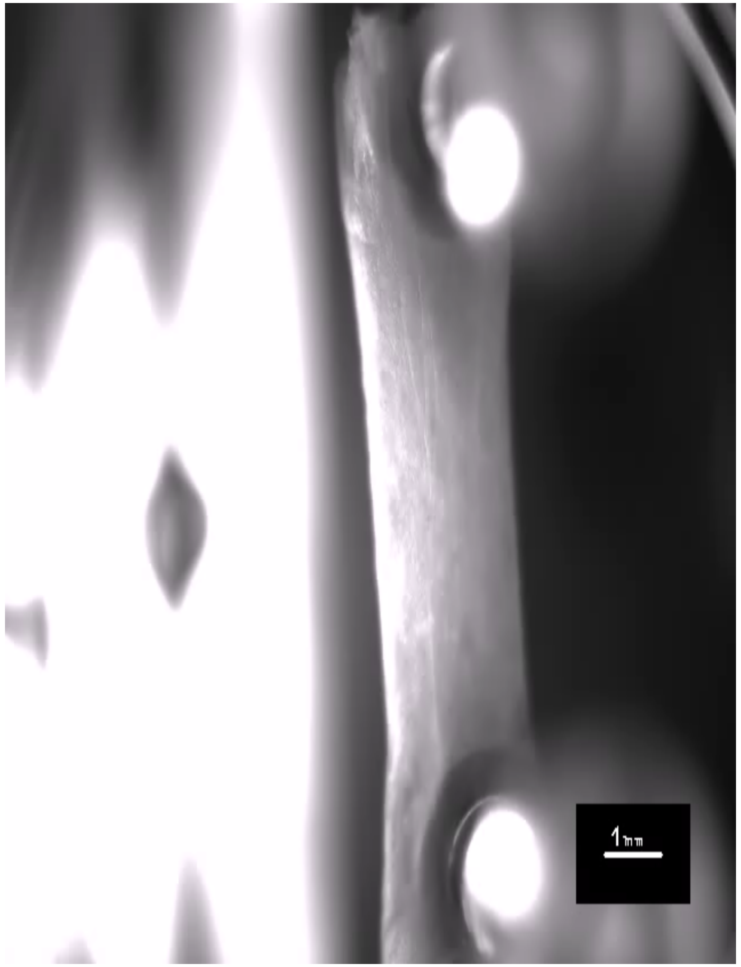

Supplementary Video S2: **Asynchronous beating of EHT on Day 1.** Engineered heart tissue was generated from iCell cardiomyocyte2 was maintained in EHT medium. The video was captured using EHT measuring system at 24 hours after the tissue generation.

Supplementary Video S3: Related to Figure 1.

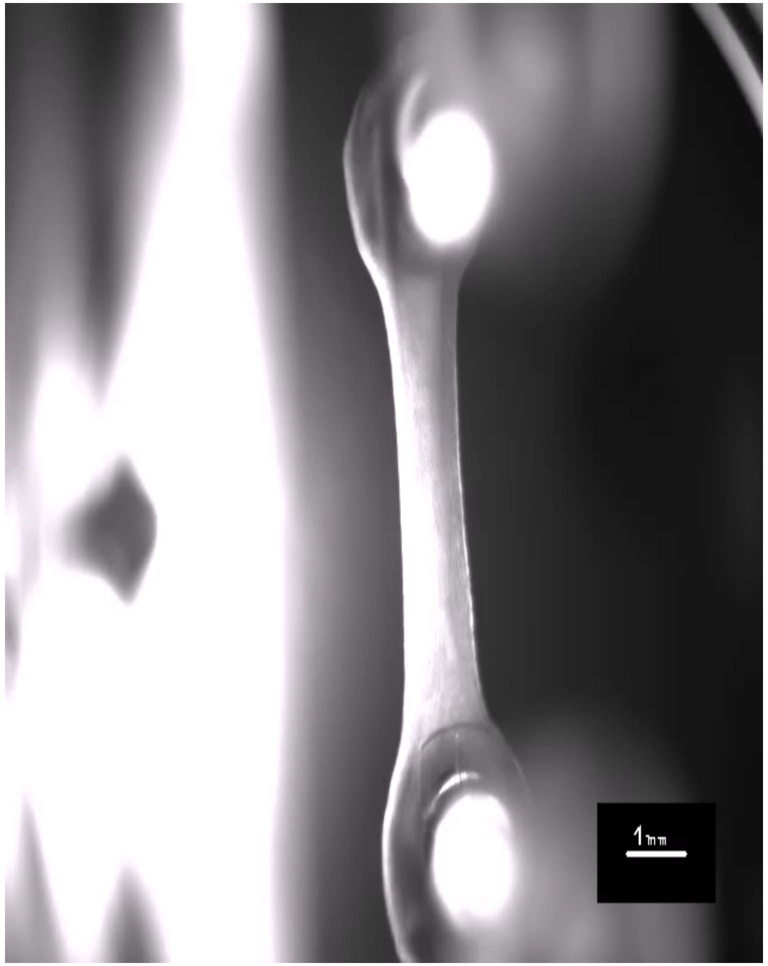

Supplementary Video S3: Synchronous beating of EHT on Day 14. Engineered heart tissue was generated from iCell cardiomyocyte2 and was maintained in EHT medium. The video was captured using EHT measuring system at day 14 after the tissue generation.

Supplementary Video S4: Related to Figure 1.

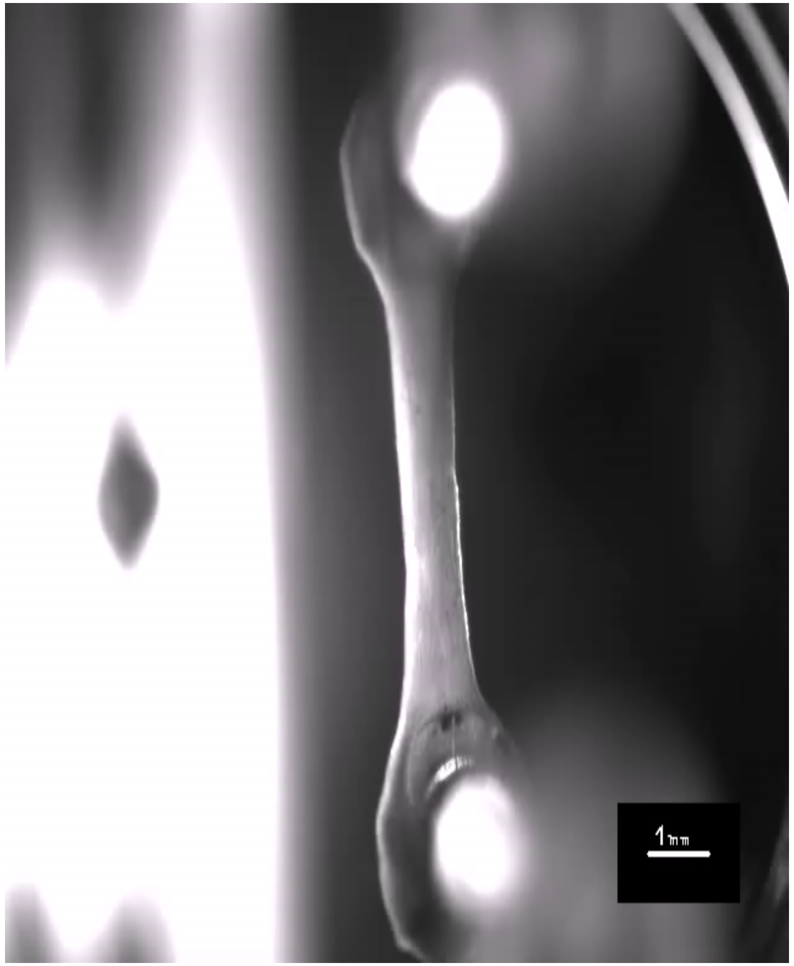

Supplementary Video S4: Synchronous beating of EHT on Day 90. Engineered heart tissue was generated from iCell cardiomyocyte2 and was maintained in EHT medium. The video was captured using EHT measuring system at day 90 after the tissue generation.

Supplementary Video S5: Related to Figure 1.

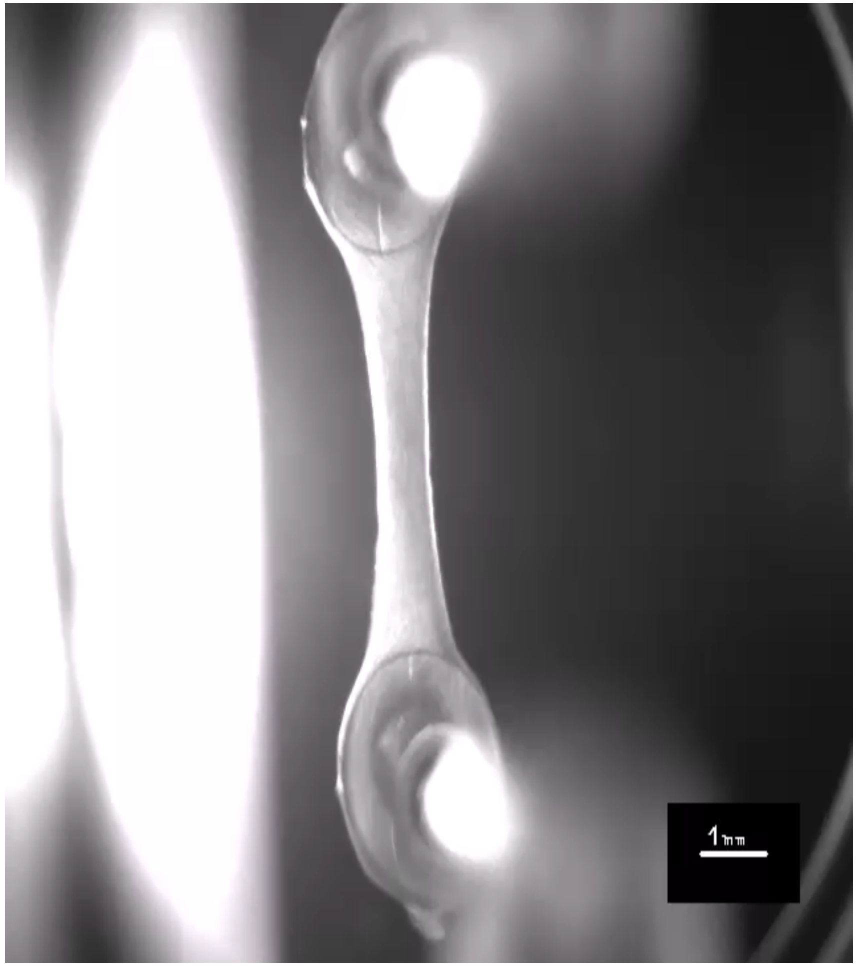

Supplementary Video S5: Morphology of EHTWTC11. Engineered heart tissue was generated from WTC11-GCaMPf-hPSC-cardiomyocytes and was maintained in EHT medium. The video was captured using EHT measuring system.

Supplementary Video S6: Related to Figure 4.

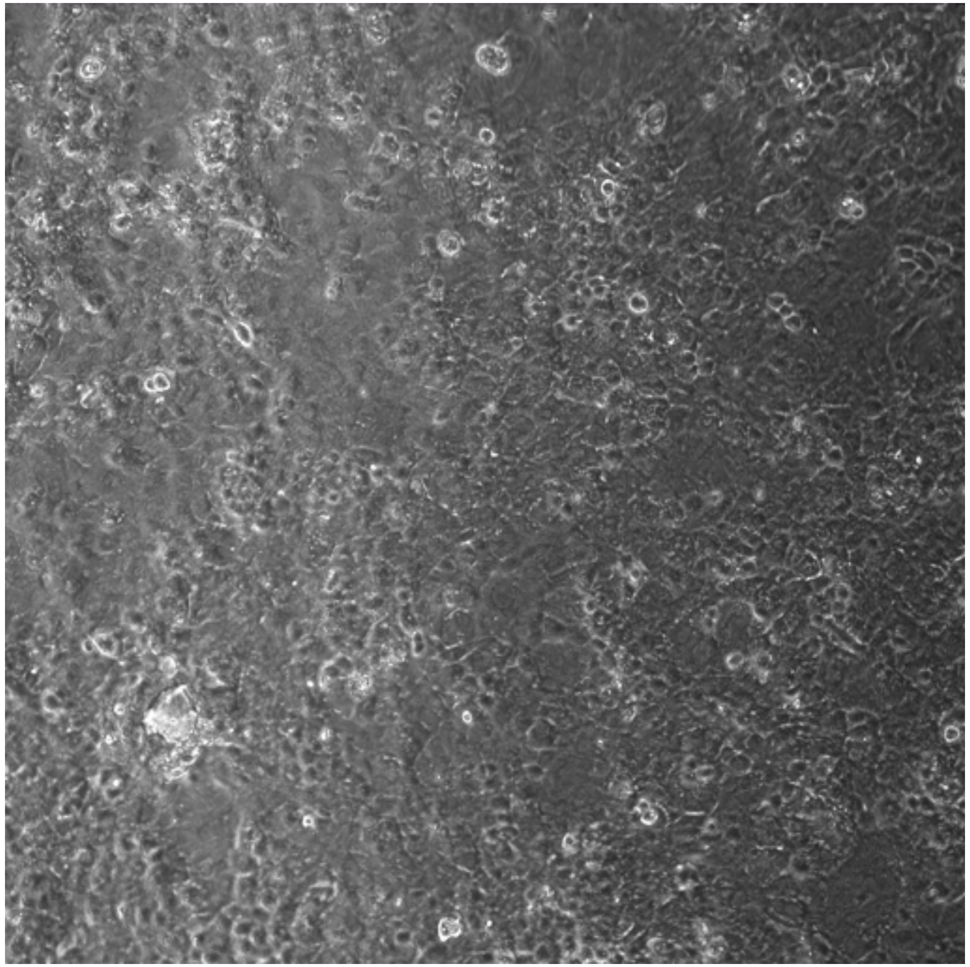

Supplementary Video S6: Contraction activity of Monolayer at 0 nM verapamil exposure. iCell cardiomyocyte2s cultured on 24 well plate were dosed with various concentrations of verapamil under 1.25 Hz pacing rate. The video represents the contractile activity of the control well of iCell cardiomyocyte monolayer in Tyrode's solution with 0.6 mM calcium captured with SI8000 Cell Motion Imaging System.

Supplemental Video S7: Related to Figure 4.

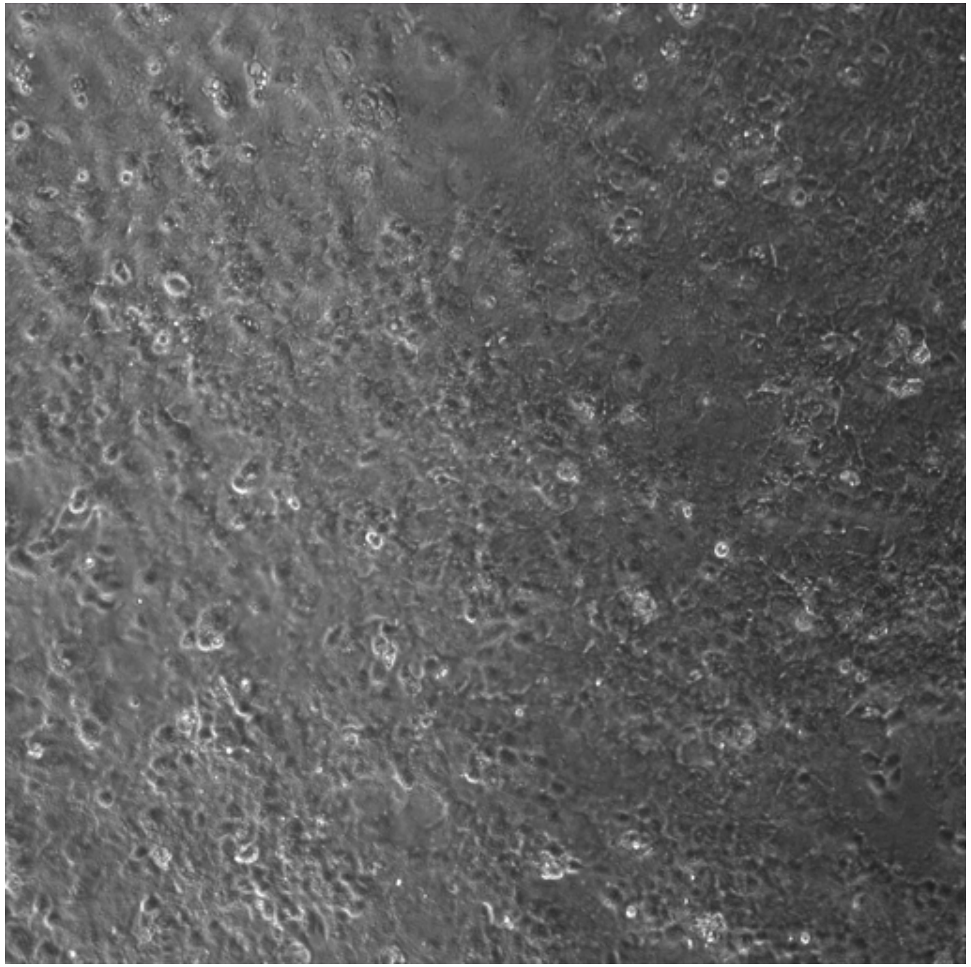

Supplemental Video S7: Contraction activity of Monolayer at 100 nM verapamil exposure. iCell cardiomyocytes cultured on 24 well plate were dosed with various concentrations of verapamil under 1.25 Hz pacing rate. The video represents the contractile activity of iCell cardiomyocyte monolayer dosed with 100 nM of verapamil in Tyrode's solution with 0.6 mM calcium captured with SI8000 Cell Motion Imaging System.

Supplementary Video S8

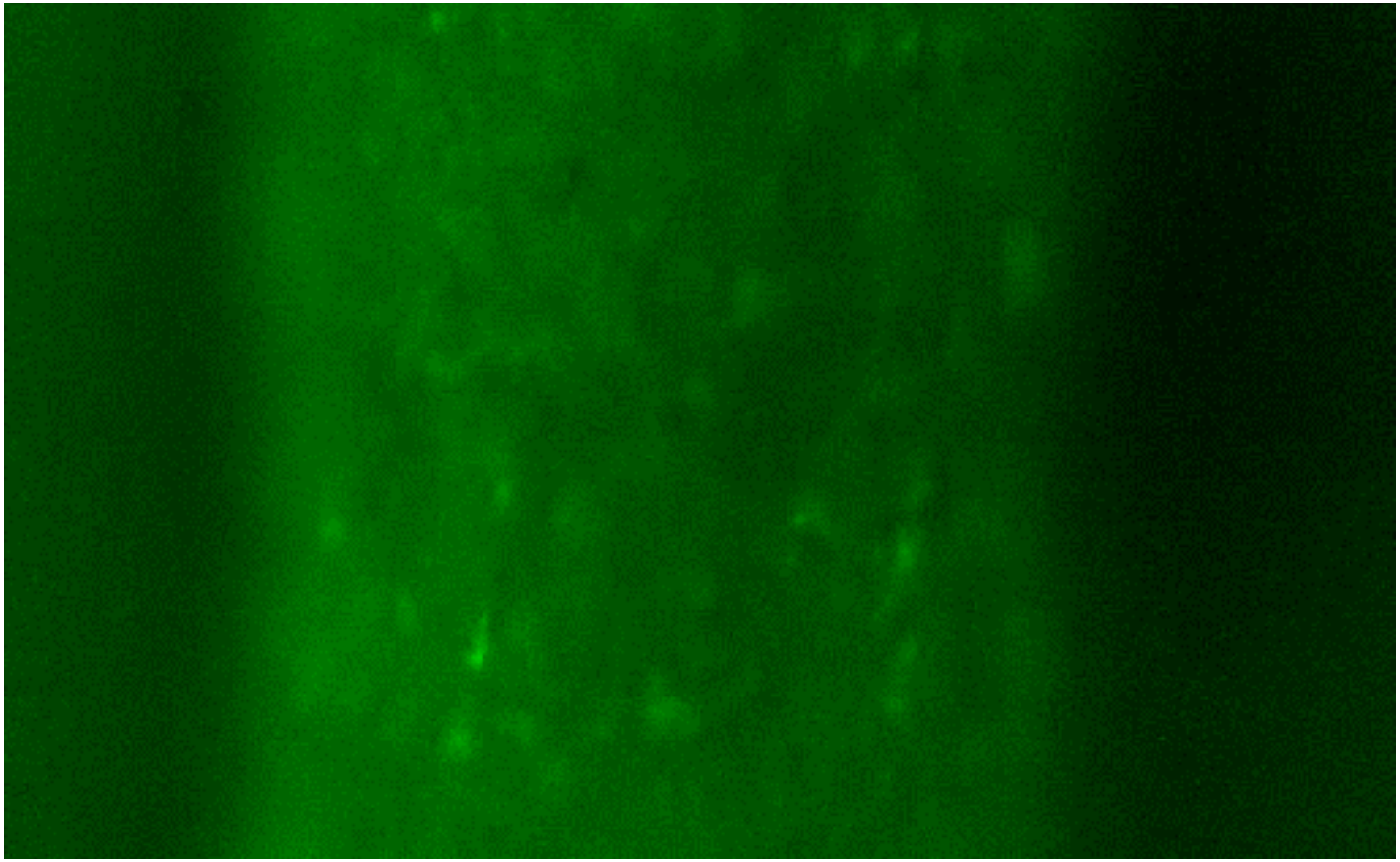

Supplementary Video S8: EHTs were fabricated with cardiomyocytes differentiated from hPSCs expressing a GCaMP6f calcium indicator.
