# Supplementary material for: Reproducibility of drug-induced effects on the contractility of an engineered heart tissue derived from human pluripotent stem cells
Source: Front Pharmacol. 2023 Jul 4;14:1212092. doi: 10.3389/fphar.2023.1212092 (PMC10352809; doi:10.3389/fphar.2023.1212092)
Supplement: Supplementary file 5 [file DataSheet3.pdf]

frontiers

Detailed Materials and Methods

Reproducibility of drug-induced effects on the contractility of an engineered heart tissue model with human pluripotent stem cells

Ayesha Arefin*, Melissa Mendoza, Keri Dame, M. Iveth Garcia, David G. Strauss & Alexandre J.S. Ribeiro*

*** Correspondence:**
Ayesha Arefin: rfnayesha@gmail.com
Alexandre J.S. Ribeiro: axribeiro3@gmail.com

1. Cell culture platforms- Page 02
2. Thawing cryopreserved hPSC-cardiomyocytes to use in EHT fabrication- Page 05
3. Preparation of media and reagents- Page 07
4. Instrumentation- Page 12
5. Operation and settings for different experimental assays- Page 15
6. Testing drug effects- Page 19
7. Data analysis- Page 26
8. Detailed methods and materials references- Page 27
9. Method figures- Page 29
10. Method tables- Page 44

1. CELL CULTURE PLATFORMS

Fabrication of engineered heart tissues (EHTs)

Unless indicated otherwise, all materials used in the fabrication of EHTs were purchased from the former EHT Technologies GmbH (Hamburg, Germany, EU, 2018), now DiNAQOR Deutschland GmbH (Hamburg, Germany, EU) and all supplies and reagents were decontaminated following standard procedures. EHT fabrication consisted of a two-day process, where Silicon racks (DiNAQOR Deutschland GmbH, C0001) and Teflon spacers (DiNAQOR Deutschland GmbH, C0002) were cleaned and sterilized (autoclaved) at least one day prior to fabrication according to vendor instructions. Cool racks (Neta scientific inc, NJ, USA, 432061) for 15 mL conical tubes (Thermo Fisher Scientific, MA, USA, 432061) and for 2 mL Eppendorf tubes (Thermo Fisher Scientific) were placed in the refrigerator overnight. A 2% Agarose (Invitrogen, Waltham, MA, USA, 16500100) solution and 10x DMEM medium (Gibco, MA, USA, 12100056 13.4 gram) were then prepared as detailed ahead. In this study, EHTs were generated from two different human pluripotent stem cell (hPSC) lines including hPSC-cardiomyocytes2 (iCell2) from Fujifilm-Cellular Dynamics International, WI, USA, and in-house differentiated human WTC-11 GCaMPf hPSC line, which was a gift from Dr. Bruce Conklin (Gladstone Institute of Cardiovascular Disease and UCSF). iCell plating medium (Fujifilm-Cellular Dynamics International, CMM-100-110-001) was moved from a -20°C freezer into a 4°C refrigerator (if using iCell2) or prepared RPMI complete (Gibco, 11875101) medium as instructed ahead (if using WTC11) and stored it in the refrigerator at 4°C. Fresh aliquots of penicillin/ streptomycin (Gibco, 15140) and L-glutamine (Gibco, 25030-081) were also transferred from -20°C to the refrigerator at 4°C. The following day, heat-inactivated fetal calf serum (Gibco, 16140071) and heat-inactivated horse serum (Life Technologies, CA, USA, 26050088) were moved from the -20°C freezer into the refrigerator at 4°C. A bucket with ice was used for temporary storage of Matrigel (Corning, NY, USA, 354234) aliquots and fibrinogen (Sigma-Aldrich, MO, USA, F8630) aliquots were placed it into the fridge at 4°C for at least 30 minutes prior to use. Plating medium (if iCell2) or RPMI complete medium (WTC11) was kept at room temperature for at least one hour before use. Once all the reagents were thawed, different formulations of media were prepared and used in the fabrication process (10 mL of 2x medium, 5 mL of NKM medium and an appropriate amount of EHT medium) as detailed ahead. Per rack of four EHTs (**Method Figure 1**), 2 mL (4x0.5 mL) of EHT medium was used for releasing the tissue from the agarose-based cast and 6 mL (4x1.5 mL) of EHT medium was used for the final medium change once the fabrication was completed. After medium was prepared, the 15 mL cool rack was placed on top of ice in the bucket and a 15 mL conical tube with prepared medium was placed on the rack to keep it cold. A 24-well plate (Thermo Fisher Scientific, 144530) and a sterile metal container with the Silicon racks and Teflon spacers were brought into the biosafety cabinet. The agarose solution (detailed ahead) was transferred from an oven to the cabinet and checked for its homogeneity to ensure its quality. If any clumps were observed, the solution was microwaved for 10 seconds to ensure a homogeneous consistency. The agarose solution was dispensed into a 24-well plate to prepare solid agarose trenches that served as molds for the EHTs to be formed around the Silicone racks (**Method Figure 2**). In detail, 1.6 mL of agarose solution was added gently into each well, while avoiding formation of bubbles, totaling 4 wells at one time and Teflon spacers were placed vertically on the top of the wells (each spacer

should fit in the middle of the wells and should not move). After 20 minutes for agarose gelation, the Teflon spacer was carefully removed from the wells, leaving a trench per well where each pair of posts of the Silicon rack would fit. The Silicon rack was placed inside the agarose trenches and ensured it was stable, centered and did not touch the [agarose walls or bottom surface. The thrombin (Sigma-Aldrich, SIGT6634-100UN), ROCK inhibitor (Y-27632) (Thermo Fisher Scientific, SCM075) and Matrigel aliquots, along with eight empty PCR tubes were placed onto the 2 mL cool rack on ice. A fibrinogen aliquot was warmed up in hand for a few seconds and transferred into the biosafety cabinet at room temperature. Next, the EHT mastermix was prepared to later include cells and form a tissue around and between the extremities of the Silicon racks in each agarose trench. To prepare the mastermix 147 µL of 2x DMEM, 264.0 µL of Matrigel, and 2.6 µL of Y-27632 were added in a cold 15 mL tube. Tips were changed and 66.8 µL of fibrinogen solution was added to the mastermix. The mastermix was gently homogenized by pipetting up and down with a P1000 pipettor for 10 to 15 times. 80 µL (per 4 tissues) of mastermix was then transferred to a 2 mL tube and kept in a cool rack. Next, 3 µL of thrombin solution was transferred to eight PCR tubes, which were also kept in the cool rack. Cells (iCell2 or WTC11) were thawed, counted, and centrifuged as detailed ahead. 100 µL of NKM medium was added slowly with gentle rocking the tube back and forth and gentle pipetting to resuspend the pelleted cells per every million cells counted. For example, 4.4 million cells were used to generate four tissues and 440 µL of NKM medium was added. 20 µL of mastermix solution was added per 110 µL of NKM+cell mixture to prepare tissues. A total of 80 µL of mastermix was used per every four tissues. Then, 97 µL of tissue suspension (NKM+cell+mastermix) was added to one tube containing 3 µL of thrombin solution by placing the pipet tip all the way to the bottom of the tube and slowly dispensing the tissue suspension without creating any bubbles. The total volume (97- 100 µL) of this solution was transferred without mixing, without changing the tip or changing the volume of the pipette, and carefully added it to the bottom of the agarose trench containing the Silicon racks in the 24-well plate. Pipette tips were changed after filling each agarose trench with tissue suspension. When the volume of tissue suspension was not enough to fill leftover trenches with posts, mastermix with NKM medium without cells and thrombin was added to those trenches to keep the rack balanced. After adding the tissue suspension to the trenches, the 24-well plate was gently rocked back and forth once and placed into the cell culture incubator (environmental conditions detailed ahead) for one hour. 500 µL of EHT medium was added to each well, and the plate was gently swirled and placed back into the incubator for 30 minutes. During this time, a new 24-well plate with 1.5 mL of fresh EHT medium per well was prepared and incubated for equilibration for at least 15 minutes. After 30 minutes, both the casting plate with agarose trenches and the medium plate equilibrating in the incubator were brought into the biosafety cabinet. The Silicon racks were carefully and slowly removed and were transferred to wells of the new 24-well plate with equilibrated EHT medium. The plates with the tissues were transferred back to the incubator. EHT medium was changed three times a week for at least 21 days, and force was routinely measured using the EHT measuring system (DiNAQOR Deutschland GmbH, A0001, first generation). Once each tissue passed quality control criteria detailed ahead, preplanned acute or long-term drug exposure experiments were executed.

EHT Medium Change

EHTs were maintained by changing medium every Monday, Wednesday, and Friday at a consistent time between 11 to 12 am. During every media change, previously aliquoted heat-inactivated horse serum was thawed from -80°C storage, and fresh EHT medium was aseptically prepared (detailed ahead), and 1.5 mL of new medium was pipetted into each well of a new 24-well plate. Per batch of EHTs, two plates were used for medium changing purposes. Both plates were kept in the incubator and appropriately labeled as plate one and plate two. While maintaining the EHTs in plate one, plate two was the plate for medium equilibration prior to exposing the tissues to fresh medium. After adding freshly prepared EHT medium to plate two, the plate was equilibrated for at least 15 minutes in the incubator. Both plates were then placed inside the biosafety cabinet and tissue racks were carefully transferred to the equilibrated fresh medium in plate two. On the following media-changing day, the same process was followed.

Culturing hPSC-cardiomyocytes (iCell cardiomyocytes2) as monolayers

Surface-treated 24-well plates (Thermo Fisher Scientific, 142475) were used to culture iPSC-cardiomyocytes as monolayers according to the layout (**Method Figure 14**). This layout aimed to allow integration of electrical Pacing electrodes (DiNAQOR Deutschland GmbH, P0001) on the plate to electrically stimulate monolayers. Prior to seeding cells, the surfaces of the wells were coated with fibronectin (Sigma-Aldrich, F1141) by incubating one mL per well of a 12.5 µg/mL solution in PBS with 0.02% gelatin (StemCell Technologies, Vancouver, Canada, 07903) for one hour and 30 minutes in the cell culture incubator. After adding 2 mL of PBS at room temperature per well and keeping it in the cell culture incubator at 37°C, fibronectin-coated wells were ready to use within one hour to two days. PBS was aspirated from wells prior to seeding cells after being thawed.

Before thawing iCell cardiomyocytes2, plating medium and maintenance medium (Fujifilm-Cellular Dynamics international, CMM-100-120-001) were thawed overnight in the refrigerator at 4°C. Plating medium was used once after thawing, and maintenance medium was stored in the refrigerator at 4°C and not used beyond two weeks of storage. Cells were thawed and centrifuged according to vendors' instructions. After obtaining a cell pellet in a 50 mL conical tube, cells were resuspended in plating medium of enough volume to seed 156,000 viable cells/cm^2. Once all liquid was aspirated from the coated wells, a clonal ring (Sigma-Aldrich, C2059) was placed in the center of each well. Plating medium of 500 µL with resuspended cells were added to the interior of each ring and plates were left in the cell culture incubator (37°C, 7% CO_2, 40% O_2) for four hours to allow cells to attach to the bottom of the wells. During this time, maintenance medium was equilibrated on a second empty plate in the incubator. The clonal ring was then carefully removed, and the plating medium was aspirated before adding one mL of pre-equilibrated maintenance medium to each well. To transition from the commercially available culture medium to the medium used to maintain EHTs without aprotinin (Sigma-Aldrich, A1153), daily medium changes were made using different medium compositions according to the following schedule:

- Day 0: Cells Maintenance Medium
- Day 1: Maintenance Medium + 25% EHT medium (without aprotinin)
- Day 2: Maintenance Medium + 50% EHT medium (without aprotinin)

- Day 3: Maintenance Medium + 75% EHT medium (without aprotinin)
- Day 4: 100% EHT medium (-aprotinin)
- Day 5: Ready for experiments

2. THAWING CRYOPRESERVED HPSC-CARDIOMYOCYTES TO USE IN EHT FABRICATION

Procedures are described per vial, but volumes used were adjusted when using more than one vial.

iCell Cardiomyocytes2 (iCell2):

Cryovials with cells were stored in liquid nitrogen after being received from the manufacturer. When ready to use for EHT fabrication, cryovials were transferred in a box filled with dry ice to the cell culture room and cells were kept on dry ice for less than 60 minutes. When ready to thaw, the cryovial was partially submerged in a water bath at 37°C with gentle agitation for about one minute with frequent inspection of the melting contents until only a sliver of ice was left in the middle. The cryovial was carefully wiped with a sterile cloth soaked in 70% ethanol after clearing any wetness from its surface and brought aseptically to the biosafety cabinet. Following vendors' instructions, the contents of the cryovial was gently transferred into a 50 mL conical tube, the cryovial was rinsed with one mL of plating medium (the vial was continuously rotated while adding medium to wash cells from the wall) and added it to the 50 mL tube (dropwise, each drop every 4-5 seconds). The drops were added to the inner surface of the conical tube while keeping a distance from the bottom liquid and gently swirled at the end. Next, 16 mL of plating medium was slowly added at room temperature to the tube, following a drop wise addition technique for 30-60 seconds for the first one mL, and then added the remaining amount within 30 seconds. The cell suspension was then gently mixed by slanting the tube back and forth two to three times after sealing it. 20 µL of the suspension was used for the purpose of cell counting with a 0.4% trypan blue stain solution (Aligned Genetics Inc, Anyang, South Korea, T13001). A total of four independent counts were performed using an automated cell counter (Aligned Genetics Inc, LUNA-IITM) and averaged the separate measurements to calculate the volume of cell suspension to be used during EHT fabrication. After centrifuging the tube at 200 x g for 5 minutes, the tube was transferred back to the biosafety cabinet to carefully aspirate the medium and kept it on ice after resuspending in the appropriate volume of NKM medium.

WTC-11 GCaMPf hPSC-Cardiyomyocytes

The procedure described for thawing iCell2 cardiomyocytes was also followed for cardiomyocytes derived from WTC-11 GCaMPf hPSC, but a different thawing medium was used: RPMI complete medium with ROCK inhibitor and its preparation is described ahead.

Tip: The thawing process was started once all other necessary EHT fabrication components were completed, thereby minimizing the amount of time cells were left as a pellet after centrifugation.

Culture and maintenance of WTC-11 GCaMPf HPSC- cardiomyocytes

The WTC-11 GCaMPf reporter hPSC line was cultured in mTeSR hPSC culture medium (STEMCELL Technologies, 85850) on hESC-Qualified Matrigel-coated (Corning, 354277) six-well plates (Greiner Bio-One CELLSTAR, Kremsmünster, Austria,657160) and passaged with Accutase (Innovative Cell Technologies, Inc, San Diego, CA, USA, AT-104) for expansion and maintenance. To dissociate cells, cultures were rinsed 2x with sterile PBS (Thermo Fisher Scientific, 14190250) followed by 5 minutes incubation with Accutase at 37°C. Once cells were appropriately dissociated, determined by visual inspection of the plate, the dissociation enzyme was quenched with mTeSR medium, transferred to a 15 mL tube, and centrifuged for 3 minutes at 100 x g. The supernatant was aspirated, and the pellet was resuspended in mTeSR supplemented with 5 µM ROCK inhibitor (Y27632 Tocris Bioscience, Bristol, UK, 1254) for about 20 hours after seeding. The passage ratio was experimentally determined; here, cells were passaged at a 1:30 split for maintenance. An expanded stock was frozen in Bambanker freezing medium (FUJIFILM Wako Chemicals USA, Corp., VA, USA, NC9582225) for cryopreservation and for continued experimental use.

Directed differentiation to cardiomyocytes (WTC-11 GCaMPf hPSC-cardiomyocytes)

A cardiac differentiation protocol was used based on temporal modulation of WNT signaling controlled by small molecules (1) followed by cardiomyocytes purification using metabolic selection (2). hPSCs were thawed, maintained, and passaged 3-5 times on Matrigel-coated 6-well plates before initiating differentiation. To prepare for differentiation, mature hPSC colonies were singularized in Accutase at 37°C for 8 minutes, quenched, centrifuged, resuspended in one mL mTeSR, and counted using the LUNA II automated cell counter. Cells were then diluted to the appropriate volume and seeded at a density of 12,500 cells per cm^2 in 6-well plates and cultured for 4-5 days in mTeSR, where medium was changed daily until nearly confluent. When colonies appeared dense and mature, cultures were exposed to 7 µM CHIR99021 (LC Laboratories, Boston, MA, USA, C-6556) in RPMI 1640 supplemented with B27- insulin (Gibco™, A18956-01) for 48 hours, followed by 24 hours in RPMI with B27- insulin without CHIR. At 72 hours, WNT inhibition was carried out with the addition of 5 µM IWP2 (Biorbyt, Cambridge, UK, orb146227) in RPMI 1640 supplemented with B27-insulin for two days. From day 5 to day 11, cultures were maintained in RPMI 1640 with B27-insulin, where visual beating activity emerged and increased from approximately day 7 to day 10. On day 11, metabolic purification began with glucose starvation by culture in RPMI 1640-Glucose with B27+insulin, resulting in a relatively pure population of cardiomyocytes expressing actin and troponin T after 3-4 days. The glucose starvation period was stopped when non- cardiomyocytes had died off and before cardiomyocyte beating activity was affected. After a 24-hour recovery period in RPMI 1640+Glucose with B27+insulin, the hPSC- cardiomyocytes were passaged using accutase to a monolayer culture on fibronectin-coated 6-well plates for 28 days in maturation medium containing RPMI-Glucose with B27+insulin, 4 mM lactate (FUJIFILM Wako Chemicals USA, 129-02666), and 100 nm T3 (Sigma-Aldrich). On day 28, cells were dissociated using accutase and cryopreserved in 90% FBS / 10% dimethyl sulfoxide (DMSO) freezing medium.

Fluorescent labeling of WTC-11 GCaMPf hPSCs and hPSC-cardiomyocytes

Cells (WTC11 hPSCs and WTC11 hPSC-cardiomyocytes) were fluorescently labeled with immunocytochemistry against marker proteins of pluripotency (hPSCs) or cardiomyocyte differentiation (hPSC-cardiomyocytes). Labeled markers of pluripotency were the TRA-1-60 cell surface antigen and the transcriptional factor homeobox protein NANOG. Sarcomeric proteins alpha-actinin and troponin-T were labeled as markers of cardiomyocyte differentiation. After being washed with PBS, cells were fixed with 4% (v/v) paraformaldehyde (Electron Microscopy Sciences) in PBS for 15 minutes and then permeabilized in 0.1% (v/v) triton X-100 (Sigma) for 15 minutes more. Then, after washing cells three times with PBS with 1% bovine serum albumin (Sigma), we blocked cells with a PBS solution of 10% (w/v) normal goat serum (Thermo Fisher Scientific) for one hour and incubated samples overnight at 4 ºC with primary antibodies against the mentioned proteins in a solution of PBS with 1% bovine serum albumin. Primary antibodies were diluted as follows for incubation: i) anti-TRA-1-60 mouse monoclonal (Abcam) 1:500; ii) anti-NANOG rabbit polyclonal (Abcam) 1:500; iii) anti-alpha-actinin mouse monoclonal (Sigma); iv) 1:2500 anti-troponin T mouse monoclonal (Thermo Fisher Scientific) 1:400. The following days, cells were rinsed three times with a solution of PBS with 1% bovine serum albumin and incubated for two hours in a solution containing the following secondary fluorescent antibodies (ThermoFisher Scientific; 1:200 dilution) in PBS and 1% bovine serum albumin: i) goat anti-mouse IgG (H+L) Alexa Fluor™ 488 and donkey anti-rabbit IgG (H+L) Alexa Fluor™ 568. After washing again three times with a solution with 1% bovine serum albumin in PBS, we incubated cells for 15 minutes with a solution of Hoechst 33342 at a concentration of 10 µg/mL in PBS and washed again three times in PBS before mounting cells with VECTASHIELD mounting medium (Vector Laboratories).

3. PREPARATION OF MEDIA AND REAGENTS

Different types of media were used in the different stages of EHT fabrication, maintenance, and use (**Method Table 1**) storage, thawing and preparation procedures were followed to handle medium reagents to ensure a reproducible outcome of the performed experiments. In the following sections, the storage, thawing, aliquoting and preparation of all the necessary reagents used in this study are described. Various media types for specific purposes were prepared from different components on the day of use, including EHT casting medium (NKM), EHT medium, EHT medium without aprotinin, EHT medium with 4% serum and without any serum, iCell cardiomyocytes2 plating medium, and thawing medium for WTC-11 GCaMPf reporter hPSC-cardiomyocytes. Some of the media types that were reused after being kept under the recommended condition for a finite number of days, as instructed by the manufacturer, such as 1x DMEM (Sigma-Aldrich, D5546), 10x DMEM and iCell cardiomyocytes2 maintenance medium. The various media used throughout the study are listed in **Method Table 1**.

HI Fetal Bovine/Calf Serum and HI Horse Serum

Heat-inactivated fetal calf serum and horse serum are the key components for EHT generation as noted by system developers. Serum was aliquoted, thawed, and used only once to avoid potential adverse effects of repeated freeze/thaw on cellular properties. Upon receiving it, serum was stored in

the freezer at -20°C. Once needed for aliquoting in smaller volumes for use, a 500 mL of a serum bottle was thawed at 4°C for two to three days. After fully thawed, the serum was aliquoted using standard sterile techniques in a biosafety cabinet. Fetal calf serum and horse serum were aliquoted (3 mL) into prelabeled 15 mL conical tubes, while keeping the original serum bottle on ice. The aliquots were stored in the freezer at -80°C until needed.

L-glutamine (200 mM)

L-glutamine was added to NKM medium, which was used during the EHT fabrication. Initially, the 100 mL bottle was thawed in the refrigerator at 4°C. Following standard aseptic techniques in a biosafety cabinet, 5 mL aliquots were prepared and stored at -20°C. Once thawed to make NKM medium, an aliquot was stored at 4°C for up to one month to use when needed.

Penicillin/Streptomycin

Penicillin/streptomycin was added to EHT medium for tissue formation. The 100 mL bottle was thawed at 4°C. Following standard aseptic technique in a biosafety cabinet, aliquots of 5 mL were prepared and stored at -20°C. Once thawed, an aliquot was stored 4°C for up to one month to use when needed.

Aprotinin from bovine lung (33 mg/mL)

Aprotinin, which prevented fibrin degradation, was required for EHT medium and fibrinogen preparation. After receiving it from the manufacturer, the stock aprotinin was stored at 4°C. To prepare aprotinin in solution, 1 bottle of aprotinin (10 mg) was dissolved in 303 µL of water for injection (Sigma-Aldrich, A12873-01) to make a 33 mg/mL solution. Aliquots of 20 µL were prepared and stored at -20°C until needed. Freshly prepared aprotinin was used to make fibrinogen solutions.

Fibrinogen (200 mg/mL)

Fibrinogen was a critical component for successful EHT formation, as noted by EHT developers. A homogeneous solution indicated successful dissolution of fibrinogen powder. The fibrinogen preparation process was initiated by autoclaving 100 mL of 0.9% NaCl (Labchem Inc, PA, USA, LC234604) and stored at room temperature, only being used under sterile conditions. Prior to making the fibrinogen solution, the bottle containing 0.9% NaCl was warmed in the water bath at 37°C for at least 15 minutes. Once warm, the 0.9% NaCl solution was transferred to a 50 mL tube and (72.1 x 5) 360.5 µL of aprotinin added per 5 mL NaCl-solution (needed two aprotinin bottles of 10 mg each). Using a sterile spoon in the biosafety cabinet, fibrinogen (one gram) was transferred to a 50 mL sterile conical tube and fibrinogen clumps were broken down into crude powder with a sterile metal spatula or serological pipette and 5 mL of 0.9% NaCl was slowly added to the fibrinogen powder.

After, the lid of the conical tube was tightly secured, and the solution was kept it in a water bath at 37°C. Under sterile conditions and every 10 to 15 minutes, the tube was gently inverted to avoid the formation of any bubbles to dissolve the fibrinogen for approximately one hour. The homogeneous solution was then split into 100 µL aliquots that were stored at -80°C.

Tip: Fibrinogen was often received in larger clumps, which were turned into powder before dissolving. Solutions were discarded when fibrinogen did not fully dissolve following the described procedure (**Method Figure 3**).

Matrigel

Matrigel was stored at -80°C upon receiving. To aliquot the stock solution, the bottle was transferred to an ice bucket that was previously kept at 4°C for at least one hour and transferred back to 4°C to slowly thaw overnight. In the meantime, supplies including the pipette tips, a tube rack and storage box used for handling Matrigel were also refrigerated at 4°C. The following day, Matrigel aliquots of 350 µL were prepared in the biosafety cabinet, while minimizing exposure to room temperature. In this process, tips were changed often to ensure they did not warm up and tubes were kept on ice. Matrigel aliquots were then transferred to a pre-labeled cold storage box and stored at -80°C.

Tip: Matrigel aliquots were thawed on ice at 4°C for 2 hours on the day of EHT fabrication.

Thrombin, Bovine (100 U/mL)

The Thrombin enzyme is necessary to form the fibrin gel during EHT formation. Thrombin was stored at -20°C upon receiving it from the vendor. To prepare a solution from 100 UN thrombin powder, a solution of PBS pH 7.4 (1X) was aseptically prepared without CaCl, MgCl (Life technologies, 10010049) and water for injection. Specifically, 600 µL of PBS pH 7.4 (1X) without CaCl, MgCl with 400 µL of water for injection were combined in a 2 mL Eppendorf tube. One mL of PBS solution was then added to the thrombin tube containing the powder. The solution was mixed with a one mL pipettor by pipetting up and down 5 to 10 times. Then, the thrombin solution was aliquoted at 20 µL volume in PCR tubes and stored at -20°C for up to one year. During the EHT formation process, the unused excess solution was discarded after thawing and not refrozen to avoid potential protein degradation due to freezing and thawing.

ROCK inhibitor, Y-27632, dihydrochloride salt (10 mM)

ROCK inhibitor was required to formulate EHT medium, as instructed by the developers. Y-27632 was received in batches of 5 mg, which were stored at -20°C. To prepare a 10 mM concentration, 1.478 mL of sterile distilled water (Gibco, 15230-162) was added directly to the Y-26632 container, mixed well by pipetting up and down several times and aliquoted into PCR tubes at volumes of 3 µL, which were then stored at -20°C for up to 1 year.

10x Dulbecco's modified eagle medium (DMEM)

DMEM powdered medium packets were stored at 4°C upon receiving. To prepare 10x DMEM medium, all the contents of one package was transferred to a 150 mL medium bottle (Corning, CLS431175) and 100 mL of water for injection was added. the bottle containing the medium solution was then swirled until most of the DMEM powder was dissolved and a 50 mL serological pipette was used to thoroughly mix and dissolve the rest of the powder. Medium was stored in the refrigerator at 4°C for up to one month.

2x DMEM medium preparation

2x DMEM medium was prepared on the day of EHT formation. Two to three hours prior to the preparation, heat-inactivated horse serum was thawed, and penicillin/ streptomycin was placed at 4°C. After both components were fully thawed, 10 mL of 2x medium was aseptically prepared in 15 mL sterile conical tubes and kept them on ice. Further, two mL of horse serum and 0.2 mL of penicillin/streptomycin was added to two mL of 10x DMEM solution. Finally, 5.8 mL of water for injection was added and mixed well by pipetting up and down several times. This solution was stored on ice at room temperature until needed for the EHT generation step. Unused medium was discarded and not reused.

EHT casting medium

Termed in the literature as ECM (3) or NKM (4) medium, EHT casting medium was prepared on the day of EHT fabrication. Two to three hours prior to its preparation, aliquots of heat-inactivated calf serum, penicillin/streptomycin, and 200 mM L-glutamine were thawed at 4°C. After fully thawed, batches of 5 mL of EHT casting medium were aseptically prepared in 15 mL conical tubes, which were kept on ice. For this purpose, 500 µL calf serum, 50 µL penicillin/ streptomycin, and 50 µL L-glutamine were added to 4.4 mL of 1x DMEM medium in a 15 mL conical tube, which were kept on ice until needed. Unused medium was discarded.

EHT medium

EHT medium was also prepared fresh on the day of EHT fabrication and every time for every medium change during tissue maintenance on Mondays, Wednesdays, and Fridays. Two to three hours prior to the preparation of EHT medium, heat-inactivated horse serum was thawed at 4°C. The volume of medium to be prepared was determined by the number of tissues being fabricated or maintained. Per 10 mL of EHT medium, 10% horse serum (one mL), 1% penicillin/ streptomycin (100 µL), 0.1% Insulin (Sigma-Aldrich, I9278) (10 µL) and 0.1% aprotinin (10 µL) was aseptically added into 8880 µL of 1x DMEM medium in a 15 mL conical tube.

EHT medium without aprotinin

EHT medium without aprotinin was used in experiments involving cells cultured as monolayers in traditional two-dimensional settings. Fresh medium was prepared every time before medium changes following the previous procedure for EHT medium preparation, but without adding aprotinin.

Tip: EHT medium was made fresh every time before medium changes. Horse serum and aprotinin aliquots were not frozen and thawed, and the excess of these components and medium were discarded after preparation of EHT medium. Record of the horse serum lot numbers (lot# 1999127) were kept and maintained throughout the study, given that developers previously reported that abnormal batches of horse serum could cause unexpected biological effects in the EHTs.

4% Serum and Serum free EHT- medium

EHT medium with reduced serum was used for experiments involving long-term drug exposure to EHT and was also made fresh before each use. After meeting established quality control criteria, EHTs were maintained in 4% serum EHT medium during the whole experimental period, when exposing them to drug; during drug exposure, serum free EHT medium was used. This medium was prepared similarly to the EHT medium, other than a reduction of horse serum from 10% to 4%.

iCell cardiomyocytes2 plating medium

Upon receiving the plating medium from the supplier, it was stored at -20°C. The day before fabricating EHTs, the medium was thawed in the refrigerator at 4°C overnight.

iCell cardiomyocytes2 maintenance medium

This medium was used briefly after removing the plating medium and before transitioning to EHT medium without aprotinin for culturing cells as monolayers. After receiving, the medium was stored at -20°C. The day before seeding cells in monolayers, it was thawed in the refrigerator at 4°C overnight and kept under those conditions for use within one week.

Thawing medium for WTC-11 GCaMPf hPSC-cardiomyocytes

For thawing WTC-11 GCaMPf hPSC-cardiomyocytes, complete RPMI medium was used. The B27 supplement (Gibco, 17504044) was thawed in the refrigerator at 4°C and added at a 1:50 ratio to the RPMI medium along with penicillin/streptomycin at a ratio of 1:100. ROCK inhibitor Y-27632(10 mM) was added at a 1:1000 dilution in medium and used for cell seeding.

Preparation and maintenance of 2% agarose solution

Agarose was stored at room temperature. Before 2% agarose preparation, a dedicated 100 mL Pyrex flask was autoclaved. 100 mL sterile PBS pH 7.4 (1X) without $CaCl_2$, or $MgCl_2$ was then aliquoted into the flask. 2 grams of agarose were weighed and added to the PBS flask. A microwave oven was used to completely dissolve the agarose. After dissolution, the flask containing 2% agarose was stored in a heated cabinet at 60°C. The agarose solution could be stored up to one week.

Tip: Agarose was dissolved in PBS rather than water, as developers noted that water may cause osmotic shock in cells. Usually, the 2% agarose solution was prepared the day before tissue generation, stored in a heated cabinet, and kept in a dedicated glass flask for autoclaving. The glass flask was never washed with detergent, only with running tap water. The flask was only opened inside the hood to keep the contents sterile.

Optimizing the composition of Tyrode's solution

Initially, Tyrode's solutions with different compositions were tested with a cardiomyocyte monolayer. The selected composition worked best for both monolayers and engineered heart tissue (5). The customized formulation was ordered from a small biotechnology (Boston BioProducts, Inc., MA, USA, C-7650F, C-7651F) company. The components are listed in **Method Table 2**.

Tyrode's solution preparation

A 0.6 mM Tyrode's solution was used for contractile function modulating drug exposure, as well as for washing the tissues. 150 mL of 0.6 mM of Tyrode's solution was prepared by adding 18 mL of Tyrode's solution containing 5 mM Calcium to 132 mL of Tyrode's solution containing no calcium. The solution was stored in a refrigerator at 4°C for up to one month and used when needed.

4. INSTRUMENTATION

EHT measuring system assembly

The EHT measuring system has several components (**Method Figure 4**). A camera for recording tissue contractility and associated hardware, an environmentally controlled chamber, a gas mixture flask, gas mixture controller, and a computer that has the contractility recording software. Upon arrival of the system, the optical recording hardware (the camera and the gas- and temperature-controlled incubator) was unwrapped as per instructions and the Thermic mass flow controller (Vögtlin Instruments – flow technology, Muttenz, Switzerland, GCR Compact Regulator, customized) was placed closer to the gas wash flask (Fisher Scientific Co LLC, Pittsburgh, PA, USA, 09-841-092) for controlling the proper gas mixing. The Thermic mass flow controller was installed by connecting it to N_2, O_2, and CO_2 gas lines. Bubble tubing (of variable diameter as the tubing inlet diameter in the Thermic mass flow controller was difficult to find in the USA) was used to connect

the gas input. Bubble tubing (Allied Healthcare B and F 100 Ft. Smooth Bore Disposable Oxygen Tubing, 64203) was used for output and a T adapter was used to merge all three-gas outlet tubing into one outlet tubing. A 6 mm outer diameter silicon tubing was used as a merged outlet tubing and was connected to the outlet of the gas flask with an inline filter. To do so, the glass enclosure was unscrewed from the front part, and the bubble wrap, fixation screw, and lens cap was removed from the camera. The enclosure was put back and the CPU, keyboard, monitor, and mouse was connected. The instrument data cable was connected to the PC (white: 37 pol DUSB cable), the power cable to the electric multiple array (provided by the vendor), the camera (red cable) to the front right next to the LED (away from the other USB connection panel), and the axis system to PC, as instructed: upper two left Y, right X, and the bottom right Z. Finally, an appropriate converter was used to connect the system to power the power source.

Thermic mass flow controller set up

The thermic mass flow controller was used to control the humidity and required gas inside the gas- and temperature-controlled incubator of the EHT measuring system. Bubble tubing was used to connect N2, O2, and CO2 gases to the controller and an intermediate valve was installed to control the gas flow (on/off). Bubble tubing and a T-adapter were used to merge three outlets into one and a filter in the outlet tubing connected it to the gas mixture wash flask. The wash flask for gas mixture was filled with ultra-pure distilled water (500 mL). For short-term use or daily use, the environmental conditions were first set up by turning on the thermic mass flow controller. Then, the valves for all three gases were turned on. The gas flowrate was adjusted to O2: 200 mln/min, N2: 235 - 2635 mln/min, and CO2: 35 – 65 mln/min. Flow increased when the knob was rotated counter-clockwise. Experiments were performed when the CO2 percentage reached 7%. Experiments that required leaving the EHTs inside the EHT measuring system for over 2 hours were not performed. Per developer instructions, it was possible to keep the Thermic mass flow controller going and to stabilize the gas inside the gas- and temperature-controlled incubator inside the EHT contractility system for several days. This study was unable to control the gas flow due to various leakage issues, and thus did not explore the long-term use of the Thermic mass flow controller and the EHT contractility system.

Tip: The gas line was occasionally checked for visibility of any water which could be due to the back pressure after the gas controller was turned off. If there was any water, compressed air was used to remove the water.

Sterilization and storage of Silicon racks and Teflon spacers

Silicon racks and Teflon spacers were shipped in a 24-well plate (**Method Figure 1**). Following instructions from developers, racks and spacers were cleaned, sterilized, and stored at the end of every experiment. After experiments, the racks and spacers were first rinsed under running water (tap water) to get rid of the agarose or any tissue debris, specifically checked them against light and used a tweezer to remove any tissue debris that was still attached to the posts. Using a hot plate and a clean beaker, distilled water was boiled and then Silicon racks or Teflon spacers were added to the boiling water for 10 minutes. The process was then repeated with fresh distilled water once more. They were

dried in a 70°C oven for approximately 15 minutes. The Silicon racks were stored in an autoclavable metal box (Without a metal box, the Silicon rack turned yellowish. This could be autoclave-specific but using a metal box solved this problem). Individual autoclave packs were used to wrap the spacers for autoclaving. The autoclave setting should not exceed 110°C, as recommended by the developer.

Tip: Designating separate beakers for boiling Teflon spacers and Silicon racks from other lab activities helped to keep the beakers free of soap and detergents. The vendors recommended cleaning the beakers with water, and not to use detergents or salts at any point. Silicon racks were placed upside down during autoclaving, so the posts would not bend. As instructed by developers, spacers have the capability of multiple uses; however, here reuse of the Silicon racks was limited depending on the type of experiment. As the Silicon rack constructing polymer, PDMS absorbs drugs (6), absorption of dye was notably visible in the Silicon racks even after the aforementioned cleaning and sterilization process after long-term drug treatment experiments. To avoid any unwanted carry over of previously added drugs, new racks were cleaned and used for each long-term experiment (**Method Figure 5**).

Pacing electrodes

Pacing electrodes (DiNAQOR Deutschland GmbH, P0001) were usually stored in a 24-well plate at room temperature. The Pacing electrodes were cleaned and sterilized prior to starting and at the end of an experiment. When ready to clean, Pacing electrodes were rinsed, and the hinges were taken out using a screwdriver provided by the developer and stored in a dry box. A deep square glass tray was filled with distilled water and a plastic tube holder rack was placed in the tray for cleaning (**Method Figure 6**). Pacing electrodes were placed in such a way, so they were hung and submerged, making sure that the DI water level did not reach the stainless-steel part of the unit. A magnetic stir bar was added to the water tray and the tray was placed on a magnetic stir plate at a medium speed, allowing water to slowly move around. Water was changed every 24 hours for three consecutive days. On the final day, the Pacing electrodes were dried at 70°C for 15 minutes, the hinges were screwed back, and autoclaved in separate bags. The autoclaved Pacing electrodes were stored in a sealable plastic container at room temperature. Pacing electrodes were stored wet during and after running an experiment until they were ready to be cleaned and sterilized.

The Pacing electrode set up was modified for assaying the monolayer. The metal hinge was exchanged with a spade connector (Extech, Nashua, NH, USA, Extech TL809 Electronic Test Lead Kit, TL809), allowing the plates to fit with Pacing electrodes inside the plate chamber of a SI8000 Cell Motion Imaging System (Sony Biotechnology, San Jose, CA, USA) that was used to record the contractility of the monolayers. See **Method Figure 7** for a detailed view of the set up.

The pacing unit hardware was also modified for assaying the dynamics of intracellular calcium transients. A reduction of plate height that contained the Pacing electrodes sitting on top of the EHT rack was required to fit the plate inside the plate chamber of the microscope for experiments. A lager spacer was used with 7.25-7.3 mm length and 2.2 mm diameter (Digi-key Electronics, Thief River Falls, MN, USA, 732-12686-ND) between the two steel bars of the Pacing electrodes, and while screwing the Pacing electrodes inside facing each other with a longer screw of 13.5 to 14.5 mm length (Digi-key Electronics, M2x0.4) which allowed the pacing unit to be fit inside the Silicon rack rather than on the outside. See **Method Figure 8** for a detailed view of the set up.

Tip: No detergents were used at any point during cleaning and sterilization. Occasionally, the metal bar developed an opaque layer, and a metal brush was used to carefully rub the thin layer off before cleaning. A non-lint wipe was used to wipe off the top of the metal bars.

5. OPERATION AND SETTINGS FOR DIFFERENT EXPERIMENTAL ASSAYS

Electrical pacing source

When relevant to experiments, cells in EHTs or in monolayers were electrically paced at controllable pacing rates and data was acquired with image-based assays as described in the following sections to quantify functional parameters related to contractility and dynamics of intracellular calcium transients. Depending on the instrument used for image acquisition, two different types of stimulators were used.

HSE Stimulator C Type 224

This electrical stimulator was used to pace the tissues and monolayers of cells when using the EHT measuring system or the SI8000 cell motion imaging system. To operate the stimulator, the stimulator was first connected to the power outlet and then connected the TTL sync output cable from the stimulator to the EHT BNC connector. Next, a banana jack from the stimulator was connected to the EHT instrument simply by following the matching colors of the outlet and the jack (red to red and black to black). A similar connection was established between the stimulator and the arrays of Pacing electrodes when electrically pacing cell monolayers. The parameters that were set prior to using the stimulator are listed in **Method Table 3**. Adjustments were made using the "var" key to switch between the variable or gradual adjustment mode of the parameter settings. The "SET" key was used to initiate or end the parameter setting and to increase the value in "SET" mode, the "<<" key was pressed to increase and ">>" key to decrease the value in "SET" mode. To abort settings or functions, both "<< and >>" were pressed together.

MyoPacer (IonOptix)

A MyoPacer was used to electrically pace EHTs when being imaged with an Axio Observer 7 inverted microscope (Carl Zeiss Microscopy, White Plains, NY, USA). A digital display allowed users to set voltage, pulse duration, and frequency. The MyoPacer was connected to the stimulation Pacing electrodes via two 2 mm connecting pins. Clipping wires were used to connect the pins to customized Pacing electrodes for imaging. Parameters were set by interacting with the LCD screen and an encoder knob that allowed for navigation through the different menus. The encoder had two modes of operation: turning, clicking, and by pushing. Turning by scrolling through the menus, the parameters of choice were selected with a click, and changed by turning the encoder again. The desired value was stored by another click. For experiments with the EHTs using this microscope, the

MyoPacer was set to a 4-millisecond duration bipolar waveform and 5-volt amplitude. The parameters set for the experiment are listed in **Method Table 3**.

Assaying the contractility in EHT and monolayers

The contractility of the EHT and monolayers was analyzed with image-based approaches consisting of acquiring fast videos (>60 fps) of beating tissues (12) or cells (10) and computationally analyzing these videos to quantify variations in force or cellular movement during each contractile cycle.

EHT measuring system

The video contractility assay required operation of the thermic mass flow controller and the EHT measuring system. In addition to measuring the drug-induced effect on EHT contractility, the contractility assay was routinely performed once every week to monitor the force development of each tissue. Prior to acquiring videos of beating tissues with the EHT measuring system, the Thermic mass flow controller was turned on to establish incubation settings of 7% CO2 and 40% O2 in a chamber that was also maintained at 37°C to image EHTs. The EHT measuring system was then powered up following vendor instructions and the software, CMTV Physio 12.0, was opened on the connected computer. The plate with EHTs was placed into the incubating chamber of the EHT measuring system once its environmental conditions were stable. Once the plate was settled in the chamber, the acquisition of contractile parameters was done via the software. Wells were selected while setting up the video acquisition to measure contractility, and then single clicked into the A2 position and clicked Camera view >Live > Start > Manual. Then the first well was clicked where the tissue was located. After selecting the well set to be analyzed first, the camera moved to that position, and then further utilized the X, Y and Z panels to adjust the position of the tissue. Upon adjusting and saving the position of each EHT per well, details on experimental conditions were added to the acquisition identification file and this process was repeated for each EHT being imaged. After that, the "peak force" parameters were set as recommended by the vendor:

- Peak force (double check the settings before recording)
- Number of frames for derivative calculation: 4
- Filter level: 10
- Baseline level: 0.95
- Force threshold: 0.02 – 0.05 mN
- Minimum factor: 0.2
- Maximum peak distance: 60s
- CV/RV: 0.2

After selecting the real time function, automatic acquisition of videos was initiated. After the acquisition and return of the plate to the incubator, data was saved and properly identified, and the EHT measuring system was turned off by closing all the gas regulators first, then the Thermic mass

flow controller until no more bubbling could be observed in the gas mixer wash flask. All the valves, the software, and finally the EHT system were closed to end the process.

SI8000 Cell Motion Imaging System

To use the motion detection system, its incubation chamber was first equilibrated to 37 °C and 5% CO_2 and the viewer software (SI8000 View software, Sony Biotechnology) installed in the computer controlling the system was opened. After setting acquisition of the monolayers in wells within the 24-well plate and placing the plate in the chamber with Pacing electrodes connected to the electrical stimulator, 10-second videos were acquired using the following software settings:

- Lens: PH
- Resolution: 2048x2048
- Magnification: 10x
- Frame per second: 75 fps
- Shutter speed: 1/75 sec
- Total frame: 800
- Duration: 10 s
- Auto focus and auto brightness boxes: unchecked
- Video and images options: checked

To capture an image or video, a location was first selected to save the acquired data. Separate folders for every column were created and then either video, or image, or both image and video were selected with a checkbox. Then, "Start" was clicked when everything was ready. A second HSE Stimulator C Type 224 was used for pacing cells in a monolayer. The operating procedure was the same as described earlier. The parameters were set at the same degree except the voltage. For the monolayers experiment a slightly higher voltage was used. The settings are listed in **Method Table 3**. Three locations in each well were selected for video recordings and the files were saved by columns.

Assaying dynamics of intracellular calcium transients

An Axio Observer 7 inverted microscope with heated stage, temperature, and humidity-controlled 37°C chamber was used in these experiments. Parameters were optimized and set before drug exposure. Video acquisitions were done with Zen 2.6 Blue software at Brightfield (BF) and Enhanced Green Fluorescent Protein (EGFP): 2×2 binning, ROI size of 2048×1264 with Y-offset of 368 (size: 1024×632 pixels and pixel size: 13×13 μm) using 10x objective. BF videos were acquired at varying lamp-intensity (16-25%) adjusted to the thickness of the tissue with a 10-millisecond exposure time for 10 second duration. Calcium fluorescence videos were acquired in the EGFP channel (Excitation 488 nm, Emission 509 nm) with a 15-millisecond exposure time for 10-second duration. An experimental set-up was calibrated to a 24-well plate that was utilized for EHTs and a heated stage plate (PeCon) to preset approximate x, y, z-coordinates. For each drug concentration, x, y, z-

coordinates were adjusted to acquire the middle tissue region with a 10x objective. On the day of the experiment, the microscope was first turned on for the temperature and humidity-controlled chamber to reach a stable point. After the equilibration, the plate was taken to the microscope and set onto the heated stage inside the temperature and humidity control chamber. Microscope parameters were set as described before and tissues were paced at frequencies of 0.5 Hz and 1 Hz using a MyoPacer. BF (101 frames per second) and EGFP (67 frames per second) videos of EHTs with WTC-11 GCaMPf hPSC-cardiomyocytes were obtained in control and three subsequent concentrations for each compound to determine the effect of acute drug exposure on the dynamics of intracellular calcium transients.

Contractile parameters estimated from acquired videos

A summary of how the acquired videos of EHT and monolayers were analyzed with dedicated software is provided:

Analysis from EHT system: Customized software (CMTV GmbH) was used to determine contractile force based on the known mechanical properties of the Silicon racks. Contractile force, F, was calculated from the extent of Silicon rack deflection δ (µm), length of the post (L), radius of the post (R), and known elastic modulus (E) of the casting material of the Silicon rack Polydimethylsiloxane (PDMS) by the following formula: F=3πER4δ/4L3. Contraction and relaxation velocities were derived from the average maximal steepness of contraction or relaxation peak, respectively. Each value represented the mean of all measurable contraction peaks in the set recording period of each EHT (13). The output parameters of the EHT system are listed in Method Table 4.

SI8000 Cell Motion Imaging System output parameters: The block matching algorithm divides each frame of a video into macroblocks and compares luminance similarity from one frame to the next neighboring one of the captured videos. Cardio model analysis software utilized this principle to quantify contraction and relaxation deformation by calculating the displacement vectors of cellular movement. A sophisticated Motion Vector algorithm allowed for obtaining a beat profile, from which contraction and relaxation parameters were also calculated (14, 15). The parameters that can be obtained from cardio model analysis software are listed in **Method Table 5**.

Dynamics of intracellular calcium transients parameters: Videos of EHT-WTC-GCaMP6f were imported into MATLAB (MATLAB R2017a) for analysis. MATLAB was used to identify calcium fluorescent intensity and velocities, while subtracting background noise. First, exported videos from the microscope were uploaded to identify the number of frames, frame rate, and conversion to micron of each video. Then, for each fluorescent video, a region of interest was manually selected, as well as a reference region, and the subtraction of background noise from detected calcium intensity changes. The region of interest and reference regions were kept consistent throughout experimental tissues. As a result of selected regions, graphical representations of calcium intensity were obtained, and pacing rate was detected based on a Fast Fourier Transform, rate of calcium signal velocities, and tissue contraction time equivalent for each video. Output values report calculated signal intensity, as well as curves that correspond to

intensity and velocity changes over time that were then used to compare the effects of compounds on calcium transients. Output parameters from the analyzed videos that were acquired to capture the dynamics of intracellular calcium transients are listed in **Method Table 6**.

6. TESTING DRUG EFFECTS

Preparation of solutions with compounds

Unless indicated otherwise, drugs were purchased from Apexbio Technology, TX, USA already dissolved in DMSO (Sigma-Aldrich,) at a concentration of 10 mM. Depending on experimental goals (**Method Table 7**), EHTs were exposed to compounds in Tyrode's solution or medium of relevance. Vials with 10 mM of compound in DMSO were stored at -20°C and aliquots were made for one-time use. On the day of aliquot preparation, the solution was thawed in the refrigerator at 4°C for one hour and aliquots were prepared with a volume ranging from 30 to 100 µL and stored as indicated below for six to 12 months. Total concentration of DMSO in solution being exposed to cells was 0.1% for all conditions. Drugs were exposed to cells following specific experimental goals described in **Method Table 7**, using medium formulations and exposure times relevant to the goals of the different experiments as detailed in the previous section. In general, ivabradine was used to inhibit spontaneous contractions prior to exposing cells to drugs known to enhance contractility in cardiomyocytes (5). Drugs that have enhanced contractile effect on cardiomyocytes (isoproterenol (Millipore Sigma, MA, USA, 420355-100 mg), EMD57003 (Millipore Sigma, 530657-10 mg), omecamtiv mecarbil (A8349), Bay K8644 (Millipore Sigma, 196878-1 mg)) or reduced (verapamil (Millipore Sigma, V4629-1 g), ranolazine (Millipore Sigma, R6152), mavacamten (A8720), ryanodine (B5092)) were used in experiments focused on testing the reproducibility of their effects on EHTs. Compounds were added at varying concentrations that were observed to yield different levels of changes in contractility. Aspirin (A4013) was used as a negative control. The contractile response of monolayers (7, 8) of hPSC-cardiomyocytes was also assayed upon exposing them to varying concentrations of isoproterenol, verapamil and ranolazine. Varying concentrations of EMD57003, omecamtiv mecarbil, mavacamten and verapamil were also exposed to EHTWTC11s made with cardiomyocytes differentiated from WTC11-PSCs expressing GCaMP6f (16) to image intracellular calcium transients in addition to assaying contractility. The effects of these drugs on the contractility of EHTWTC11s were compared with their effects on EHTiCell2 to investigate how different these can be when using cardiomyocytes differentiated from different PSC lines (17). In addition to assaying the acute effects of contractility affecting drugs, drugs known to cause cardiotoxicity (9) (doxorubicin (Millipore Sigma, 504042), sunitinib (B1045), erlotinib (A3397), paclitaxel (A4393) were also exposed to EHTiCell2 to analyze their subacute effects.

Ivabradine hydrochloride: A 10 mM solution in DMSO was purchased and stored at -20°C. The solution was thawed in the refrigerator at 4°C to prepare 30 µL aliquots of stock solutions that were stored at -20°C for up to six months. Before using, aliquots were thawed and 30 µL drug solution was mixed in 2970 µL of water for injection (Sigma-Aldrich, A12873-01). 6 µL of this diluted solution was added to the medium or Tyrode's solution to obtain a final concentration of 300 nM in 2 mL per well.

Aspirin (acetylsalicylic acid): Solutions were diluted in Tyrode's solution on the day of exposure to 50 µL aliquots. Cells in monolayer and EHTiCell2 were exposed to 0, 0.1, 1 and 10 µM of aspirin.

Isoproterenol, hydrochloride: Following vendor instructions, isoproterenol was acquired in powder form and stored in a dry and dark place at room temperature. It was reconstituted in solution prior to experiments being performed. A stock solution of 100 mM was prepared by dissolving 100 mg in 4.73 mL of water for injection and mixing the solution thoroughly. Working dilutions were further prepared for the experiments and stored at -20°C: 1000, 100, 10, 1, 0.1 µM in water for injection. Then, 20 µL of each concentration was added to 2 mL of 0.6 mM Tyrode's solution to obtain final concentrations of 10, 1, 0.1, 0.01 and 0.001 µM for experiment with monolayer and EHTiCell2.

Verapamil hydrochloride: Following vendor instructions, it was acquired in powder form and stored in a dry and dark place at room temperature. A stock solution of 20 mM was prepared by dissolving 25 mg of verapamil in 2.543 mL of DMSO. The solution was mixed thoroughly and then 50 µL of aliquots of stock concentration were prepared in PCR tubes and stored at -20°C. From the stock concentration, dilutions of 1000, 300, 100, 30, 10, and 3 µM in water for injection were prepared. Then, 2 µL of each concentration was added to 2 mL of 0.6 mM Tyrode's solution to obtain final concentrations of 1, 0.3, 0.1, 0.03, 0.01 and 0.003 µM for exposure to monolayers and EHTiCell2. For experiments with EHTWTC11s, dilutions of 100, 30 and 10 µM were prepared to obtain final concentrations of 0.01, 0.03 and 0.1 µM.

Ranolazine dihydrochloride: Following vendor instructions, it was acquired in powder form and stored in a dry and dark place at room temperature. A stock solution of 50 mM was prepared by dissolving 100 mg in 4 mL of DMSO, which was then thoroughly mixed. Aliquots with 100 µL of stock solution were prepared in PCR tubes and stored at -80°C. From the stock concentration, dilutions of 5000, 3000, 1000, 300, and 100 µM in water for injection were prepared and 20 µL of each concentration was added to 2 mL of 0.6 mM Tyrode's solution to obtain final concentrations of 50, 30, 10, 3, and 1 µM to expose monolayers and EHTiCell2s.

EMD57003: Following vendor instructions, it was acquired in powder form and stored in a glass bottle at -20°C in the dark. When ready to use, 10 mg of powder was reconstituted in 2.35 mL DMSO to obtain a 10 mM stock solution. Stock solutions of 300 µL EMD 57003were aliquoted into PCR tubes and stored them at -20°C for up to six months. On the day of experiments, an aliquot was thawed and used to prepare working dilutions of 5000, 1000, 500, and 100 µM in water for injection, and 20 µL of each concentration was added to 2 mL of 0.6 mM Tyrode's solution to obtain final concentration of 50, 10, 5 and 1 µM for drug exposure in EHTiCell2. For experiments with EHTWTC11s, 5000, 1000 and 100 µM of EMD57003 were prepared to obtain final concentrations of 50, 10 and 1 µM for drug exposure.

Omecamtiv mecarbil: Stock solutions were already dissolved in 1 mL DMSO at a concentration of 10 mM and were stored at -20°C. When ready to use, the tube was thawed in the refrigerator at 4°C and 70 µL aliquots were prepared in PCR tubes. The compound was diluted on the day of experiments using one of the aliquots. Working dilutions of 1000, 300, 100, 30 and 10 µM in water for injections were prepared and 20 µL of each concentration was added to 2 mL of 0.6 mM Tyrode's solution to obtain final concentrations of 10, 3, 1, 0.3 and 0.1 µM. 0.1% DMSO was added to all the Tyrode's solution wells except for the column with 10 µM to keep the final DMSO concentration at 0.1% for experiments conducted with EHTiCell2. For experiments with EHTWTC11s, dilutions of 1000, 100 and 10 µM were prepared with this compound to obtain 10, 1 and 0.1 µM final concentrations for drug exposure.

MYK-461/ mavacamten: Stock solutions were already dissolved in one mL DMSO at 10 mM concentration and were diluted on the day of experiments from one of the aliquots. Working dilutions were prepared of 3000, 1000, 300, 100 and 30 µM in water for injection and 2 µL of each concentration were added to 2 mL of 0.6 mM Tyrode's solution to obtain final concentrations of 3, 1, 0.3, 0.1 and 0.03 µM for EHTiCell2 exposure experiments. For experiments with EHTWTC11s, dilutions of 3000, 300 and 30 µM of this compound were prepared to obtain final concentrations of 3, 0.3 and 0.03 µM.

Bay K8644: Following vendor instructions, it was acquired in powder form and stored in a glass bottle at 4°C in the dark. When ready for use, 1 mg of powder was reconstituted in 279 µL of DMSO to obtain a 10 mM stock solution. 10 µL of stock solutions were aliquoted into PCR tubes and stored at -20°C. On the day of the experiment, one aliquot of the stock solution was used to prepare working dilutions of 100, 50, 30, 10 and 1 µM in water for injection and 20 µL of each concentration was added to 2 mL of 0.6 mM Tyrode's solution to obtain final concentrations of 1, 0.5, 0.3, 0.1 and 0.01 µM for experiments with EHTiCell2.

Ryanodine: Following vendor instructions, it was acquired in powder form and stored at -20°C. When needed to use, 1 mg of powder was reconstituted in 200 µL of DMSO to obtain 10 mM stock solution and thoroughly mixed it to prepare 425 µL of working dilutions of 2000, 1000, 300, 30 and 10 µM in water for injection. Aliquots of 20 µL of solution were mixed in 2 mL of 0.6 mM Tyrode's solution to obtain final concentrations of 20, 10, 3, 0.3 and 0.1 µM for exposing to EHTiCell2.

Erlotinib: A 10 mM solution in DMSO was purchased and stored at -20°C upon arrival. When needed, 100 µL aliquots were prepared in PCR tubes, which were then stored at -80°C, and thawed and diluted on the day of experiments. Working dilutions of 1, 0.5, and 0.1 mM in water for injection were prepared and 15 µL of each concentration was added to 1.5 mL of EHT medium without serum to obtain 10, 5, and 1 µM final concentrations for drug exposure to EHTiCell2.

Sunitinib: A 10 mM solution in DMSO was purchased and stored at -20°C upon arrival. When ready for use, the tube was thawed in the refrigerator at 4°C and 100 µL aliquots were prepared in PCR

tubes, which were then thawed and diluted on the day of experiments. Working dilutions of 1000, 500, and 100 µM in water for injections were prepared and 15 µL of each concentration was added to 1.5 mL of EHT medium without serum to obtain final concentrations of 10, 5, and 1 µM for drug exposure in EHTiCell2.

Doxorubicin, hydrochloride: A 10 mM solution in DMSO was purchased and stored at -80°C upon arrival. When ready to use, the tube was thawed in the refrigerator and 100 µL aliquots were prepared in PCR tubes and stored at -80°C for up to six months, being thawed and diluted on the day of experiments. Working dilutions of 100, 50, and 12.5 µM in water for injections were prepared and 15 µL of each concentration were added to 1.5 mL of EHT medium without serum to obtain final concentrations of 1, 0.5, and 0.125 µM for drug exposure to EHTiCell2.

Paclitaxel: A solution of Paclitaxel in DMSO at 10 mM concentration was purchased and stored at -20°C upon arrival. When ready for use, the tube was thawed in the refrigerator at 4°C, and 100 µL aliquots were prepared in PCR tubes and stored at -20°C for up to six months. On the day of the exposure, working dilutions of 5000, 1000, and 100 µM in water for injections were prepared and 15 µL of each concentration was added to 1.5 mL of EHT medium without serum to obtain 1, 0.5, and 0.125 µM final concentrations for drug exposure in EHTiCell2. Data was not shown for this drug compound due to low replicates used in the experiments.

Exposure of EHTs to test compounds known to increase contractility

A detailed workflow of the contractility increasing drug exposure experiments is illustrated in **Method Figure 11**. Responses were collected for two pacing rates for those drugs that are known to enhance cardiac contractility. Preparation of aspirin (control), isoproterenol, EMD57003, omecamtiv mecarbil and Bay k8644 were described above. Plates with Tyrode's solution and EHT medium were prepared for ivabradine exposure the day before the experiment. First, EHTs were incubated in ivabradine containing medium for one hour, then exposed to Tyrode's solution containing different concentrations of drug for acquiring contractility measurements, and finally tissues were returned to their original plates.

Plates with Tyrode's solution

Fresh or previously made 0.6 mM Tyrode's solution was prepared the day before the drug exposure. An appropriate amount of DMSO was added to prepare a 0.1% concentration in the Tyrode's solution, when DMSO was used to dissolve the selected drug compound. In a 24-well plate, two mL solution per well were added, excluding the first and last column in the plate due to poor visibility by the camera of the contractility measuring system. Prepared plates were incubated overnight. Two mL of 0.6 mM Tyrode's solution was added in column 6 to use it as a wash well for Pacing electrodes and tissues.

Plates with EHT medium for ivabradine

The day before the drug exposure, EHT medium was also prepared for the appropriate number of wells and pipetted them into a 24 well plate of two mL volumes. The first and last columns were excluded from plates, and prepared plates were incubated overnight. **Method Figure 15** represents an example of plate layout for such experiments.

Test compound incubation protocol

On the day of the drug exposure, the Thermic mass flow controller and EHT measuring system was turned on as described previously. Ivabradine was thawed and working solution was prepared as described. Ivabradine was added to all the wells containing the EHT medium or Tyrode's solution and mixed well. Plates were then placed back into the incubator. Then, baseline measurements of the tissues were recorded when the environmental conditions of the EHT measuring system was ready. The plate was taken to the biosafety cabinet to transfer tissues into EHT medium containing ivabradine for one hour. During this one-hour incubation, the drugs used for the given experiment were prepared. The appropriate dilutions were prepared, and the drugs were added to the equilibrated Tyrode's solution and placed back into the incubator. After an hour of incubation, quality control two that was described earlier was performed. If the tissues followed all the pacing frequencies, experimentation was continued. After performing the quality control, tissues were placed back into the biosafety cabinet along with the plates containing different dilutions of drugs in Tyrode's solution. The tissue rack was carefully transferred to the column containing the control (Tyrode's solution with DMSO) and incubated in the incubator for 15 minutes. In this step, the Pacing electrodes were kept on the same plate in column six, which contained 0.6 mM Tyrode's solution only. After 15 minutes of incubation, the tissue rack was held with one hand and the Pacing electrodes were placed in the well and the tissues were placed on top of the Pacing electrode. Then, tissue responses at 0.5 and 1 Hz pacing rates were recorded. In the CTMV software, the following naming convention for sample ID and Group ID was used for each condition:

- Sample ID: frequency Hz (0.5 and 1 Hz)
- Group ID: drug concentration

The concentration was gradually increased by moving the tissue rack from column 0 to the column containing the highest drug dose. Before exposing the tissue to the new concentration, the drug solution was quickly mixed and then the Pacing electrode and the tissues were transferred. Folder numbers associated with each concentration response were documented for future data processing. After recording all the responses, tissues were washed in the Tyrode's solution that contains 0.6 mM Calcium for 2 to 3 minutes and then transferred back to its original plate containing EHT medium.

Tip: The medium of the EHTs was changes the day prior to performing any drug studies. Usually, a Tuesday or Thursday worked well for performing drug exposure studies. Tissues were also given a full one-week recovery time before a second drug exposure experiment was performed on the same tissue.

Exposure of EHTs to test compounds known to decrease contractility

Effects were assayed with only one pacing rate with drugs that are known to decrease contractility. Preparations of verapamil, mavacamten, ranolazine, and ryanodine were described in the previous section. The preparation for contractility reducing drug exposure is similar to contractility increasing drug exposure. The two major differences: tissues were not exposed to ivabradine, which required elimination of quality control two, and tissues were only exposed to a 1.25 Hz pacing rate. Medium for the tissue was changed the day before the drug exposure, and 0.6 mM Tyrode's solution with 0.1% DMSO was prepared and equilibrated as described earlier. A step-by-step workflow for the contractility decreasing drug exposure experiment is illustrated in **Method Figure 12**.

Test compounds exposure to assay variation in dynamics of intracellular calcium transients in EHT

Dynamics of intracellular calcium transients analyses of EHTWTC11 were performed between days 26-65. EHTWTC11s were exposed to EMD 57033, omecamtiv mecarbil, verapamil, and mavacamten to test for variations in intracellular calcium transients. For this assay, a Zeiss microscope, MyoPacer and MATLAB were used to process the data. The process for setting up the microscope, MyoPacer, and the data analysis were described earlier. For EMD 57033 and omecamtiv mecarbil drug preparations, the same process was followed as described for EHT contractility enhancing drug exposure, while for verapamil and mavacamten, the process was as described for contractility reducing the drug exposure process. Videos were captured with a Zeiss microscope, exerted electrical pacing with a Myopacer, and processed data with MATLAB.

Compound exposure with cell monolayers

Experiments were performed to investigate the sensitivity of contractile responses of monolayer and EHT to aspirin (control), isoproterenol, verapamil, and ranolazine.

Incubation of cell monolayers with ivabradine

The aforementioned process was used to prepare Ivabradine. Monolayers were incubated with 300 nM of ivabradine for one hour before exposure to aspirin and isoproterenol. The ivabradine solution was added to wells containing EHT medium and all the wells with Tyrode's solution that were previously equilibrated. The EHT medium was then replaced in 24-well plate containing monolayers with medium that has ivabradine and incubated for one hour. Then, a baseline measurement of three representative wells were taken to ensure a reduced spontaneous beating of the monolayer.

Isoproterenol and aspirin exposure in monolayer

Isoproterenol and aspirin were prepared as previously described and aliquots were used on the day of the experiment. Isoproterenol was prepared on the same day of the exposure. The dilutions of drugs were added to the Tyrode's solution containing plate before the exposure. After a baseline

measurement of the monolayer following ivabradine incubation, both plates (monolayer and equilibrated Tyrode's solution with ivabradine), and the selected drug were taken into the biosafety cabinet. The drug solution was added to the first column of Tyrode's solution, Tyrode's solution was mixed (three times), the medium was aspirated from the monolayer, the Tyrode's+Drug solution was transferred to the monolayer plate, and then the Pacing electrodes were placed on top. Then, the lid was placed back on, the plate was taken to the SI8000 cell motion imaging system and placed into the plate holder. The glass was slid back on the top and left semi-open as the system did not close fully due to the hinge of the Pacing electrodes. The stimulator was turned on, the settings were checked, and the Pacing electrodes were connected with clipping wires and the previously saved plate layout was loaded. Then, Z was checked for each location (could be done for the entire column at a time) and update was clicked to save changes. After that, the following information was entered into the software:

- Memo 1: Compound name
- Memo 2: Pacing rate
- Memo 3: Dose

Then, measurements were taken with a pacing rate of one Hz and videos were saved in separate folders for each column. After the measurements, the monolayer was left in Tyrode's solution, medium was aspirated from the next column, Tyrode's solution with drugs was added, and then Pacing electrodes were transferred from the previous column to this column, and the next measurements were taken. After the recording, the plate was placed back into the biosafety cabinet, where all the Tyrode's solution was aspirated and replenished with fresh equilibrated EHT medium without aprotinin.

Verapamil and ranolazine drug exposure to monolayers

The day before the experiment, Tyrode's solution with 0.6 mM Calcium was prepared. The solutions were pipetted into two 24 well plates (two mL of solution per well) in the middle three columns and incubated overnight. 0.1% DMSO was only added to Tyrode's solution with 0.6 mM Calcium in columns that would have a low dose of the drug that did not contain a high percentage of DMSO (0.1%). On the day of the experiment, EHT medium without aprotinin was prepared and equilibrated in the medium change plate. Then, baseline contractile motion measurement of the monolayer was taken. Verapamil and ranolazine were prepared as described previously. The same process was followed for drug exposure and cell motion recording, except with a pacing rate of 1.25 Hz. At the end of the recording, cells were replenished with equilibrated fresh EHT medium without aprotinin and placed back in the incubator.

Cardiotoxic drug exposure in EHT

Experiments were designed to investigate sub-acute or delayed response and recovery in contractility due to cardiotoxic drug exposure. The preparation of erlotinib (control), sunitinib and doxorubicin was described earlier. Spontaneous contractility was recorded before, during and after the drug exposure.

The different stages for exposing and recording contractile effect of cardiotoxic drug are illustrated in **Method Figure 13.** There were several stages of the experiment. In the first stage, tissues were generated and maintained for 21 days. Contractility measurements were taken on day 14 and again on day 21. By day 21, tissues usually achieved an average force of 0.2 mN. If not, tissues were left incubating for an additional week. In the second stage, tissues were maintained at 4% serum medium once the tissues satisfied the first quality control (stable force of about 0.2 mN). The composition of 4% serum EHT medium was mentioned earlier. Tissues were kept in 4% serum EHT medium for 5 days and medium was changed every day. The third stage of this experiment was maintaining the tissues in serum-free EHT medium for 24 hours and then exposing them to selected drug compounds in serum-free EHT medium every 24 hours for 2 days. At the last stage of this experiment, tissues were maintained in 4% serum EHT medium for another 5 days while changing medium every day 24 hours after the second exposure of drug. Five days after drug exposure, the tissues were washed in a pre-equilibrated 0.6 mM Tyrode solution and stored for future gene expression experiments or discarded.

7. DATA ANALYSIS

Data was compared to control using paired t-test for the group data. Ordinary one-way ANOVA with Dunnett's multiple comparison test was performed for mixed model (Figure 1F, **Method Table 8**). Ordinary two-way ANOVA with Dunnett's multiple comparison test, with individual variances were used for mixed models (Figure 2 to 6). Data were presented as means ± SEM. Figure 1-3, represents the raw numbers, where value in figure 4-6 was expressed as a percentage of the control value. The level of statistical significance was expressed as p-values < 0.033 compared to control. **Method Table 8** lists all the statistical models used in the graphs presented in the original manuscript.

8. DETAILED METHODS AND MATERIALS REFERENCES

9.

10.

11.

1. **Lian X, Hsiao C, Wilson G, Zhu K, Hazeltine LB, Azarin SM, Raval KK, Zhang J, Kamp TJ, and Palecek SP**. Robust cardiomyocyte differentiation from human pluripotent stem cells via temporal modulation of canonical Wnt signaling. *Proc Natl Acad Sci U S A* 109: E1848-1857, 2012.

2. **Tohyama S, Hattori F, Sano M, Hishiki T, Nagahata Y, Matsuura T, Hashimoto H, Suzuki T, Yamashita H, Satoh Y, Egashira T, Seki T, Muraoka N, Yamakawa H, Ohgino Y, Tanaka T, Yoichi M, Yuasa S, Murata M, Suematsu M, and Fukuda K**. Distinct metabolic flow enables large-scale purification of mouse and human pluripotent stem cell-derived cardiomyocytes. *Cell Stem Cell* 12: 127-137, 2013.

3. **Mannhardt I, Eder A, Dumotier B, Prondzynski M, Kramer E, Traebert M, Sohren KD, Flenner F, Stathopoulou K, Lemoine MD, Carrier L, Christ T, Eschenhagen T, and Hansen A**. Blinded Contractility Analysis in hiPSC-Cardiomyocytes in Engineered Heart Tissue Format: Comparison With Human Atrial Trabeculae. *Toxicol Sci* 158: 164-175, 2017.

4. **Vollert I, Seiffert M, Bachmair J, Sander M, Eder A, Conradi L, Vogelsang A, Schulze T, Uebeler J, Holnthoner W, Redl H, Reichenspurner H, Hansen A, and Eschenhagen T**. In vitro perfusion of engineered heart tissue through endothelialized channels. *Tissue Eng Part A* 20: 854-863, 2014.

5. **Mannhardt I, Breckwoldt K, Letuffe-Brenière D, Schaaf S, Schulz H, Neuber C, Benzin A, Werner T, Eder A, Schulze T, Klampe B, Christ T, Hirt MN, Huebner N, Moretti A, Eschenhagen T, and Hansen A**. Human Engineered Heart Tissue: Analysis of Contractile Force. *Stem cell reports* 7: 29-42, 2016.

6. **Arefin A, Huang J, Platts D, Hypes V, Harris J, Iyer R, and Nath P**. Fabrication of flexible thin polyurethane membrane for tissue engineering applications. *Biomedical microdevices* 19: 1-9, 2017.

7. **Guth BD, Engwall M, Eldridge S, Foley CM, Guo L, Gintant G, Koerner J, Parish ST, Pierson JB, Ribeiro AJS, Zabka T, Chaudhary KW, Kanda Y, and Berridge B**. Considerations for an In Vitro, Cell-Based Testing Platform for Detection of Adverse Drug-Induced Inotropic Effects in Early Drug Development. Part 1: General Considerations for Development of Novel Testing Platforms. 10: 2019.

8. **Ribeiro AJS, Guth BD, Engwall M, Eldridge S, Foley CM, Guo L, Gintant G, Koerner J, Parish ST, Pierson JB, Brock M, Chaudhary KW, Kanda Y, and Berridge B**. Considerations for an In Vitro, Cell-Based Testing Platform for Detection of Drug-Induced Inotropic Effects in Early Drug Development. Part 2: Designing and Fabricating Microsystems for Assaying Cardiac Contractility With Physiological Relevance Using Human iPSC-Cardiomyocytes. *Frontiers in Pharmacology* 10: 2019.

9. **Mamoshina P, Rodriguez B, and Bueno-Orovio A**. Toward a broader view of mechanisms of drug cardiotoxicity. *Cell Rep Med* 2: 100216, 2021.

10. **Kopljar I, De Bondt A, Vinken P, Teisman A, Damiano B, Goeminne N, Van den Wyngaert I, Gallacher DJ, and Lu HR**. Chronic drug-induced effects on contractile motion properties and cardiac biomarkers in human induced pluripotent stem cell-derived cardiomyocytes. *Br J Pharmacol* 174: 3766-3779, 2017.

11. **Chen T-W, Wardill TJ, Sun Y, Pulver SR, Renninger SL, Baohan A, Schreiter ER, Kerr RA, Orger MB, Jayaraman V, Looger LL, Svoboda K, and Kim DS**. Ultrasensitive fluorescent proteins for imaging neuronal activity. *Nature* 499: 295-300, 2013.

12. **Mannhardt I, Saleem U, Benzin A, Schulze T, Klampe B, Eschenhagen T, and Hansen A**. Automated Contraction Analysis of Human Engineered Heart Tissue for Cardiac Drug Safety Screening. *J Vis Exp* 55461, 2017.

13. **Schaaf S, Shibamiya A, Mewe M, Eder A, Stöhr A, Hirt MN, Rau T, Zimmermann W-H, Conradi L, Eschenhagen T, and Hansen A**. Human Engineered Heart Tissue as a Versatile Tool in Basic Research and Preclinical Toxicology. *PLOS ONE* 6: e26397, 2011.

14. Noninvasive Evaluation of Contractile Behavior of Cardiomyocyte Monolayers Based on Motion Vector Analysis. *Tissue Engineering Part C: Methods* 18: 21-32, 2012.

15. **Hayakawa T, Kunihiro T, Ando T, Kobayashi S, Matsui E, Yada H, Kanda Y, Kurokawa J, and Furukawa T**. Image-based evaluation of contraction–relaxation kinetics of human-induced pluripotent stem cell-derived cardiomyocytes: Correlation and complementarity with extracellular electrophysiology. *Journal of Molecular and Cellular Cardiology* 77: 178-191, 2014.

16. Automated Video-Based Analysis of Contractility and Calcium Flux in Human-Induced Pluripotent Stem Cell-Derived Cardiomyocytes Cultured over Different Spatial Scales. *Tissue Engineering Part C: Methods* 21: 467-479, 2015.

17. **Mannhardt I, Saleem U, Mosqueira D, Loos MF, Ulmer BM, Lemoine MD, Larsson C, Améen C, de Korte T, Vlaming MLH, Harris K, Clements P, Denning C, Hansen A, and Eschenhagen T**. Comparison of 10 Control hPSC Lines for Drug Screening in an Engineered Heart Tissue Format. *Stem cell reports* 15: 983-998, 2020.

12. METHOD FIGURES

METHOD Figure 1

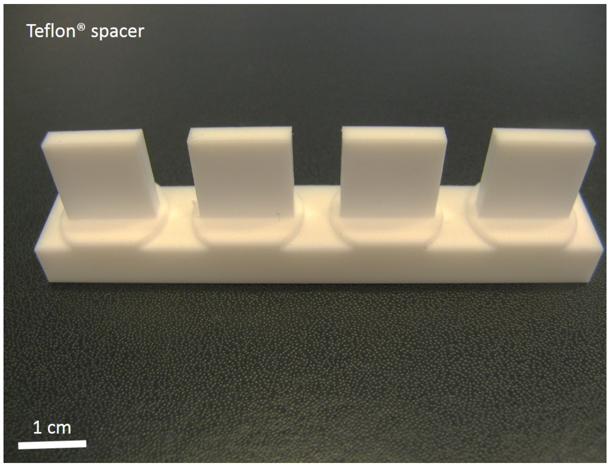

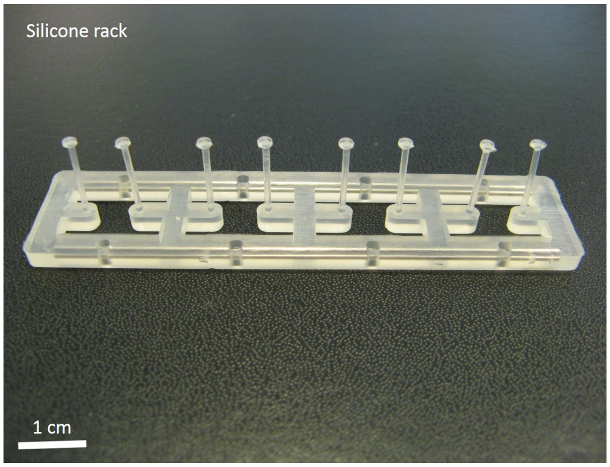

Method Figure 1: EHT fabrication components. The left image represents Teflon spacer and the right image represents the silicon rack used during EHT fabrication.

METHOD Figure 2

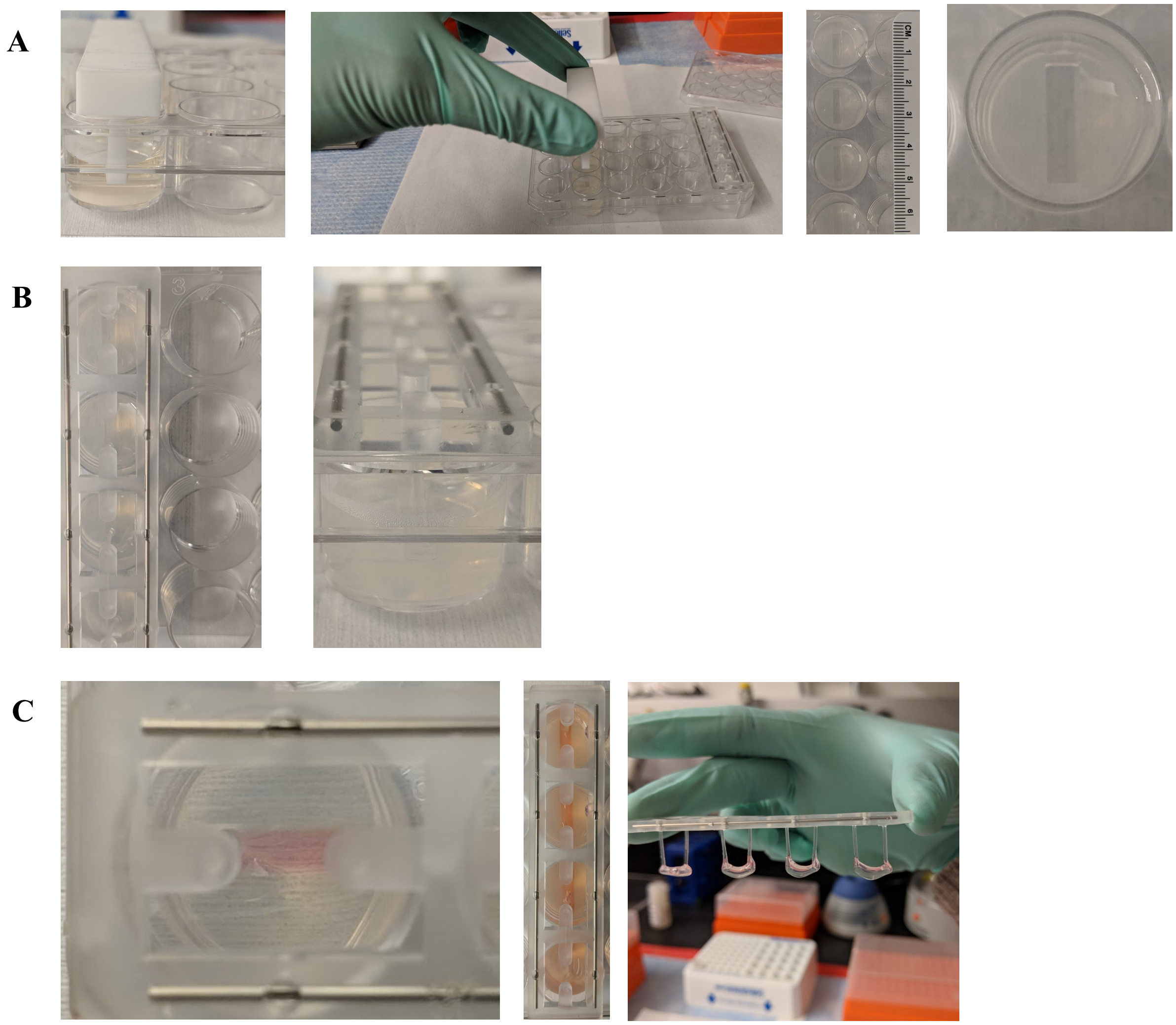

Method Figure 2: EHT fabrication steps. A) EHT construction mold was prepared with the aid of Teflon spaces and agarose. Once the mold was prepared silicon racks were placed inside the mold. Engineered heart tissue was fabricated inside the mold between the silicon posts.

METHOD Figure 3

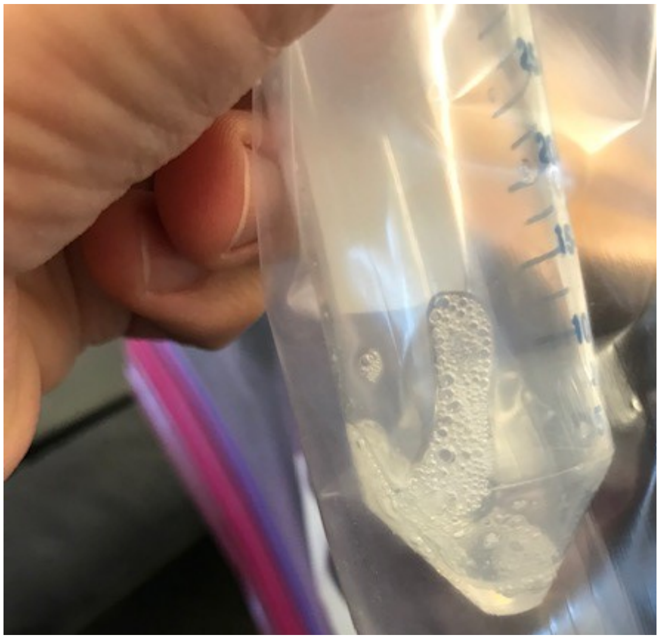

Method Figure 3: Troubleshooting fibrinogen preparation. Fibrinogen powder can form a clump leading to an unsuccessful preparation. The image represents one of the batches where fibrinogen solution was discarded where it did not fully dissolve within one hour of the preparation time and generated lots of bubbles during the process.

METHOD Figure 4

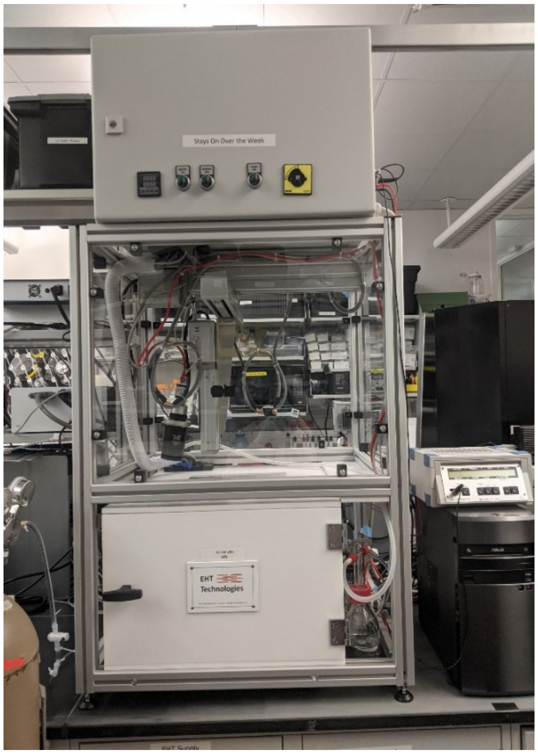

A) EHT measuring system

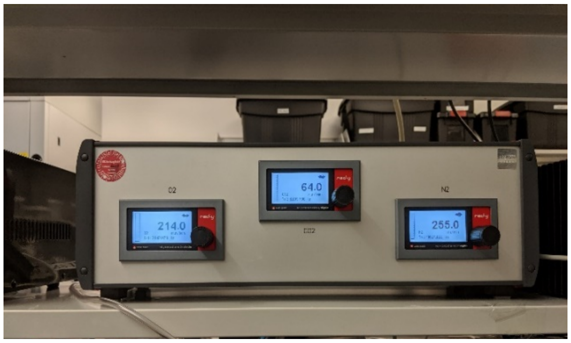

B) Thermic mass flow controller

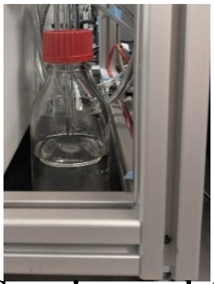

C) Gas mixer wash flask

Method Figure 4: Different components of EHT system. A) EHT measuring system consists of two parts: Upper part contains a camera for recording tissue contractility and the bottom part contains an environmentally controlled chamber where plates with tissue can be stored, B) an external gas flow controller is connected to a gas mixture flask, C) gas mixture flask is connected to the environmentally controlled chamber and provides necessary gas required for the chamber. The camera is controlled by custom software through a desktop computer connected to the EHT measuring system.

METHOD Figure 5

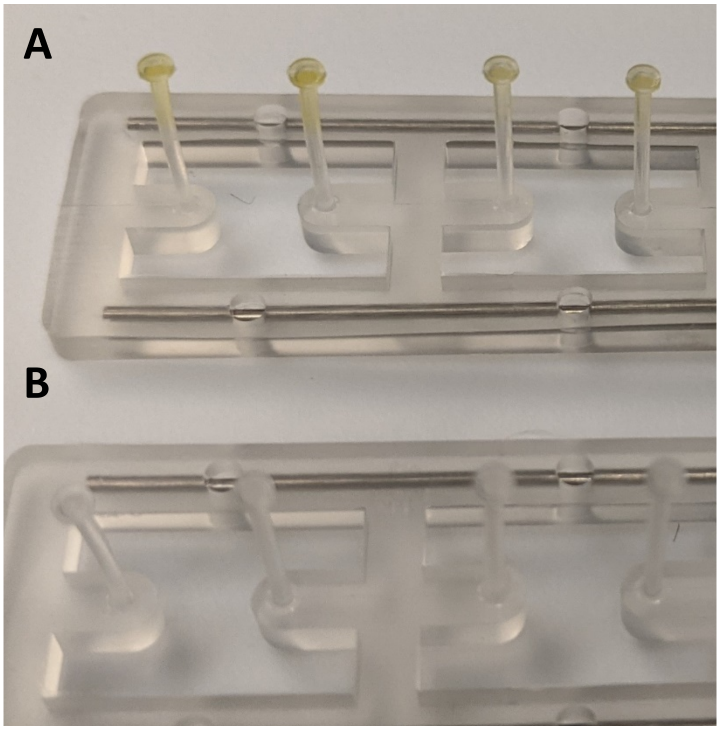

Method Figure 5: Long-term use of silicon racks. New silicon racks should be used for experiments designed to incubate tissue with drugs for more than 48 hours. A) Silicon rack turned yellow after used in a long-term experiment where tissue attached to the silicone posts were treated with 10 µM sunitinib for 48 hours, B) Silicon rack that used for tissue that were not treated with sunitinib.

METHOD Figure 6

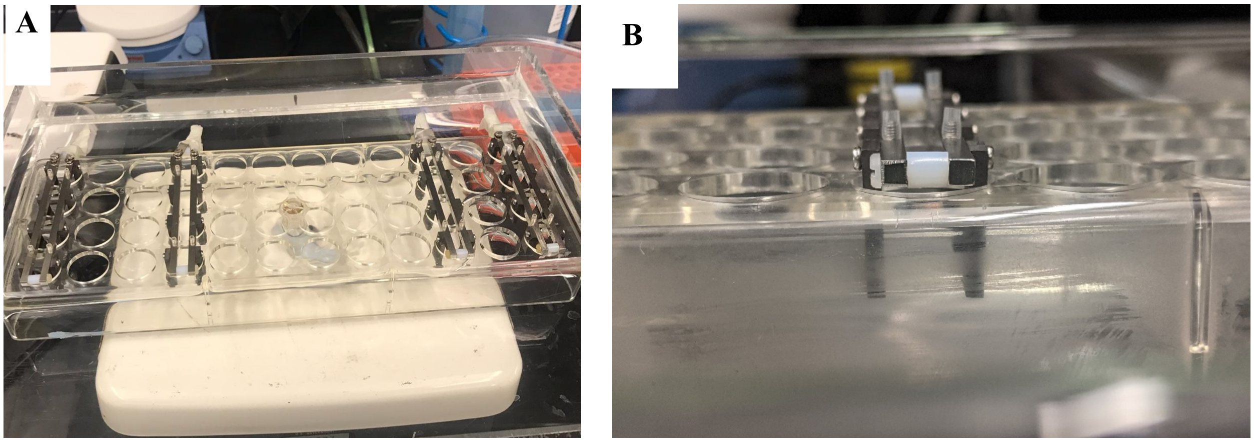

Method Figure 6: Electrode clean-up set up**.** A) Electrodes are submerged in DI water using a test tube rack. Magnetic stirrer was used to continuously move the water, B) Electrodes were submerged in a way so the top part of the electrode does not come in contact with water.

METHOD Figure 7

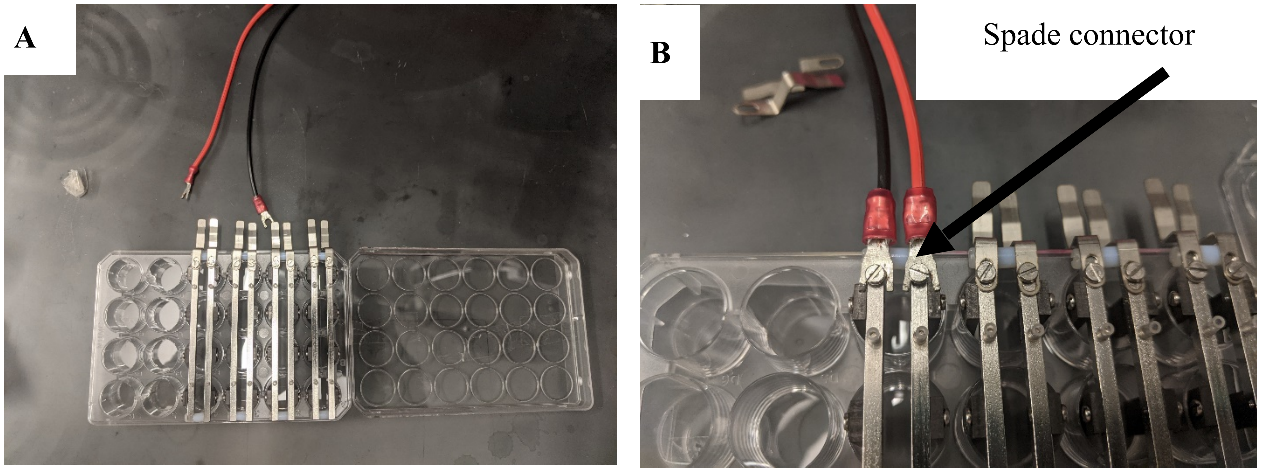

Original setup **Modified setup**

Method Figure 7: Electrodes were customized so monolayer can be paced using same experimental conditions as EHT**.** A) Original set up of the electrode to pace EHT, B) The metal hinged are replaced with spade connector to facilitate pacing of the monolayer using the same electrode and stimulator.

METHOD Figure 8

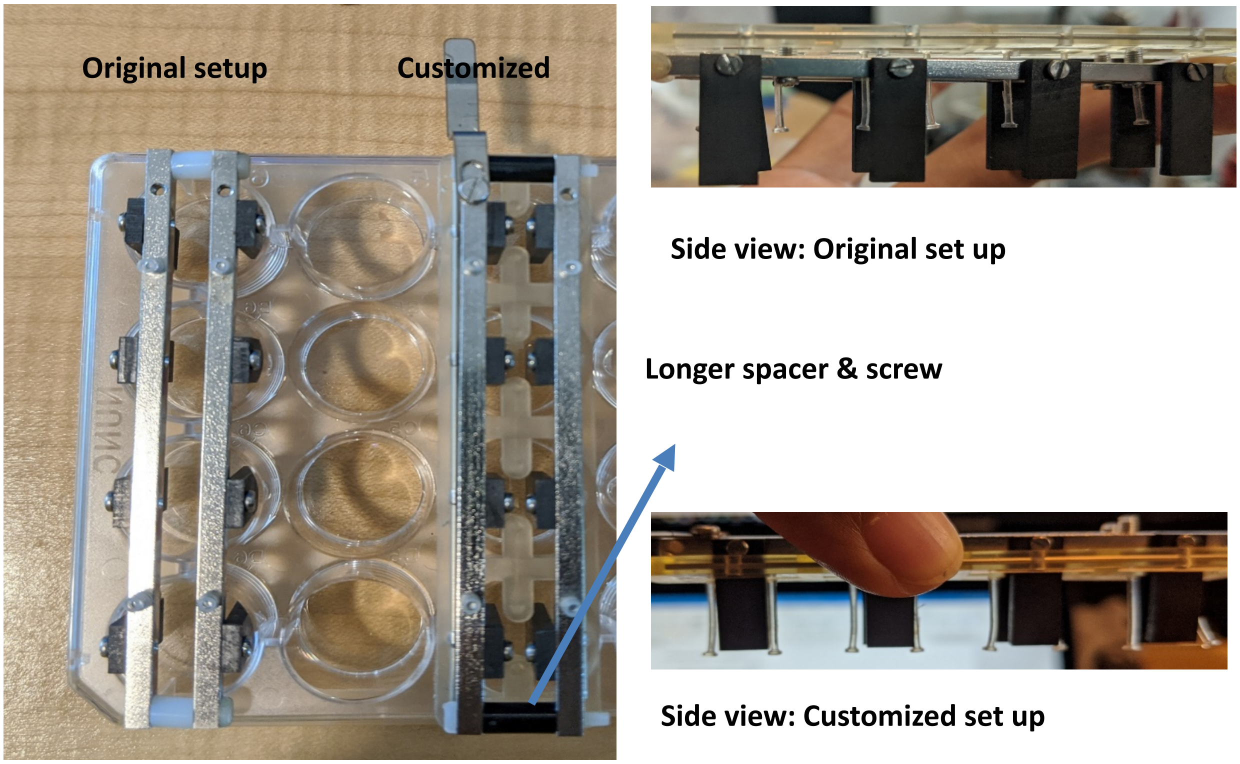

Method Figure 8: Electrodes were customized so EHTWTC11 generated from WTC11-GCaMPf-hPSC- cardiomyocytes can be paced when assaying the dynamics of intracellular calcium transients using a Zeiss microscope**.** In the original set up electrodes face outward, leaving space between two metal bars where the silicon rack can fit. But this setup creates an extra height that does not allow placement of the plate with electrodes and silicon rack together in the microscope stage. Customizing the spacing between the two-metal bar and fitting the silicon tack underneath the electrode allows for correct placement of the plate inside the microscope stage.

METHOD Figure 9

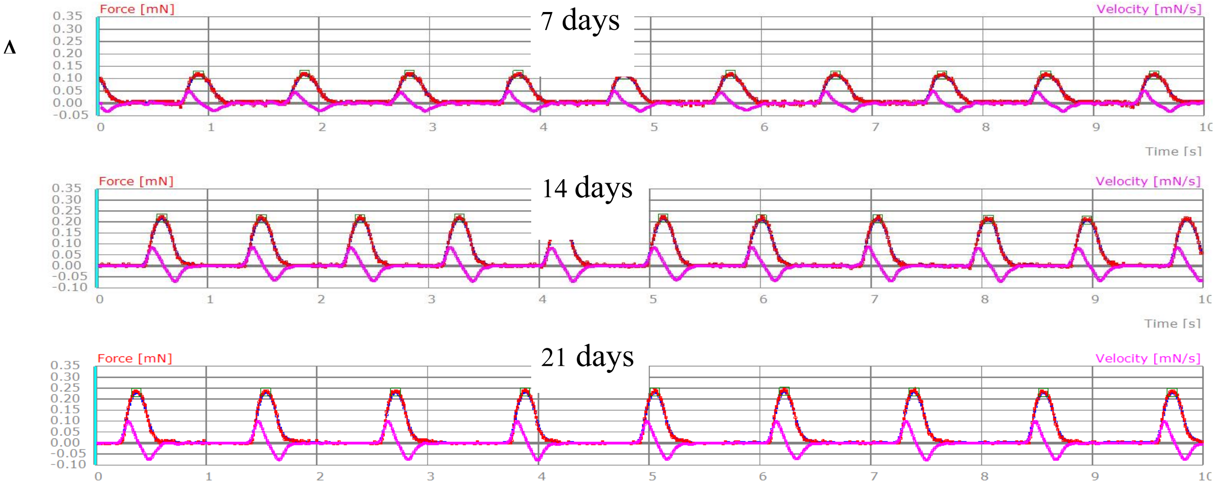

EHTiCell2: Quality control 1

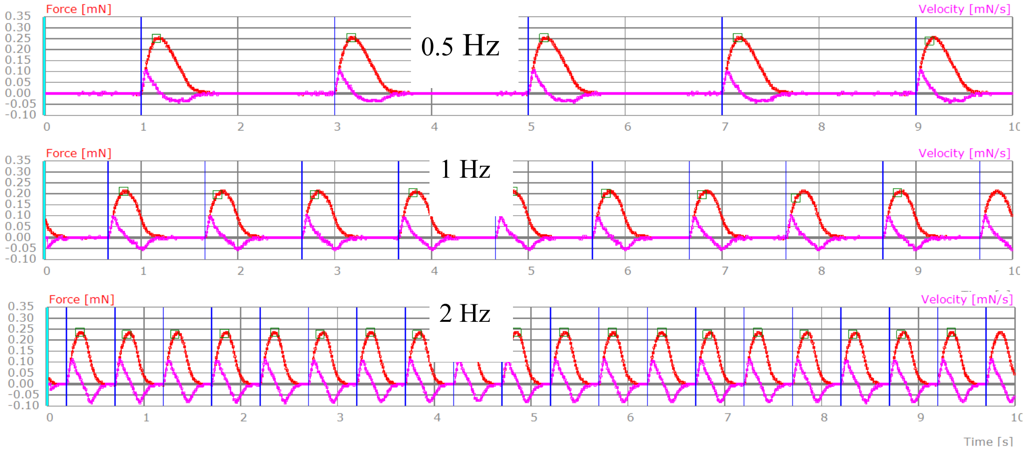

EHTiCell2: Quality control 2

Method Figure 9: Two main quality controls for assay reproducibility. A) Graph represents the contraction peak of EHTiCell2 generated from iCell cardiomyocyte2 recorded on day 7, 14, and 21. The X-axis represents force in mN, Y-axis represents time in seconds. An 80-120% of 0.2 mN force was considered as the first quality control for the tissue, B) Tissues were incubated with 300 nM of ivabradine for 1 hour in EHT media. Tissues were then exposed to three different pacing rates. Tissue following the exerted pacing rate passed the second quality control criteria.

METHOD Figure 10

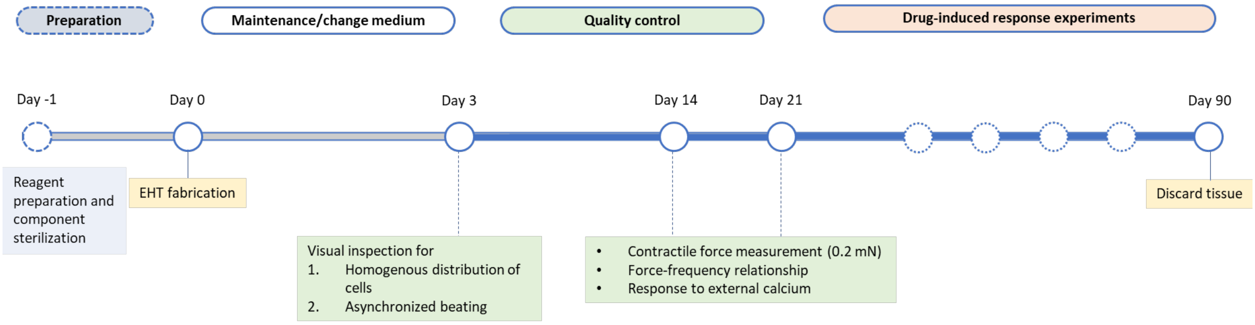

Method Figure 10: Workflow of the experiment for EHT characterization. Reagents and components of EHTs were sterilized and prepared the day before EHT fabrication. EHTs were fabricated following the vendor's instructions and optimized experimental conditions. On day 3, a visual inspection was performed to identify asynchronous beating. By day 14 and 21 tissues were monitored for two main quality controls. Response to external calcium was performed as an optional quality control measure. Once tissue successfully passed quality control criteria, experiments to obtain drug-induced responses were performed. Tissues that were passed quality controls can be maintained for up to 90 days with stable function. Tissues were discarded after the experiments.

METHOD Figure 11

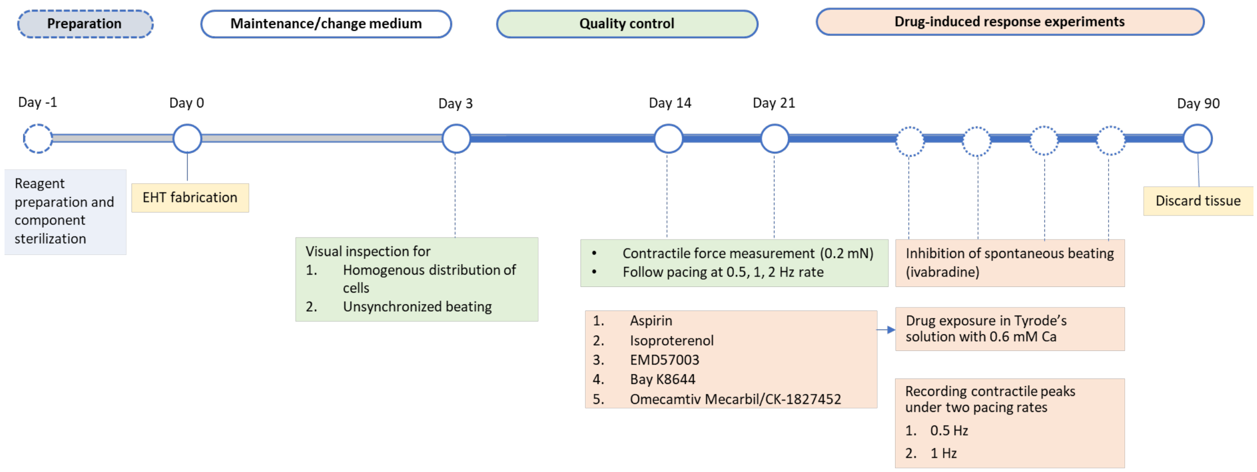

Method Figure 11: Workflow of the experiment investigating effect of contractility-increasing compounds in EHTs. Reagents and components of EHT were sterilized and prepared the day before EHT fabrication. EHTs were fabricated following the vendor's instructions and optimized experimental conditions. On day 3, a visual inspection was performed to identify asynchronous beating. By days 14 and 21 tissues were monitored for two main quality controls. Once tissue successfully passed quality control criteria, experiments to obtain drug-induced responses were performed. For contractility-increasing compounds, tissues were first incubated with 300 nM of ivabradine for one hour in EHT media to reduce the spontaneous beat rate. After 1 hour, tissues were exposed to aspirin, isoproterenol, EMD57033, bay K8644, and omecamtiv mecarbil at two different pacing rates (0.5 Hz and 1 Hz) in modified Tyrode's solution with 0.6 mM calcium. Drug-induced contractility responses were recorded using the EHT measuring system. The tissues were returned to their original plate after the experiment. Tissues were maintained for at least one week before a second drug exposure experiment was performed. Drug-exposure experiments were performed between day 21 and day 90 after fabrication.

METHOD Figure 12

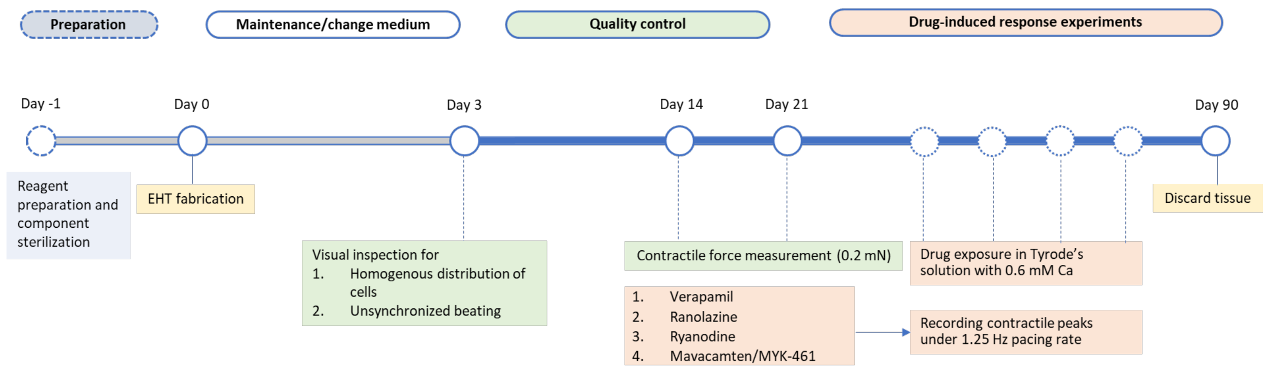

Method Figure 12: The workflow of the experiment investigating the effect of contractility-decreasing compounds in EHTs Reagents and components of EHT were sterilized and prepared the day before EHT fabrication. EHTs were fabricated following the vendor's instructions and optimized experimental conditions. On day 3, a visual inspection was performed to identify asynchronous beating. By days 14 and 21 tissues were monitored for two main quality controls. Once tissue successfully passed quality control criteria, experiments to obtain drug-induced responses were performed. For contractility-decreasing compounds, tissues were exposed to verapamil, ranolazine, ryanodine, and mavacamten at 1.25 Hz pacing rates in modified Tyrode's solution with 0.6 mM calcium. Drug-induced contractility responses were recorded using the EHT measuring system. The tissues were returned to their original plate after the experiment. Tissues were maintained for at least one week before a second drug exposure experiment was performed. Drug-exposure experiments were performed between day 21 and day 90 after fabrication.

METHOD Figure 13

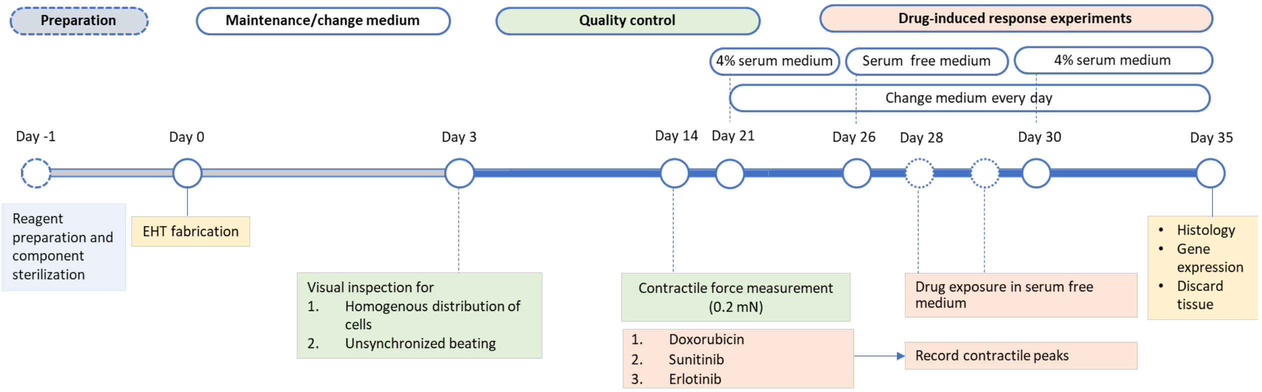

Method Figure 13: Workflow of the experiment investigating the long-term effect of cardiotoxic compounds in EHTs. Reagents and components of EHT were sterilized and prepared the day before EHT fabrication. EHTs were fabricated following the vendor's instructions and optimized experimental conditions. On day 3, a visual inspection was performed for identifying asynchronous beating. By days 14 and 21 tissues were monitored for only one quality control criterion. Once tissue successfully passed quality control criteria, tissues were adapted to a 4% serum medium for five days. Then tissues were maintained in serum-free media for 24 hours before exposing them to cardiotoxic drugs. For cardiotoxic compounds, tissues were exposed to doxorubicin, sunitinib, and erlotinib (control) for 48 hours in a serum-free EHT medium. Tissues were maintained in 4% serum media for another 5 days after drug withdrawal. Drug-induced contractility responses were recorded daily using EHT measuring system. Tissues were discarded after the end of the experiment.

METHOD Figure 14

	1	2	3	4	5	6
A						
B						
C						
D						

Method Figure 14: Plate layout for culturing monolayer. Monolayers generated from iCell cardiomyocyte2 were always seeded and cultured in columns 2, 3, and 4. This layout was optimized for pacing and recording videos of the monolayer using the SONY SI800 Cell Motion Imaging System.

METHOD Figure 15

	1	2	3	4	5	6
**A**	Media+Iva					Wash
**B**	Media+Iva					Wash
**C**	Media+Iva					Wash
**D**	Media+Iva					Wash

Method Figure 15: Plate layout for recording the drug-induced response of EHTs. Tissues are usually incubated with ivabradine in EHT media in column 1. Columns 2-5 are always dedicated to recording the response of EHTs to different concentrations of drugs. Column six is dedicated to keeping Tyrode's solution with 0.6 mM calcium for the in-between wash of the tissues during exposing the tissue from one concentration of drugs to a different concentration of drug solution.

13. METHOD TABLES

METHOD Table 1: The various types of media used throughout the study.

Media Type	Use
EHT casting media (NKM)	EHT formation
EHT media	EHT release from agarose and maintenance
EHT media without aprotinin	Maintenance of monolayers
EHT media with 4% serum	Transition media for chronic toxicity study
EHT media without any serum	Drug exposure media for chronic toxicity study
iCell Cardiomyocyte plating media	Thawing iCells during EHT formation and during thawing and plating iCells for monolayers
Thawing media for WTC11-GCaMP-iPSC-CMs	Thawing WTC11-GCaMP-iPSC-CMs during EHT formation

METHOD TABLE 2: Tyrode's solution formulation.

**Tyrode's solution custom (with ca++) (c-7650f)**	**Tyrode's solution custom (without ca++) (c-7651f)**
120 mM NaCl	120 mM NaCl
5.4 mM KCl	5.4 mM KCl
1 mM MgCl2	1 mM MgCl2
5 mM CaCl2	
0.4 mM NaH2PO4	0.4 mM NaH2PO4
22.6 mM NaHCO3	22.6 mM NaHCO3
5 mM glucose	5 mM glucose
0.05 mM Na2EDTA	0.05 mM Na2EDTA
25 mM HEPES	25 mM HEPES

METHOD Table 3: Electrical stimulator settings for various contractility measuring platform used in the study.

**EHT measuring system**	**SI8000 Cell Motion Imaging System**	**Axio Observer 7 inverted microscope**
**HSE Stimulator C Type 224**	**HSE Stimulator C Type 224**	**MyoPacer (IonOptix)**
Start mode: Free run	Start mode: Free run	
Frequency: 0.5 Hz to 2 Hz	Frequency: 0.5 to 1.25 Hz	Frequency: 0.5 to 1.3 Hz
Delay: 0.10 ms	Delay: 0.10 ms	
Width: 4 ms	Width: 4 ms	Pulse duration: 4 ms
Amplitude: 5 V/cm	Amplitude: 7 V/cm	Amplitude: 5 V/cm
Polarity: Biphasic	Polarity: Biphasic	Bipolar waveform
F-set: 1	F-set: 1	
Instrument set up: Normal run	Instrument set up: Normal run	

METHOD Table 4: Output parameters from EHT System.

Contraction parameters	Measurement
Frequency of contraction (bpm)	Number of contractions (beats) per minute
Force of contraction (mN)	Calculation of the post deflection, elastic properties, and geometry
TTP (-10%) (s)	Time to peak from 10% contraction
TTP (-20%) (s)	Time to peak from 20% contraction
TTP (-50%) (s)	Time to peak from 50% contraction
TTP (-80%) (s)	Time to peak from 80% contraction
TTP (-90%) (s)	Time to peak from 90% contraction
Relative TTP (early phase)	(TTP (-80%) - TTP (-50%))/TTP (-80%)
Relative TTP (late phase)	TTP (-50%)/TTP (-80%)
RT (10%) (s)	Time from peak to 10% relaxation
RT (20%) (s)	Time from peak to 20% relaxation
RT (50%) (s)	Time from peak to 50% relaxation
RT (80%) (s)	Time from peak to 80% relaxation
RT (90%) (s)	Time from peak to 90% relaxation
Relative RT (early phase)	(TTP (80%) - TTP (50%))/TTP (80%)
Relative RT (late phase)	TTP (50%)/TTP (80%)
Contraction velocity (mN/s)	Average maximal steepness of twitch contraction (df/dt)
Relaxation velocity (mN/s)	Average maximal steepness of twitch relaxation (df/dt)

Pacing to peak	Time from pacing signal to peak
RR Scatter (s)	Scatter of beat-to-beat variability
Resting length (mm)	Maximal distance between blue crosses, indicating length of the EHT

METHOD Table 5: Output parameters from SI8000 cell motion imaging system.

Contraction parameters	Measurement
Beating area	
Beating rate	Plotting the magnitude of motion vectors against time
Contraction velocity	Plotting the magnitude of motion vectors against time
Relaxation velocity	Plotting the magnitude of motion vectors against time
Contraction-end velocity	
Acceleration	
Contraction deformation distance	Total area under contraction peak
Relaxation deformation distance	Total area under relaxation peak
Noise level	
Contraction duration	
Relaxation duration	
Contraction-relaxation duration	Duration between the onset of the contraction peak and the offset of the relaxation peak defined as the contraction–relaxation duration
Contraction-relaxation peak interval	
Correlation	

METHOD Table 6: Output parameters from dynamics of intracellular calcium transients video acquisition.

**Contraction parameters**	**Measurement**
Intensity difference [-]	Subtraction of background noise from detected calcium intensity changes
F/F0 [-]	Change of calcium fluorescent intensity
Rate of Ca2+ signal rise [-/s]	Rate of intensity variation during contraction
Rate of Ca2+ signal extinction [-/s]	Rate of intensity variation during relaxation
Contraction time [s]	Time between the maximum rate of relaxation and maximum rate of contraction
Frequency (glob.) [Hz]	Fast fourier transform algorithm

METHOD Table 7: List of drugs, concentrations exposed to EHT/ cell monolayers, and medium/buffer used in the different experiments.

Experimental goals/ compound	Concentrations exposed to cells in culture	Used cell medium
**Inhibition of spontaneous contractions**		EHT medium
Ivabradine HCL	300 nM	
**Comparing the contractile effects of compounds between EHTs and monolayers of hiPSC-cardiomyocytes**		Tyrode's solution with 0.6 mM calcium
Aspirin	0, 0.1, 1 and 10 µM	
Isoproterenol, hydrochloride	0, 1, 3, 10, 30 and 100 nM	
Verapamil hydrochloride	0, 3, 10, 30, 100 and 300 nM	
Ranolazine dihydrochloride	0, 10, 30 and 50 µM	
**Effects of compounds known to increase/ decrease contractility in EHTs with different lines of iPSC-cardiomyocytes**		Tyrode's solution with 0.6 mM calcium
EMD57003	0, 1, 10 and 50 µM	
Omecamtiv mecarbil	0, 0.1, 1 and 10 µM	
MYK-461/ Mavacamten	0, 0.03, 0.3 and 3 µM	
Verapamil hydrochloride	0, 0.01, 0.03 and 0.1 µM	
**Effect of compounds known to increase/ decrease contractility in EHTs with iCell-cardiyomyocyte2**		Tyrode's solution with 0.6 mM calcium
Bay K8644	0.01, 0.1, 0.3, 0.5, and 1 µM	

Ryanodine	0.1, 0.3, 3, 10, and 20 μM	
**Assaying long-term drug effects**		EHT medium without any serum
Erlotinib	1, 5, and 10 μM	
Sunitinib	1, 5, and 10 μM	
Doxorubicin, hydrochloride	0.125, 0.5, and 1 μM	
Paclitaxel	1, 10, 50 and 100 μM	

METHOD table 8: Statistics details for the original data published in the main figures.

Figures	Replicates	Statistics	Post hoc	P value style
**Figure 1B**	EHTiCell2-A, n = 6, EHTWTC11-A, n = 14, EHTiCell2-B, n = 5, EHTWTC11-B, n = 8.	Ordinary one-way ANOVA		APA: 0.12(ns), 0.033(*), 0.002(**), <0.001(***)
**Figure 1C**	n=3	Paired t test, two-tailed		
**Figure 1E**	monolayers (n = 4), EHTs (n = 6)			
**Figure 1F**	(83, 70, 69) for EHTiCell2s, and n = (30, 30, 34) for EHTWTC11	Ordinary one-way ANOVA		
**Figure 2**	EMD57003 were N = 5 and n = 12 with EHTiCell2s, N = 3 and n = 11 with EHTWTC11s; for omecamtiv mecarbil were N=4 and n=16 with EHTiCell2s, N = 3 and n = 11 with EHTWTC11s		Sidak's multiple comparison test	
**Figure 3**	verapamil incubations: N = 4 and n = 18 with EHTiCell2s, N = 3 and n = 11 with EHTWTC11s. For mavacamten incubations: N = 5 and n = 11-25 with EHTiCell2s, N = 3 and n = 7-11 with EHTWTC11s			
**Figure 4**	isoproterenol N = 2 and n = (9-17) with monolayers, N = 4 and n = (20-27) with EHTs; for verapamil: N = 2 and n = (3-12) with monolayers, N = 4 and n = 18 with EHTs ; for ranolazine: N = 2 and n = (6-15) with monolayers, N = 3 and n = 6 with EHTs; for aspirin: N = 2 and n = 9 with monolayers, N = 2 and n = [illegible]	Ordinary two-way ANOVA		
**Figure 5**	N = 3 and n = 11 verapamil, N = 3 and n = 7-11 mavacamten, N = 3 and n = 11 EMD57003, N = 3 and n = 11 omecamtiv mecarbil			
**Figure 6**	N = 1 and n = 3 using doxorubicin, N = 1 and n = 3 using sunitinib, N = 1 and n = 3 using erlotinib	Ordinary one-way ANOVA	Dunnett's multiple comparison test (comparison between doses)<br><br>Tukey's multiple [illegible]	
