# Supplementary material for: Reproducibility of drug-induced effects on the contractility of an engineered heart tissue derived from human pluripotent stem cells
Source: Front Pharmacol. 2023 Jul 4;14:1212092. doi: 10.3389/fphar.2023.1212092 (PMC10352809; doi:10.3389/fphar.2023.1212092)
Supplement: Supplementary file 6 [file DataSheet1.pdf]

Supplementary Material

Reproducibility of drug-induced effects on the contractility of an engineered heart tissue derived from human pluripotent stem cells

Ayesha Arefin, Melissa Mendoza, Keri Dame, M. Iveth Garcia, David G. Strauss & Alexandre J.S. Ribeiro

*** Correspondence:**

Ayesha Arefin: rfnayesha@gmail.com

Alexandre J.S. Ribeiro: axribeiro3@gmail.com

Supplementary Figures

Supplementary Figure S1: Related to Figure 1.

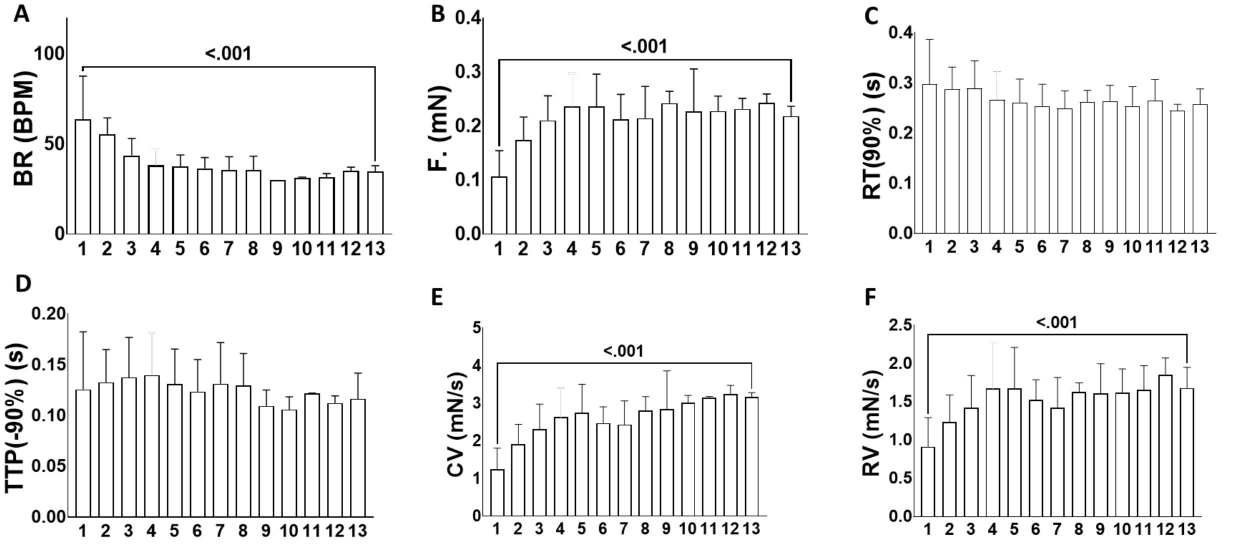

Supplementary Figure S1: Stability in contractile parameters by week in EHTiCell2. Contractile functions of EHTiCell2 generated from iCell cardiomyocyte2s were recorded in tissue maintenance media using EHT measuring system for 90 days. Data represents tissues from batch 4. Error bar indicates ±SD. Beat rate (BR), Force (F.), Time to peak from 90% relaxation (RT(90%)) , Time to peak from 90% contraction (TTP(-90%)) , Contraction velocity (CV), Relaxation velocity (RV), and Y-axis represents number of weeks.

Supplementary Figure S2: Related to Figure 1.

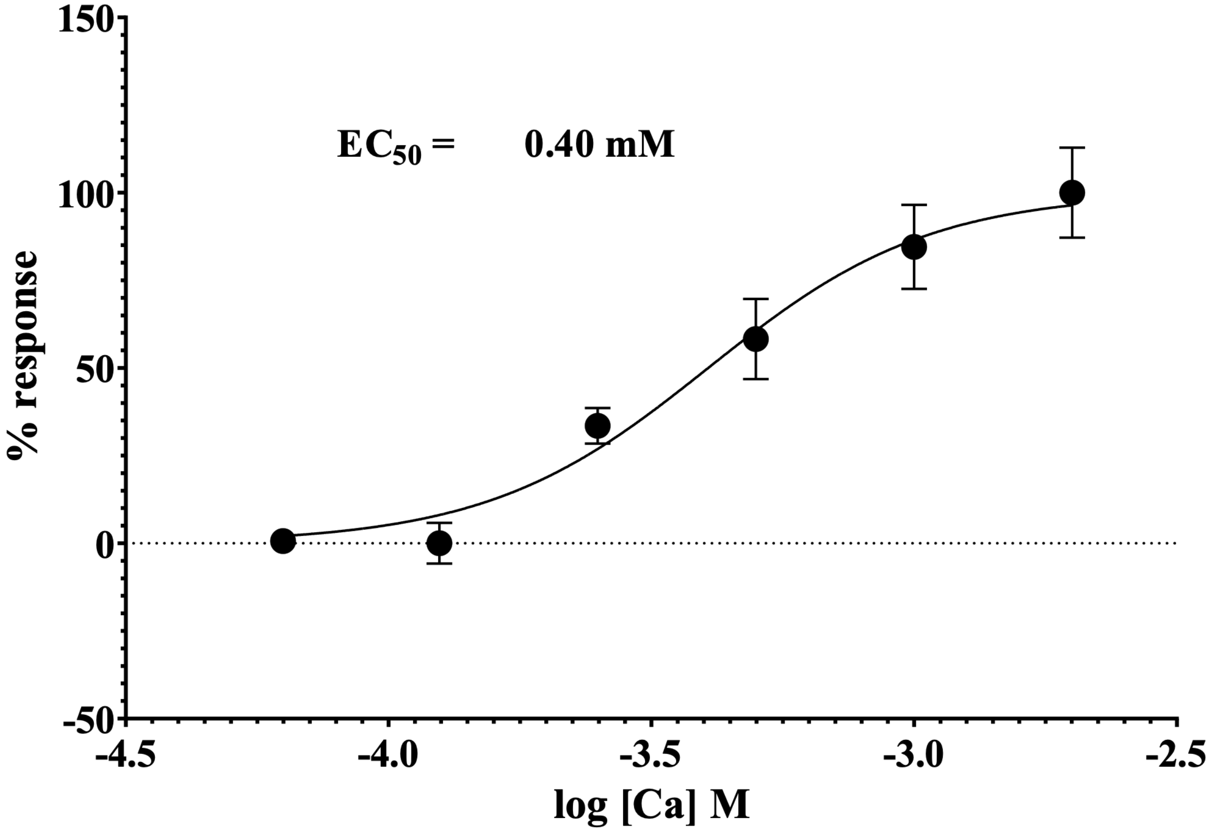

Supplementary Figure S2: Concentration response curve of external calcium in EHTiCell2. Concentration response curve of external calcium in EHTiCell2. EHTiCell2 responses to various extracellular calcium concentrations were recorded in Tyrode's solution at a 1.25 Hz pacing rate. Error bar indicates ±SD.

Supplementary Figure S3: Related to Figure 1.

Beat rate at 0.5 mM concentration

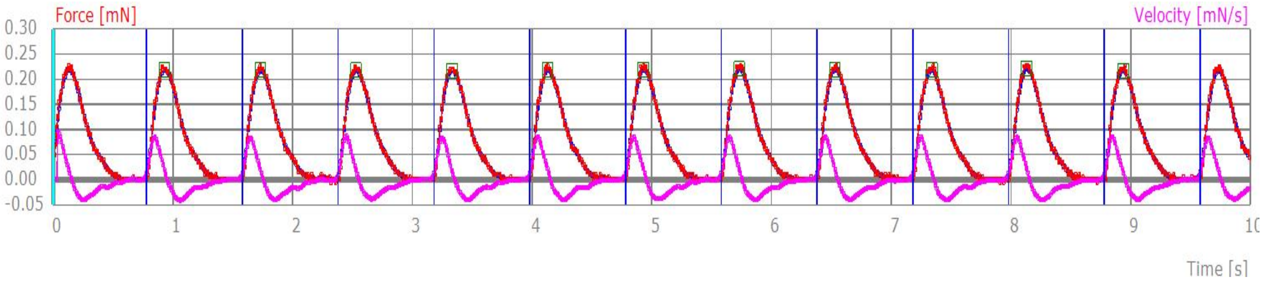

Unstable beat rate at 2 mM concentration

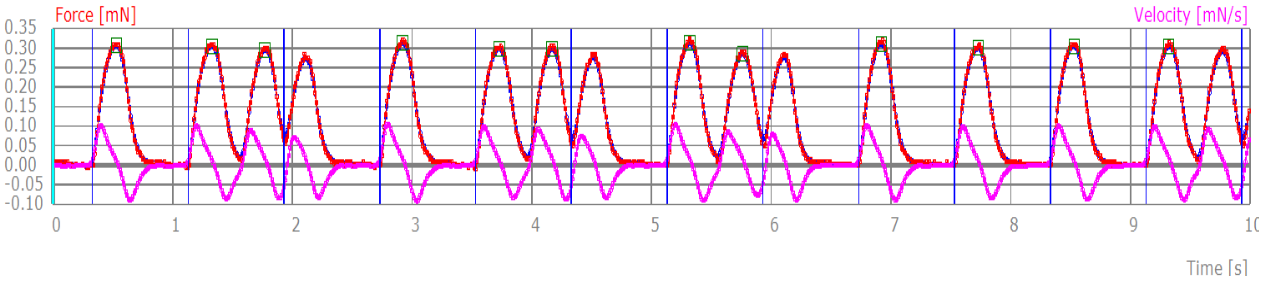

Supplementary Figure S3: Variation in beat rate with different concentrations of calcium exposure in EHT paced at 1.25 Hz. Graphs represent the contraction peak acquired by exposing EHTiCell2 to various extracellular calcium concentrations in Tyrode's solution at a 1.25 Hz pacing rate. Blue vertical lines represent the electrical pacing while red curves represent the contraction profile.

Supplementary Figure S4: Related to Figure 1.

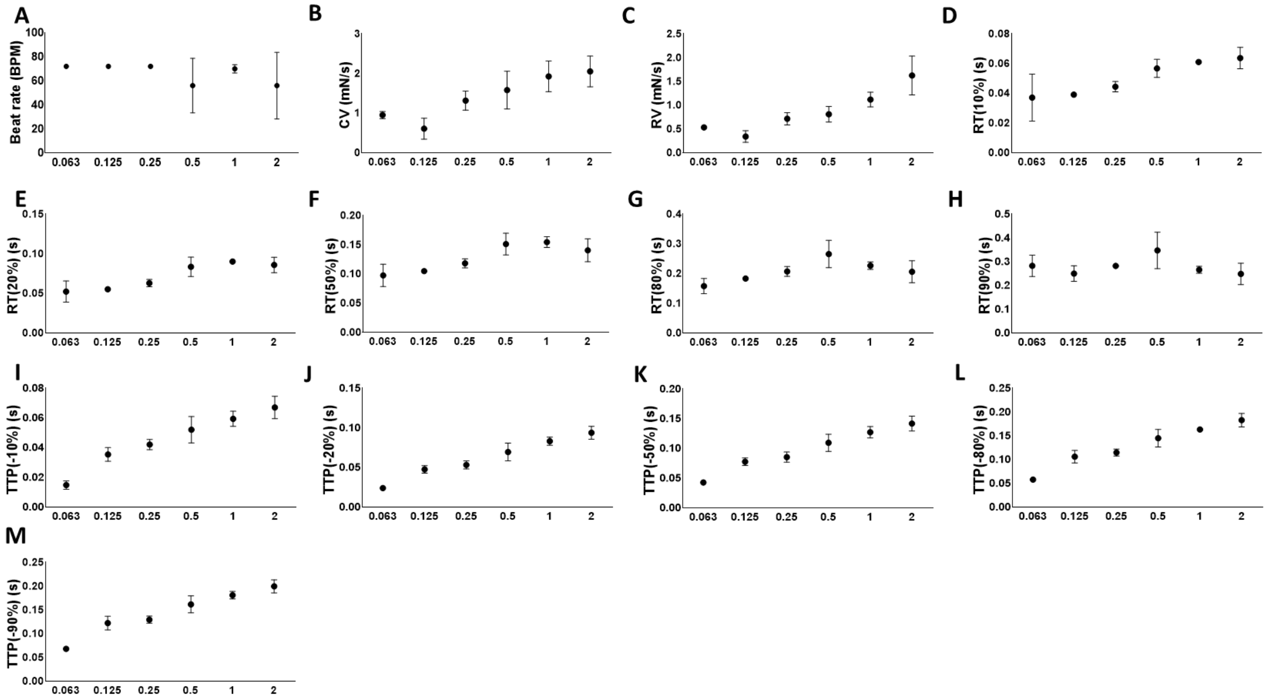

Supplementary Figure S4: Effect of extracellular calcium on different contractile parameters. Graphs represent variations in contractile parameters at different extracellular calcium concentrations. Responses were acquired in Tyrode's solution at a 1.25 Hz pacing rate. Y-axis represents calcium concentration at mM.

Supplementary Figure S5: Related to Figure 1.

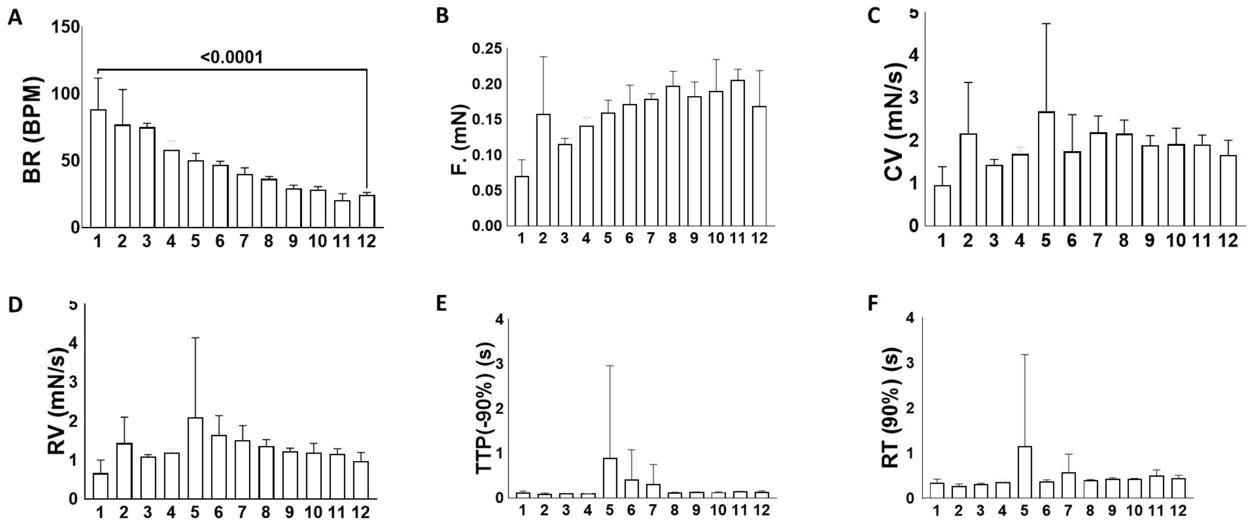

Supplementary Figure S5: Stability in contractile parameters by week in EHTWTC11. Contractile functions of EHTWTC11 generated from WTC11-GCaMPf-hPSC- cardiomyocytes were recorded in tissue maintenance media using EHT measuring system for 12 weeks. Data represents tissues from batch 3. Error bar indicates ±SD. BR = Beat rate, F. = Force, TTP(-90%) = time to peak from 90% contraction, RT(90%) = time to peak from 90% relaxation, CV = contraction velocity, RV = relaxation velocity. Y-axis represents number of weeks.

Supplementary Figure S6: Related to Figure 1.

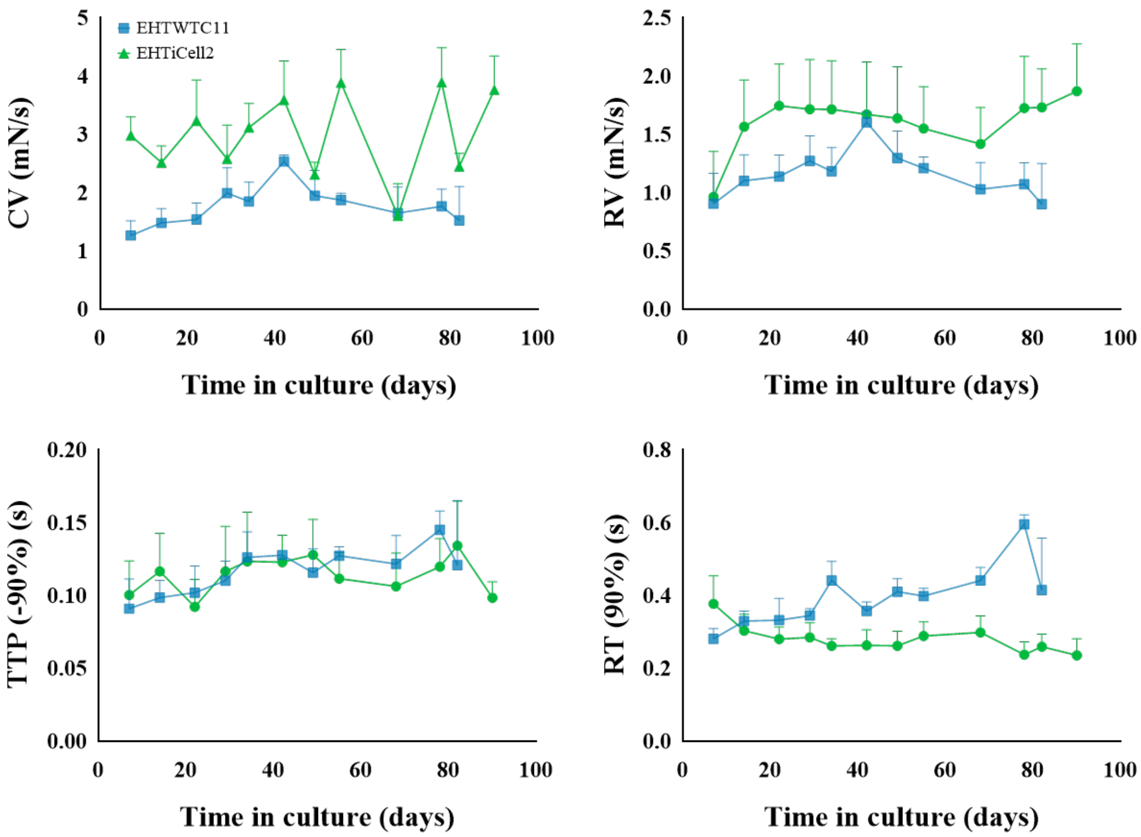

Supplementary Figure S6: Differences in contraction and relaxation kinetics between EHTWTC11 and EHTiCell2**.** Contractile functions of EHTs that were generated from WTC11-GCaMPf-hPSC-cardiomyocytes and iCell cardiomyocyte2s were recorded in tissue maintenance media using EHT measuring system for 90 days. Data represents tissues from batches that passed the quality control criteria. Error bar indicates ±SD. BR = Beat rate, F. = Force, TTP(-90%) = time to peak from 90% contraction, RT(90%) = time to peak from 90% relaxation, CV = contraction velocity, RV = relaxation velocity.

Supplementary Figure S7

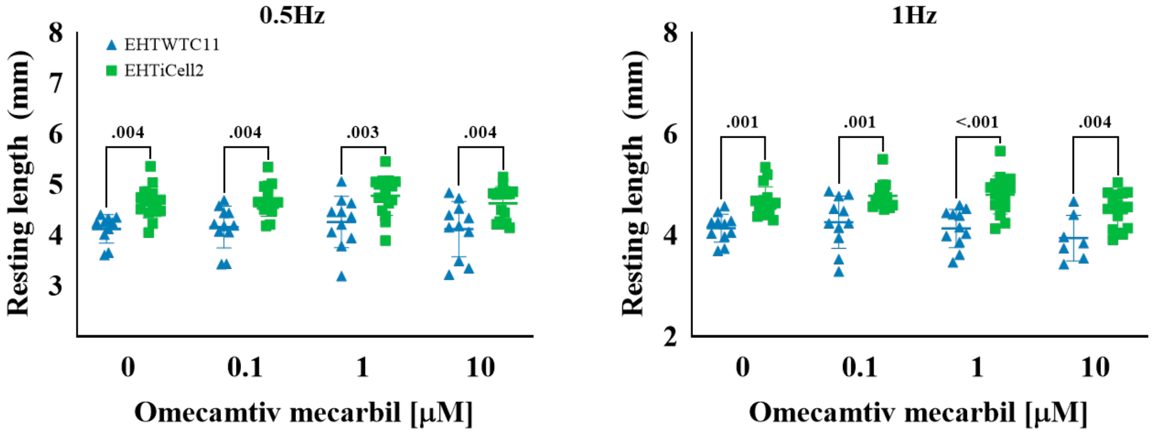

Supplementary Figure S7: Effects in EHTs of omecamtiv mecarbil on tissue resting length. Images show data collected from EHTs generated from iCell Cardiomyocytes2 (EHTiCell2) and from hPSC-cardiomyocytes expressing GCaMPf (EHTWTC11). Responses were acquired at 0.5 Hz and 1 Hz pacing rate in modified Tyrode's solution with 0.6 mM calcium. EHTs were pre-incubated for one hour in 300 nM of ivabradine. P-values were determined by ordinary two-way ANOVA using Sidak's multiple comparison test, with a single pool variance. Number of experiments (N) and replicates (n) for N=4 and n=16 with EHTiCell2s, N = 3 and n = 11 with EHTWTC11s.

Supplementary Figure S8: Related to Figure 2.

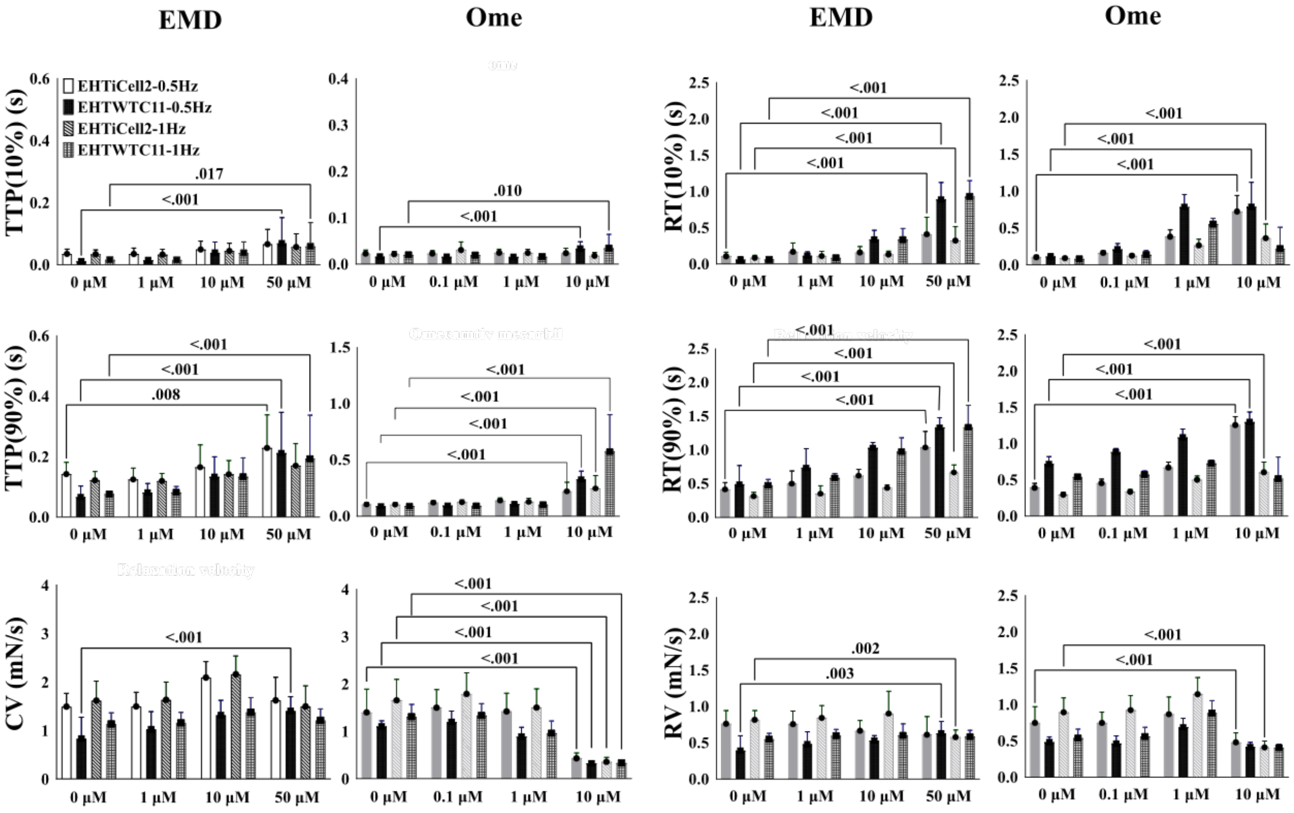

Supplementary Figure S8: Frequency-dependent effects of compounds known to increase contractility in EHTs. Effects of EMD57033 and omecamtiv mecarbil on contraction and relaxation time and speed were measured. Images show data collected from EHTs generated from iCell Cardiomyocytes2 (EHTiCell2) and from hPSC-cardiomyocytes expressing GCaMPf (EHTWTC11). Responses were acquired at 0.5- 1 Hz pacing rate in modified Tyrode's solution with 0.6 mM calcium. EHTs were pre-incubated for one hour in 300 nM of ivabradine. Data represents mean ± SD, P-values were determined by two-way ANOVA using Dunnett's multiple comparison test. Number of experiments (N) and replicates (n) for EMD57003 were N = 5 and n = 12 with EHTiCell2s, N = 3 and n = 11 with EHTWTC11s; for omecamtiv mecarbil were N=4 and n=16 with EHTiCell2s, N = 3 and n = 11 with EHTWTC11s. EMD = EMD57003, Ome = omecamtive mecarbil, TTP(-10%) = time to peak from 10% contraction, TTP(-90%) = time to peak from 90% contraction, CV = contraction velocity, RT(10%) = time to peak from 10% relaxation, RT(90%) = time to peak from 90% relaxation, RV = relaxation velocity.

Supplementary Figure S9: Related to Figure 3.

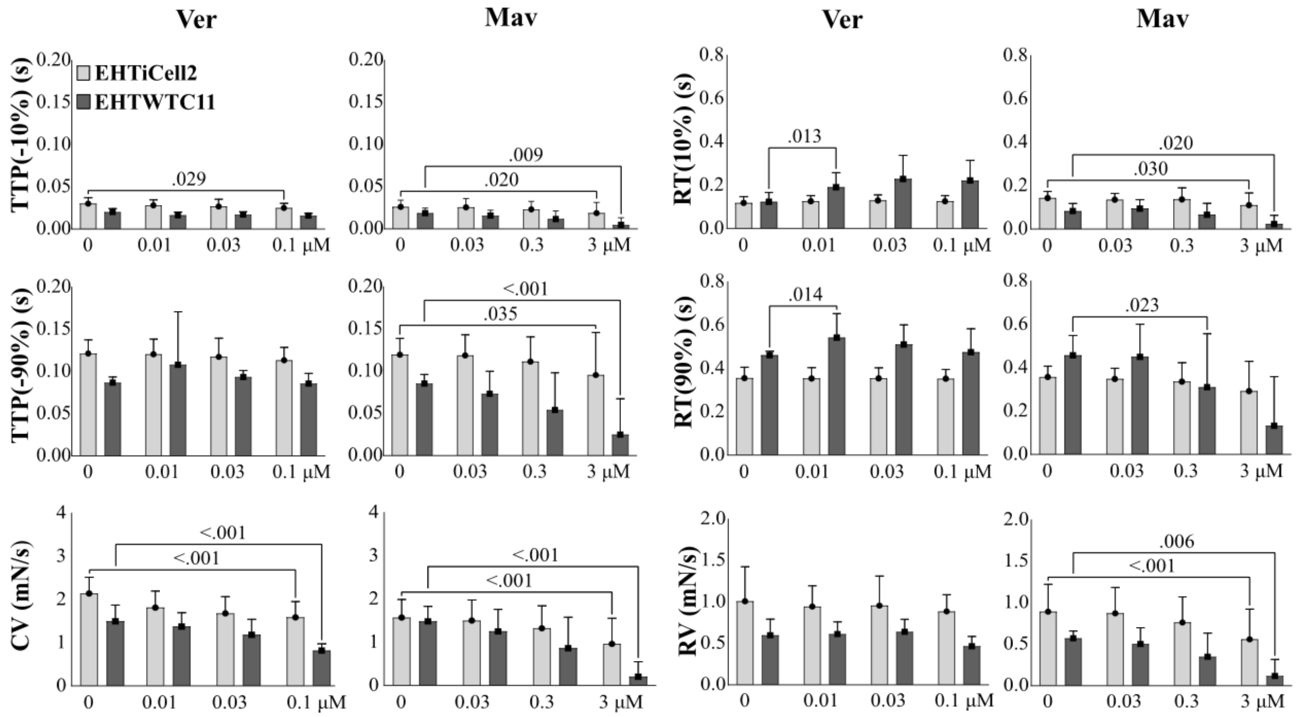

Supplementary Figure S9: Effects in EHTs of compounds known to decrease contractility. Effects of verapamil and mavacamten on contraction and relaxation time and speed were measured. Images represent drug-induced responses acquired at 1.25 Hz pacing rate in modified Tyrode's solution with 0.6 mM calcium using EHTs generated from iCell cardiomyocytes2 (EHTiCell2), and EHTs generated from hPSC-cardiomyocytes expressing GCaMPf (EHTWTC11). Data represent mean ± SD, P-values were determined by two-way ANOVA using Dunnett's multiple comparison test. Number of experiments (N) and replicates (n) for verapamil incubations: N = 4 and n = 18 with EHTiCell2s, N = 3 and n = 11 with EHTWTC11s. For mavacamten incubations: N = 5 and n = 11-25 with EHTiCell2s, N = 3 and n = 7-11 with EHTWTC11s. Ver = verapamil, Mav = mavacamten, TTP(-10%) = time to peak from 10% contraction, TTP(-90%) = time to peak from 90% contraction, CV = contraction velocity, RT(10%) = time to peak from 10% relaxation, RT(90%) = time to peak from 90% relaxation, RV = relaxation velocity.

Supplementary Figure S10: Related to Figure 3.

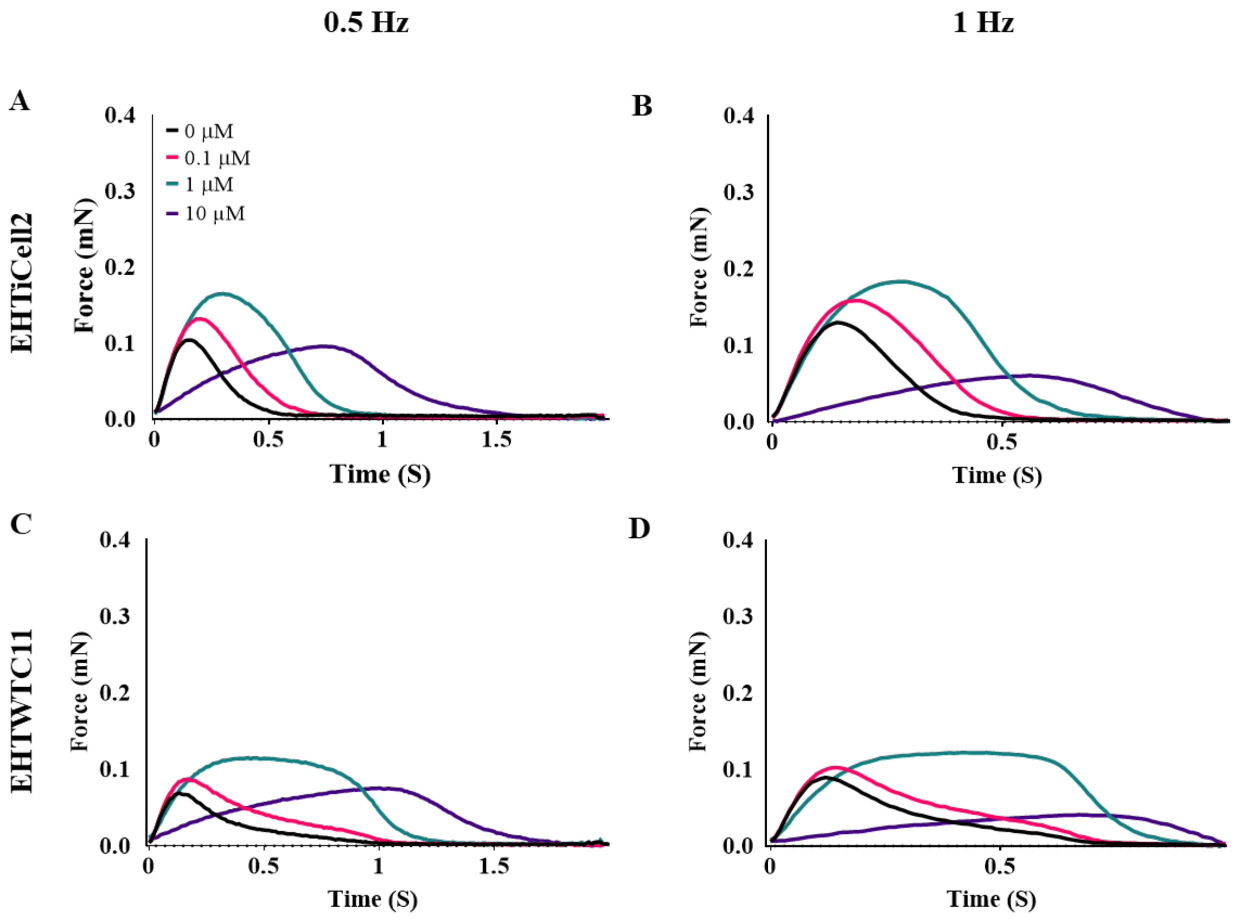

Supplementary Figure S10: Divergent changes in contraction peak at higher concentration in EHT in response to omecamtiv mecarbil. Graphs show average contraction peaks recorded when EHTiCell2 generated from iCell cardiomyocyte2 and EHTWTC11 generated from WTC11-GCaMPf-hPSC- cardiomyocytes were exposed to omecamtiv mecarbil in modified Tyrode's solution with 0.6 mM calcium at 0.5 and 1 Hz pacing rate.

Supplementary Figure S11: Mentioned in discussion.

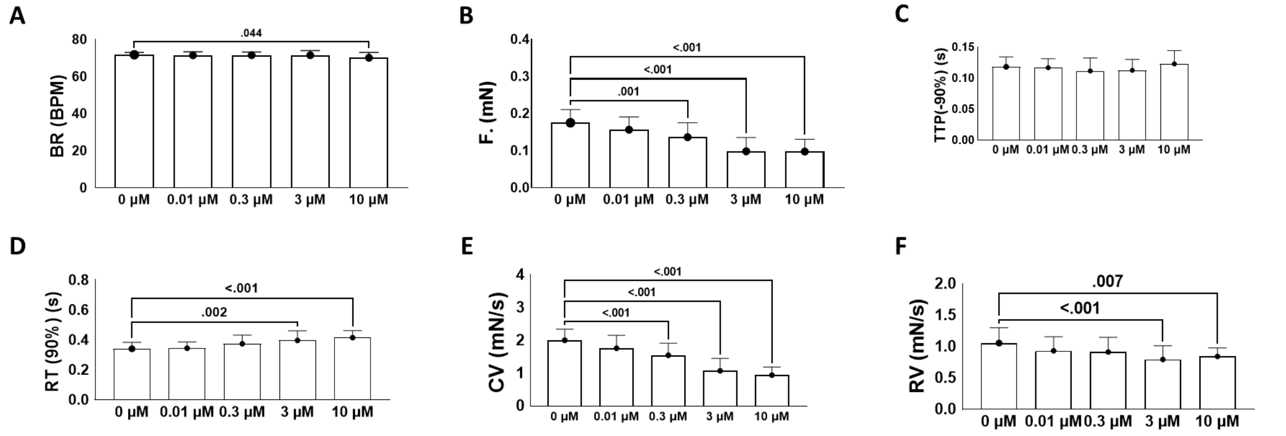

Supplementary Figure S11: Changes in contractile output in EHTiCell2 in response to ryanodine at 1.25 Hz beat rate. Graphs show variation in contractile parameters in response to ryanodine. Responses were acquired in modified Tyrode's solution with 0.6 mM calcium at 1.25 Hz pacing rate. BR = Beat rate, F. = Force, TTP(-90%) = time to peak from 90% contraction, RT(90%) = time to peak from 90% relaxation CV = contraction velocity, RV = relaxation velocity.

Supplementary Figure S12: Only mentioned in discussion.

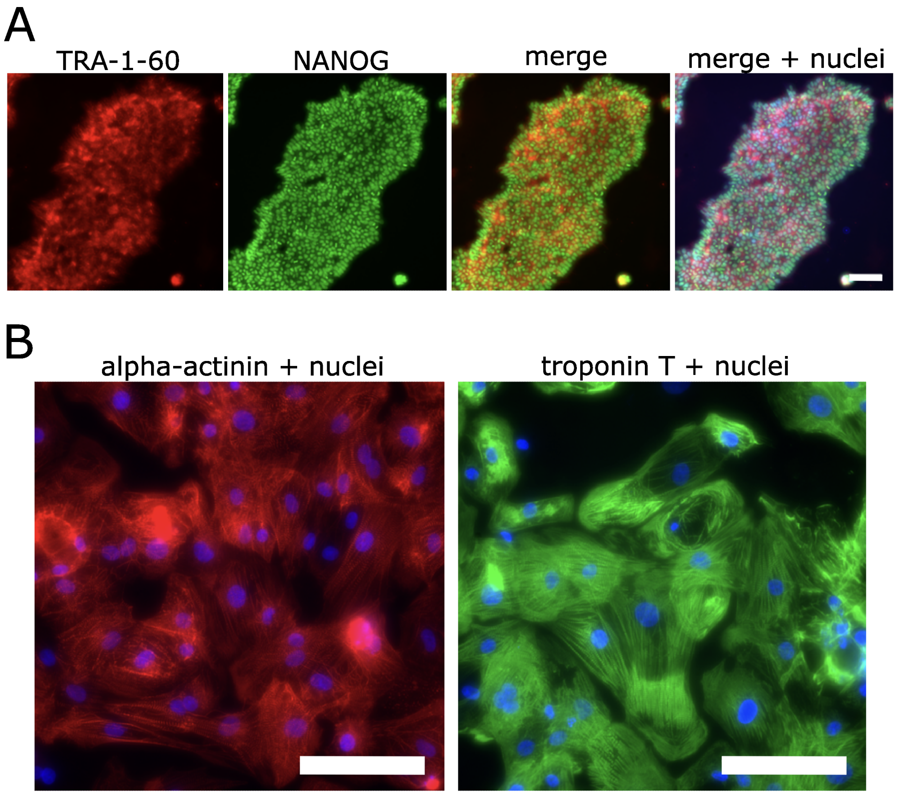

Supplementary Figure S12: Immunocytochemically labeled WTC11 hPSCs (A) and WTC11 hPSC-derived cardiomyocytes (B) in culture after being thawed from storage in liquid nitrogen. The TRA-1-60 cell surface antigen and the transcriptional factor homeobox protein NANOG were labeled in fixed WTC11 hPSCs, while the sarcomeric proteins alpha-actinin and troponin-T were labeled as markers of cardiomyocyte differentiation in fixed WTC11 hPSC-derived cardiomyocytes. Nuclei of cells is represented in blue. Scale bar represents 100 µm.
